# Analysis of Recombinant Characteristics Based on 949 PRRSV-2 Genomic Sequences Obtained from 1991 to 2021 Shows That Viral Multiplication Ability Contributes to Dominant Recombination

**DOI:** 10.1128/spectrum.02934-22

**Published:** 2022-09-08

**Authors:** Xingyang Cui, Dasong Xia, Xinyi Huang, Yue Sun, Mang Shi, Jianqiang Zhang, Ganwu Li, Yongbo Yang, Haiwei Wang, Xuehui Cai, Tongqing An

**Affiliations:** a State Key Laboratory of Veterinary Biotechnology, Harbin Veterinary Research Institute, Chinese Academy of Agricultural Sciences, Harbin, China; b School of Medicine, Shenzhen Campus of Sun Yat-sen University, Sun Yat-sen University, Shenzhen, China; c Department of Veterinary Diagnostic and Production Animal Medicine, College of Veterinary Medicine, Iowa State University, Ames, Iowa, USA; d Heilongjiang Provincial Key Laboratory of Veterinary Immunology, Harbin Veterinary Research Institute, Chinese Academy of Agricultural Sciences, Harbin, China; Changchun Veterinary Research Institute

**Keywords:** PRRSV-2, recombination, recombination hot spots, viral multiplication

## Abstract

Porcine reproductive and respiratory syndrome (PRRS) is one of the most economically important diseases affecting the pig-raising industry. The PRRS virus (PRRSV) has high genetic diversity, partly owing to viral recombination. Some individual recombinant type 2 PRRSV (PRRSV-2) strains have been detected; however, the sequence composition characteristics of recombination hot spots and potential driving forces for recombinant PRRSV-2 are still unreported. Therefore, all available genomic sequences of PRRSV-2 (*n* = 949, including 29 genomes sequenced in this study) from 11 countries from 1991 to 2021 were collected and analyzed. The results revealed that the dominant major recombinant parent has been converted from lineage 3 (L3) to L1 since 2012. The recombination hot spots were located at nucleotides (nt) 7900 to 8200 (in NSP9, encoding viral RNA-dependent RNA polymerase) and nt 12500 to nt 13300 (in ORF2-ORF4, mean ORF2 to ORF4); no AU-rich characteristics were found in the recombination hot spots. Based on infectious clones of L1 and L8 PRRSV-2, recombinant PRRSVs were generated by switching complete or partial NSP9 (harboring the recombination hot spot). The results showed that recombinant PRRSVs based on the L1 backbone, but not the L8 backbone, acquired a higher replication capacity in pig primary alveolar macrophages. These findings will help to understand the reason behind the dominance of L1-based recombination in PRRSV-2 strains and provide new clues for an in-depth study of the recombination mechanism of PRRSV-2.

**IMPORTANCE** Recombination is an important driver of the genetic shifts that are tightly linked to the evolution of RNA viruses. Viral recombination contributes substantially to the emergence of new variants, alterations in virulence, and pathogenesis. PRRSV is genetically diverse, partly because of extensive recombination. In this study, we analyzed interlineage recombination based on available genomic sequences of PRRSV-2 from 1991 to 2021. The study revealed the temporal and geographical distribution of recombinant PRRSVs and the recombination hot spot’s location and showed that artificially constructed recombinant PRRSVs (harboring a high-frequency region) had more viral genomic copies than their parental virus, indicating that dominant recombination was shaped by a tendency to benefit viral replication. This finding will enrich our understanding of PRRSV recombination and provide new clues for an in-depth study of the recombination mechanism.

## INTRODUCTION

Porcine reproductive and respiratory syndrome (PRRS) is characterized by reproductive failure in sows and respiratory symptoms in pigs and causes huge economic losses in the global pig-raising industry (1). The PRRS virus (PRRSV), the cause of PRRS, is an enveloped positive-stranded RNA virus belonging to the order *Nidovirales* and the family *Arteriviridae* (2). PRRSVs are divided into two genotypes: PRRSV-1 (*Betaarterivirus suid* 1), represented by the LV strain and first isolated in the Netherlands in 1991, and PRRSV-2 (*Betaarterivirus suid* 2), represented by the VR-2332 strain and first isolated in the United States in 1992 (3–5). The genomic sequence homology between PRRSV-1 and PRRSV-2 is approximately 60% (6). The PRRSV genome is approximately 15 kb in length with a 5′-untranslated region (UTR) and a poly(A) tail at the 3′ terminus, which contains at least 11 open reading frames (ORFs), including ORF1a, ORF1b, ORF2a, ORF2b, ORF3-7, ORF5a, and NSP2(TF) (7, 8). Owing to the significant genetic variations in intragenotype PRRSVs, PRRSV-1 and PRRSV-2 were classified into several lineages based on the *ORF5* gene (6). PRRSV-2 strains were classified into nine lineages—lineages 1 to 9 (L1 to L9)—with an interlineage diversity of at least 11% (9).

PRRSVs have extremely high genetic diversity owing to nucleotide mutation, insertion, or deletion of genomic fragments and inter- or intralineage recombination (10). When two viruses coinfect the same cell, recombination between the two different viral genomes may occur through the exchange of genetic material, usually leading to the production of a hybrid viral genome. RNA recombination can repair damage to the viral genome during transcription and replication, eliminate harmful mutations, and maintain the genetic characteristics of the virus population (11). Recombinant viruses may be highly virulent and transmissible (10, 12, 13), promoting viral escape from immune protection (14, 15), expanding the host range, and affecting the viral disease epidemic (16). Live attenuated vaccine viruses can acquire pathogenicity via recombination (17).

PRRSV recombination occurs frequently in intra- and/or interlineages, leading to the generation of novel PRRSV variants and alteration of viral pathogenicity. Recombination between wild-type PRRSVs has occurred frequently in recent years, such as the recombination between L1 and L8 or between L3 and L8 PRRSVs (18). Recombination was also reported between wild-type PRRSV and modified live vaccine (MLV) strains (19–21), and the pathogenicity of the recombinant strains to piglets was significantly enhanced (22, 23). More importantly, recombination between two PRRS MLV strains was found in France and Denmark, and the resulting recombinant strains had relatively high virulence in pigs (18). Therefore, recombination of PRRSVs alters the viral pathogenicity in pigs, enhances the risk of MLV vaccines, causes difficulties in viral detection, and makes the PRRS epidemic more complex.

Although many individual recombinant PRRSVs have been isolated (10, 21–25), the systematic recombinant characteristics, including the temporal and geographic distribution, characteristics of the recombination hot spot regions, and the contribution of the recombination hot spot regions to the multiplication of recombinants, remain unclear. In the present study, all available complete or nearly complete genomic sequences (*n* = 949) of PRRSV-2 during the period from 1991 to 2021 were collected, interlineage recombination characteristics were analyzed, and the replicative ability of artificially constructed recombinant PRRSVs was assessed.

## RESULTS

### Genomic surveillance of the 949 PRRSV-2 strains.

**FIG 1:**
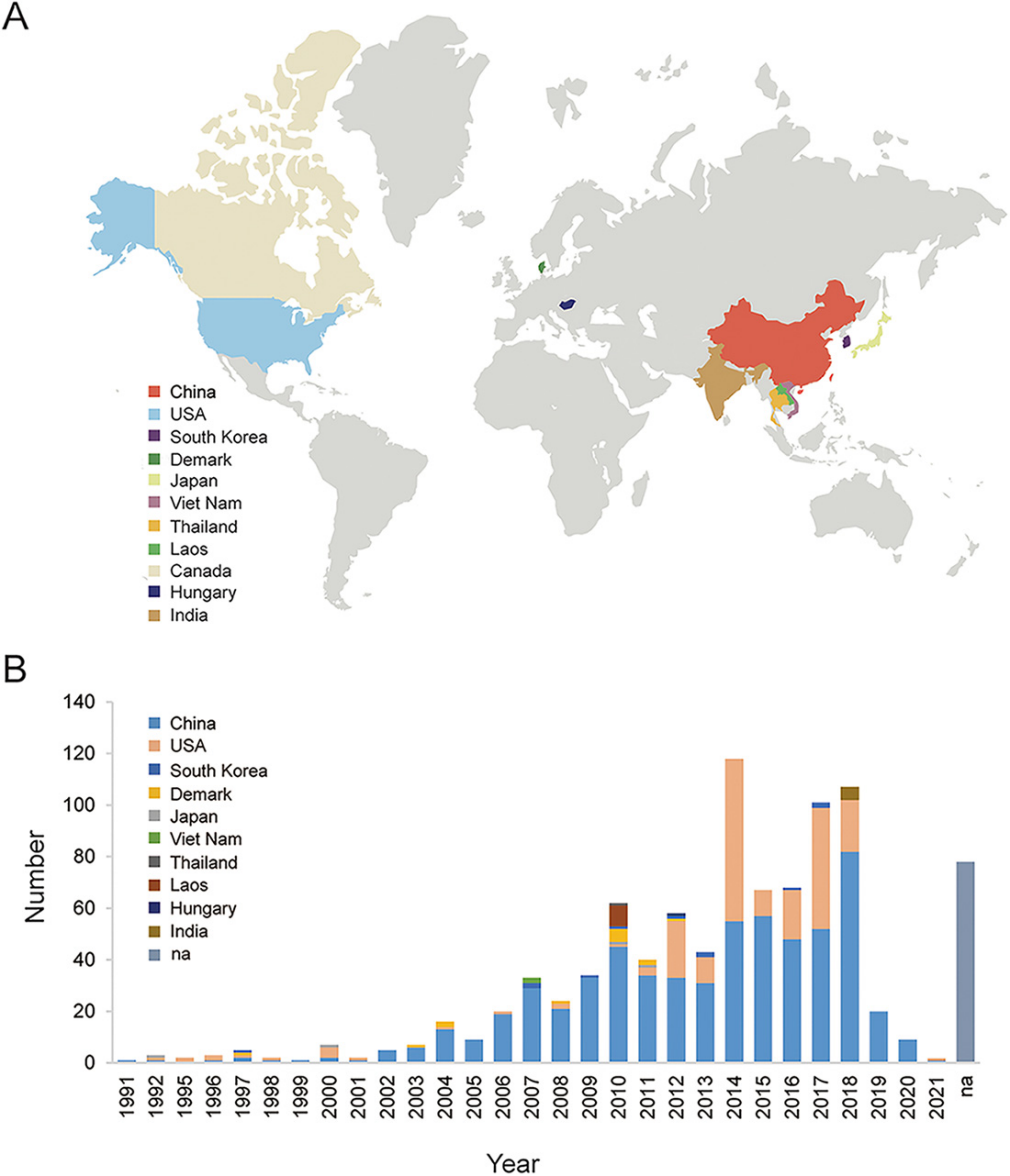
Geographical and temporal distribution of the 949 PRRSV-2 genomes from 1991 to 2021. (A) Geographical distribution of the 949 PRRSV-2 strains collected from 11 countries. (B) Annual number of 949 PRRSV-2 strains. Different colors indicate different countries, including China (*n* = 612), the United States (*n* = 213), South Korea (*n* = 11), Denmark (*n* = 13), Japan (*n* = 4), Viet Nam (*n* = 2), Thailand (*n* = 1), Laos (*n* = 8), Hungary (*n* = 1), India (*n* = 5), and Canada (*n* = 1). The strains lacking collection date information are included in the “na” column.

The genome sizes of 29 PRRSV-2 strains sequenced in this study ranged from 14,625 to 15,898 nt, excluding poly(A). In addition, 920 complete or nearly complete sequences of PRRSV-2 strains were downloaded from GenBank, which were collected from 1991 to 2021. Collectively, the 949 PRRSV-2 sequences included 797 complete genomic sequences (83.98%) and 152 nearly complete genomes (>14 kb, 16.02%). The 949 PRRSV-2 strains were distributed mainly in Asia, North America, and Europe (Fig. 1A). The strains were collected from at least 11 countries, most of which were from the United States (*n* = 225) and China (*n* = 639). The collection dates of 871 PRRSV-2 strains (91.78%) were available. The number of sequences showed an increasing trend since 2006, within the range of 24 to 118 from 2007 to 2018 (Fig. 1B).

### Lineage classification of the 949 PRRSV-2 strains.

**FIG 2:**
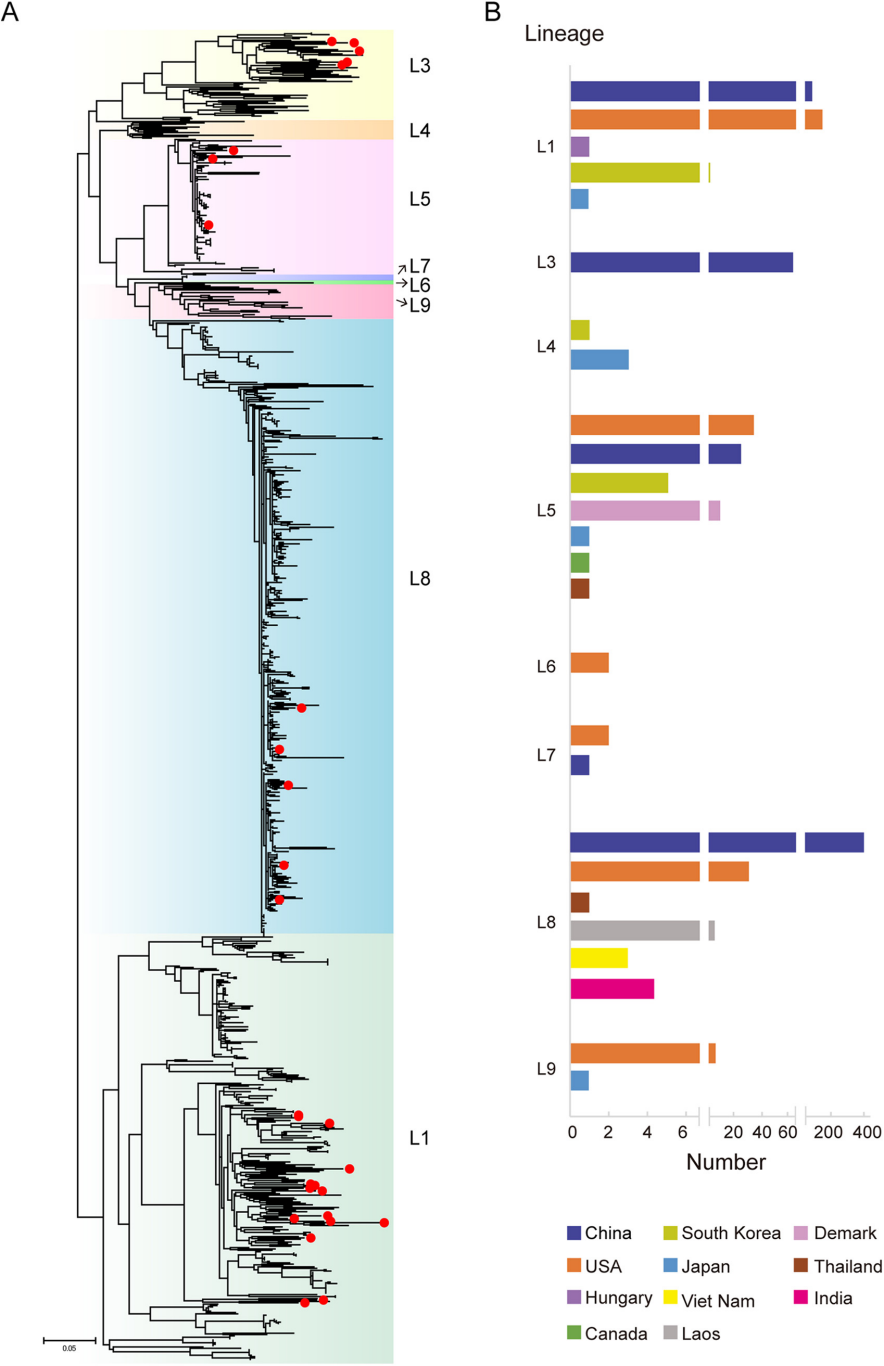
Lineage classification of the 949 PRRSV-2 strains. (A) Phylogenetic tree based on *ORF5* sequence of 949 PRRSV-2 strains. An ML-tree was generated using general time-reversible substitution as a model, and reliability was assessed by bootstrap analysis of 1,000 replications. The twenty-nine PRRSV-2 strains sequenced in this study are marked by red dots. (B) Distribution of PRRSV lineages in different countries, based on the 949 PRRSV-2 strains.

Phylogenetic analysis was performed based on *ORF5* sequences extracted from the 949 PRRSV-2 sequences. Some L4 (*n* = 10) and L9 (*n* = 14) strains were added as reference sequences to enhance the reliability of the classification. The results showed that the PRRSV-2 strains were classified into eight different lineages: L1 (*n* = 329), L3 (*n* = 68), L4 (*n* = 4), L5 (*n* = 83), L6 (*n* = 2), L7 (*n* = 3), L8 (*n* = 449), and L9 (*n* = 11) (Fig. 2A). Most PRRSVs belonged to L8 (represented by JXA1), L1 (represented by NADC30), and L5 (represented by VR-2332). L5 was the most widely distributed lineage in seven countries in Asia, North America, and Europe (Fig. 2B). L8 and L1 PRRSVs were distributed in six and five countries, respectively. Six lineages were in the United States, and five were in China. China preceded the United States in L1 and L8, whereas the United States preceded China in L5 and L7. The 29 PRRSV-2 strains sequenced in this study were clustered into four lineages: L1 (*n* = 15), L3 (*n* = 5), L5 (*n* = 4), and L8 (*n* = 5).

### Interlineage recombination analysis of PRRSV-2 strains.

**FIG 3:**
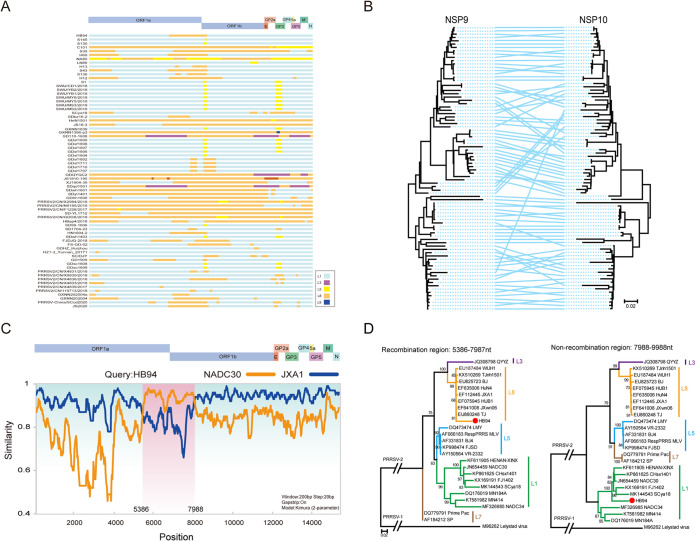
Interlineage recombination analysis of PRRSV-2 strains. (A) Map of major or minor parental lineages of recombinant PRRSV-2 strains collected from 2019 to 2021. Different colors indicate different lineages. The positions are in accordance with the PRRSV VR-2332 strain. (B) Comparison of the topological structure of phylogenetic trees of NSP9 and NSP10. A line connects genes from the same strains. The lines crossed with a large span indicate potential recombination. (C) Simplot analysis of a representative PRRSV-2 HB94 strain. The major parent is JXA1, and the minor parent is NADC30; the HB94 genome is divided by two breakpoints, and the recombinant region is shaded pink. (D) Phylogenic trees were constructed based on the regions from nt 5386 to 7987 and nt 7988 to 9988.

Recombination analysis of PRRSV-2 strains was performed using nine representative parental strains from different PRRSV-2 lineages. Of 29 PRRSV-2 strains sequenced in clinical samples, 13 were interlineage recombinant, and 58 of 179 PRRSV-2 strains collected from GenBank were identified as recombinants, which included global PRRSV-2 strains from 1991 to 2021, except for China and the United States from 1991 to 2018. (The recombination of PRRSV-2 from 1991 to 2018 in China and the United States was analyzed in our previous study [24], a total of 179 PRRSV-2 sequences were newly collected from GenBank in this study for recombination analysis, and the data set of PRRSV-2 from China and the United States between 1991 and 2018 is integrated into this study.) In most of the recombinants, the major parent strain belonged to L1 PRRSV (Fig. 3A), indicating that L1 PRRSVs provide a backbone in most recombinant events. The L8 PRRSVs were the second most dominant major parents for recombination, and only a few L5 PRRSVs were the major parents (Fig. 3A). The positions of recombinant breakpoint were distributed throughout the whole PRRSV genome; however, many breakpoints were concentrated in the NSP9 region (Fig. 3A). The phylogenetic trees based on NSP9 and NSP10 demonstrated significant topological heterogeneity, which could be attributed to the recombination events (Fig. 3B). For instance, the HB94 strain sequenced in this study was a recombinant PRRSV, with JXA1-like PRRSV (L8) as the major parent and NADC30-like PRRSV (L1) as the minor parent, and the recombination region was located between nt 5386 and 7988 (Fig. 3C). Based on the recombinant region, a phylogenetic tree was constructed based on both sides of the recombinant breakpoint (Fig. 3D). The results showed that the recombinant region from nt 5386 to nt 7987 of HB94 was located in the L8 clade of the phylogenetic tree, whereas the nonrecombinant region from nt 7988 to nt 9988 of HB94 belonged to the L1 clade in the phylogenetic tree, indicating that a recombination event occurred in the PRRSV HB94 strain.

### Temporal and geographic distribution of recombinant PRRSV-2 strains.

**FIG 4:**
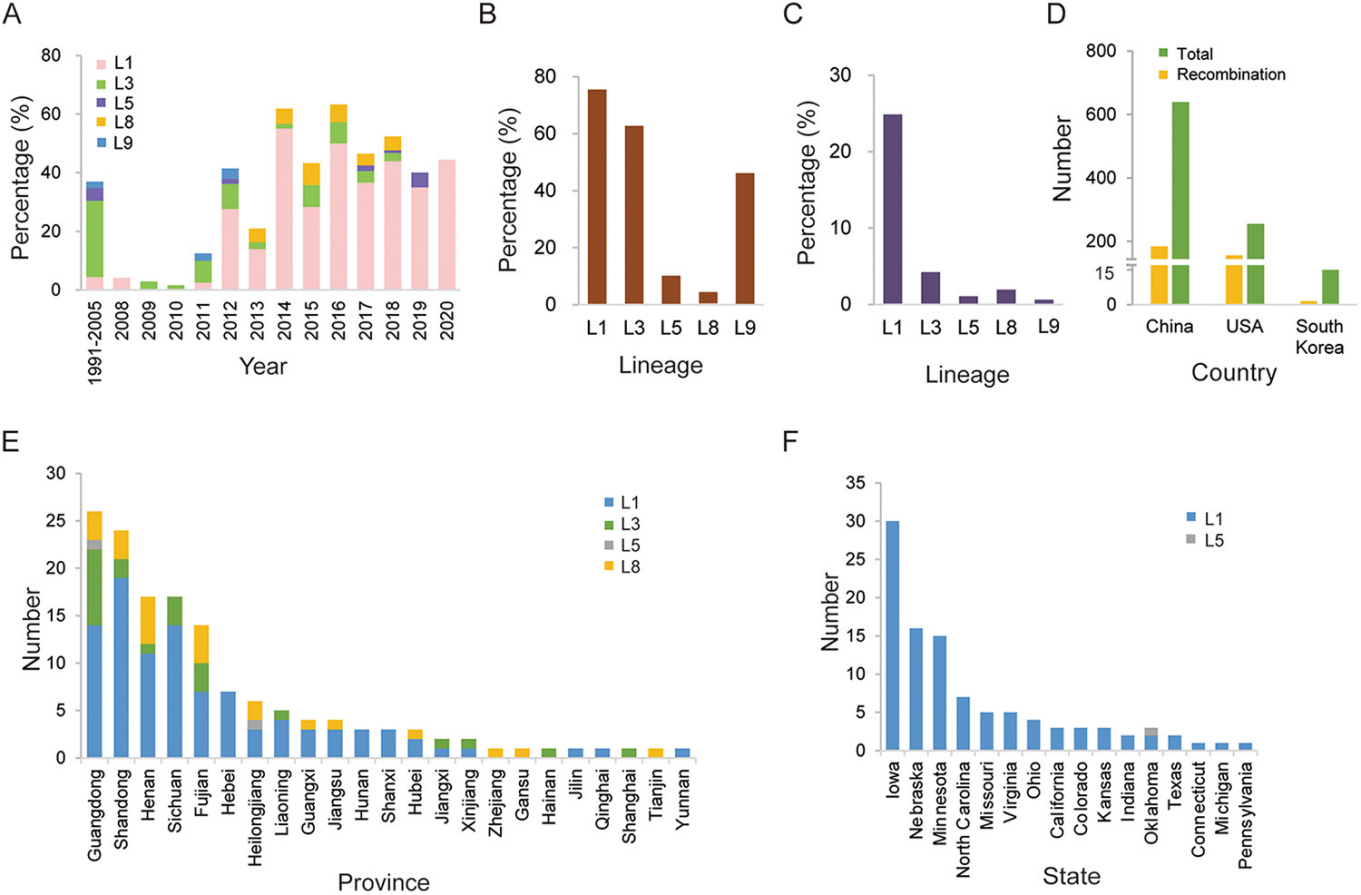
Distribution of recombinant PRRSV-2 strains. (A) Annual proportion of the recombinant PRRSVs from different lineages to the total number of PRRSVs. (B) Proportions of the recombinant PRRSVs in each lineage out of the total PRRSVs in each lineage. (C) Proportions of the recombinant PRRSVs in each lineage out of the total number of PRRSVs. (D) Numbers of recombinant and total PRRSV-2 strains in China and the United States. (E and F) Geographical distribution of recombinant PRRSV-2 strains in different provinces/states in China and the United States, respectively.

By consolidating the recombination data of this and our previous study (24), recombination events were detected in 338 PRRSV-2 strains, and the dominant lineage was found to be L3 PRRSV between 1991 and 2005, which was replaced by L1 PRRSV since 2012 (Fig. 4A), although the L5 and L8 PRRSVs were abundant and widely distributed in numerous countries from 1991 to 2021. For individual lineages, L1 PRRSV occupied the highest recombinant proportion among all lineages; for example, 257 recombinants were detected for L1 PRRSVs (257/326,78.83%), including major or minor recombinant parents. For L3 PRRSVs, 42 of 68 strains (61.76%) were recombinant, whereas L8 PRRSVs had the lowest recombinant ratio, with 26 of 449 (5.79%) being recombinant (Fig. 4B). Among all lineages, L1 had the highest proportion (257/949, 27%) of all recombinant strains from the 949 PRRSV-2 strains (Fig. 4C). No recombination was detected in L4, L6, and L7 PRRSVs. The recombinant PRRSV-2 strains were geographically distributed in three countries: China (*n* = 179), the United States (*n* = 158), and South Korea (*n* = 1) (Fig. 4D). The recombination rate in the United States (61.96%) was significantly higher than in China and South Korea (28.01 and 7.69%, respectively). A detailed distribution of recombinants revealed the provinces Guangdong, Shandong, and Henan in China (Fig. 4E) as the top three areas with the recombinants, while the top three states in the United States were Iowa, Nebraska, and Minnesota (Fig. 4F). Three main recombinant lineages—L1, L3, and L8—were found in China; however, the main recombinant lineage in the United States was L1 only, and the lineage distribution in the United States was relatively simplistic.

### Yearly distribution of PRRSV-2 recombinants in the United States and China.

**FIG 5:**
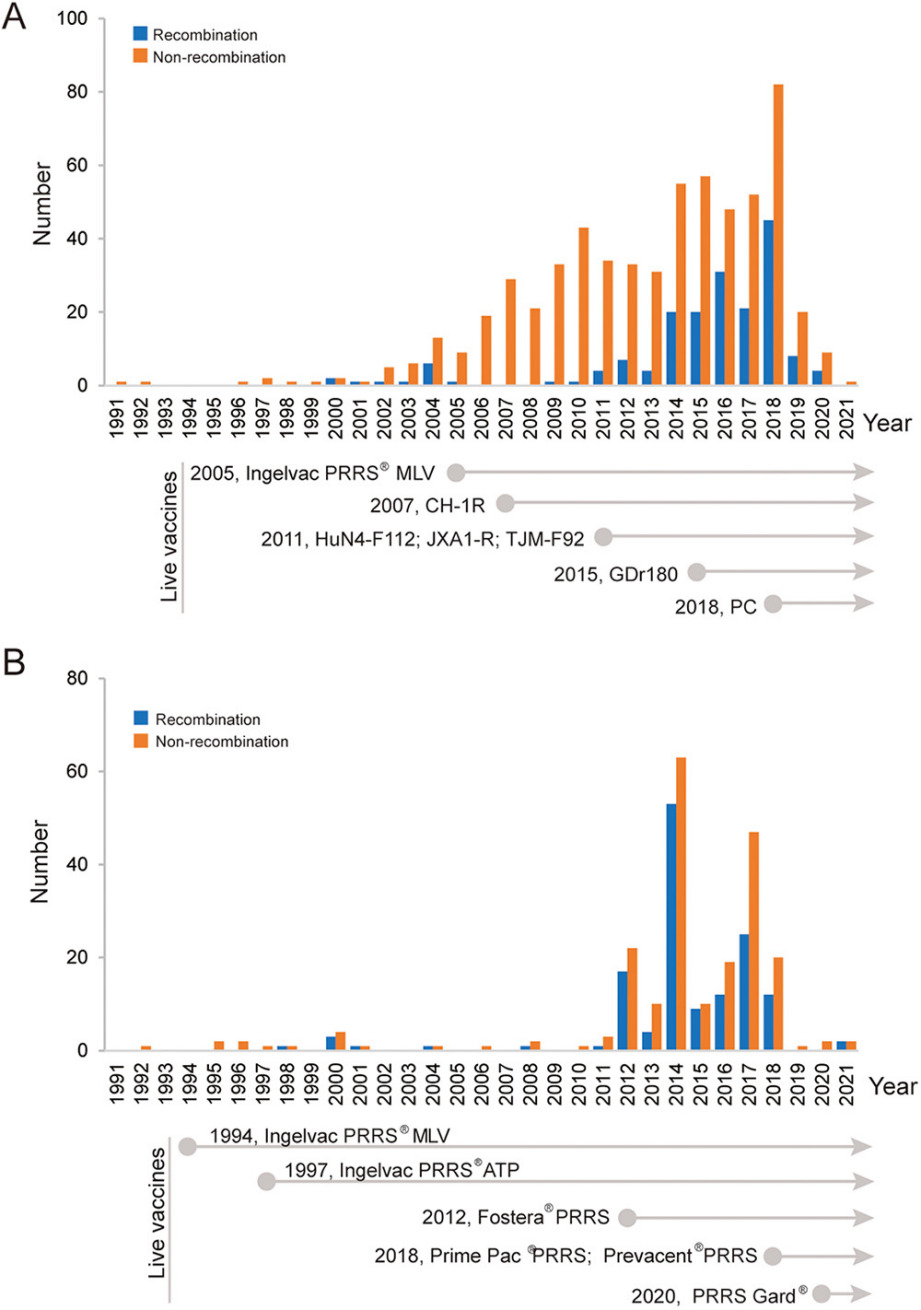
Temporal distribution of PRRSV-2 recombinants and PRRSV-2 vaccines in China and the United States. The annual number of recombinant PRRSVs and the year of approval of PRRS vaccines in China (A) and the United States (B) are indicated.

The year-to-year distribution of PRRSV-2 recombinants in the United States and China was also investigated. In China, PRRSV-2 recombinant was first detected in 2000, and only a few recombinant PRRSV-2 strains were detected from 2001 to 2013. In 2014, the number of recombinant PRRSV-2 strains and recombinant ratio increased significantly (Fig. 5A). In the United States, the recombinant PRRSV-2 strain was first detected in 1998 and remained at a very low level until 2011. Since 2012, the number of recombinant PRRSV-2 strains significantly increased, and the overall recombination rate has been higher than that in China (Fig. 5B).

### Fine mapping of high-frequency recombinant hot spots.

**FIG 6:**
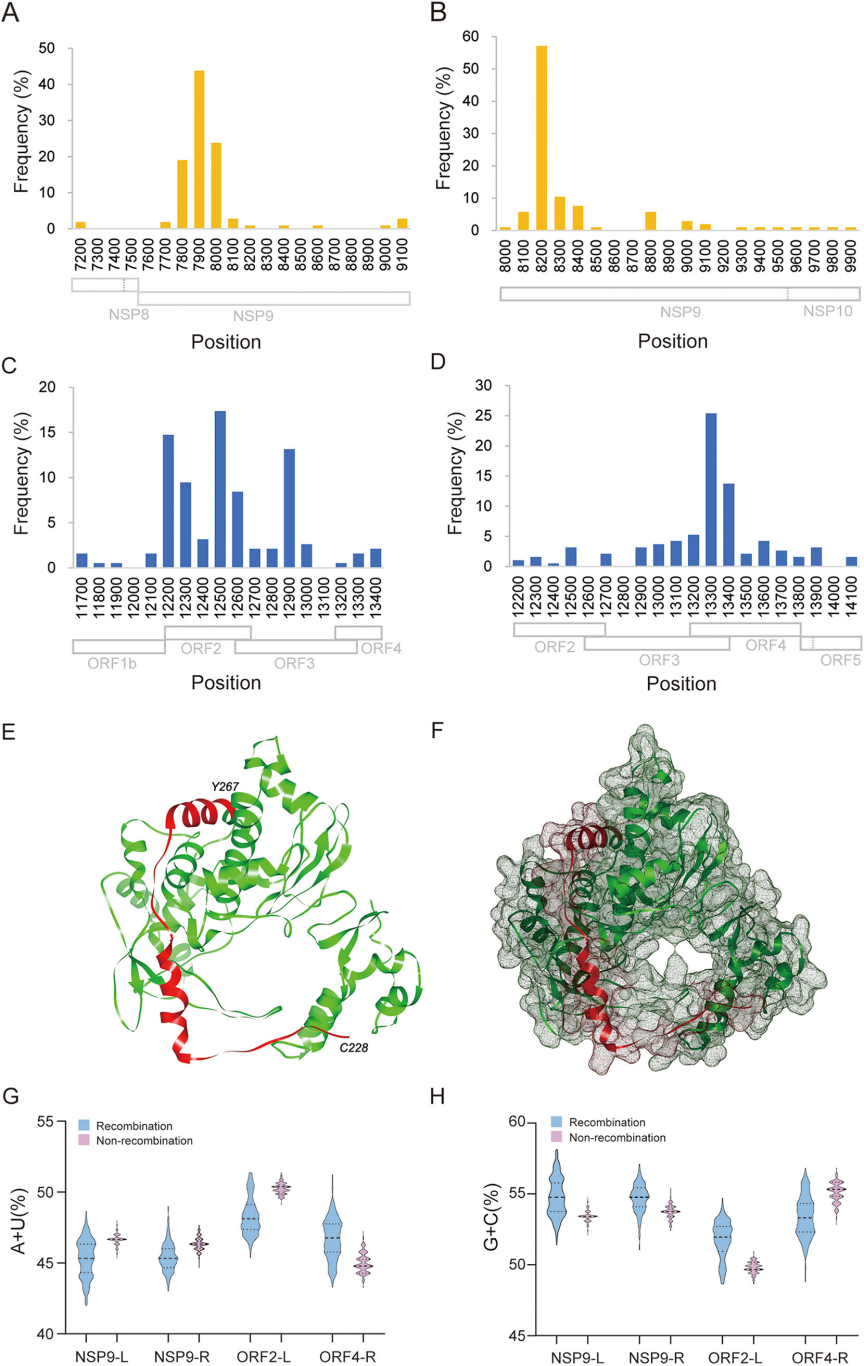
Characteristic of recombination hot spots. (A and B) Frequency of left or right breakpoints in recombinant hot spots in NSP9, respectively. The recombinant hot spot is mainly concentrated at the region between nt 7800 and nt 8200. (C and D) Frequency of left or right breakpoints in recombinant hot spots in ORF2-ORF4, respectively. The detailed position is mainly concentrated between nt 12500 and nt 13300. (E and F) Location of residues Cys228 to Tyr267, the recombination hot spot in RdRp. PRRSV RdRp structure was predicted with the Swiss-Model server, and the RdRp structure (PDB ID 6xqb, chain A) of SARS-CoV-2 was the template for building PRRSV RdRp model. (G) AU contents in the high frequency left or right breakpoints. NSP9-L, nt 7800 to 8000; NSP9-R, nt 8100 to 8400; ORF2-L, nt 12200 to 12900; ORF4-R, nt 13100 to 13400. The numbers of recombinant and nonrecombinant strains are 323 and 603, respectively. (H) GC contents in high-frequency left or right breakpoints.

The locations and frequencies of interlineage recombination were calculated. Two high-frequency recombinant hot spots were found in NSP9 and ORF2-ORF4 (mean ORF2 to ORF4) of PRRSV-2. In NSP9, the left and right breakpoints were mainly concentrated at nt 7800 to nt 8000 and at nt 8200 to nt 8400, respectively (Fig. 6A and B), referring to the nucleotide position of VR-2332. More than 40% of the recombinant breakpoints were located around nt 7900 and nt 8200. In ORF2-ORF4, the left and right breakpoints were at nt 12200 to 12600 and nt 13200 to 13400, respectively (Fig. 6C and D). The three-dimensional (3D) structure of the RNA-dependent RNA polymerase (RdRp; encoded by NSP9) of PRRSV-2 was predicted. The RdRp structure (PDB ID 6xqb) of SARS-CoV-2, another member of the same *Nidovirales*, shared the highest global model quality estimation (GMQE) score (0.32) and was selected for homologous modeling. In the predicted structure of PRRSV RdRp, the amino acids at positions 228 to 267 (corresponding to nt 682 to 801 in NSP9, located within recombinant hot spots) were located near the pocket of RdRp and may be related to the binding of viral RNA (Fig. 6E and F).

### Nucleotide composition characteristics and secondary structure of high-frequency recombinant regions.

The AU base content is considered one of the factors affecting viral recombination. In this study, the recombination breakpoint positions of all recombinant strains were counted, and recombination breakpoints were mainly concentrated in two regions, namely, NSP9 and ORF2-ORF4. The recombination breakpoints located near the NSP9 and ORF2-ORF4 regions were collected, it was found that the recombination hot spots were mainly clustered in the range of 200 nt on both sides of NSP9 and ORF2-ORF4, then AU contents of these four nucleotide fragments harboring recombination breakpoints in NSP9 or ORF2-ORF4 was analyzed. The AU contents of NSP9-L, NSP9-R, and ORF2-L in recombinant PRRSV-2 strains were slightly lower than those of the nonrecombinant PRRSV-2 strains, but the difference was not statistically significant (Fig. 6G). Nucleotide composition analysis based on NSP9 revealed that the mean AU contents were 47.3 and 47.1% for the recombinant and nonrecombinant PRRSV-2 strains, respectively. A similar AU content was found in ORF2-ORF4, suggesting that the AU content in these regions was not significantly different. A similar GC content was observed between the recombinant and nonrecombinant PRRSV-2 strains (Fig. 6H). Furthermore, AU-tracts were examined, and the 20-nt sequences on both sides of the breakpoint with more than three AU-tracts were counted. Statistical analysis results showed that only 7 of 56 recombinant strains had this characteristic, accounting for a small proportion.

The left and/or right recombination breakpoints were mapped to the predicted RNA secondary structure of the NSP9 and ORF2-ORF4. According to the results, 16 of 31 recombination breakpoints in NSP9 were located at a separate stem-loop structure, and 8 of 25 recombination breakpoints in ORF2-ORF4 were located at a separate stem-loop structure (see Fig. S1).

### Generation and characterization of artificial NSP9 recombinant L1 or L8 PRRSVs.

**FIG 7:**
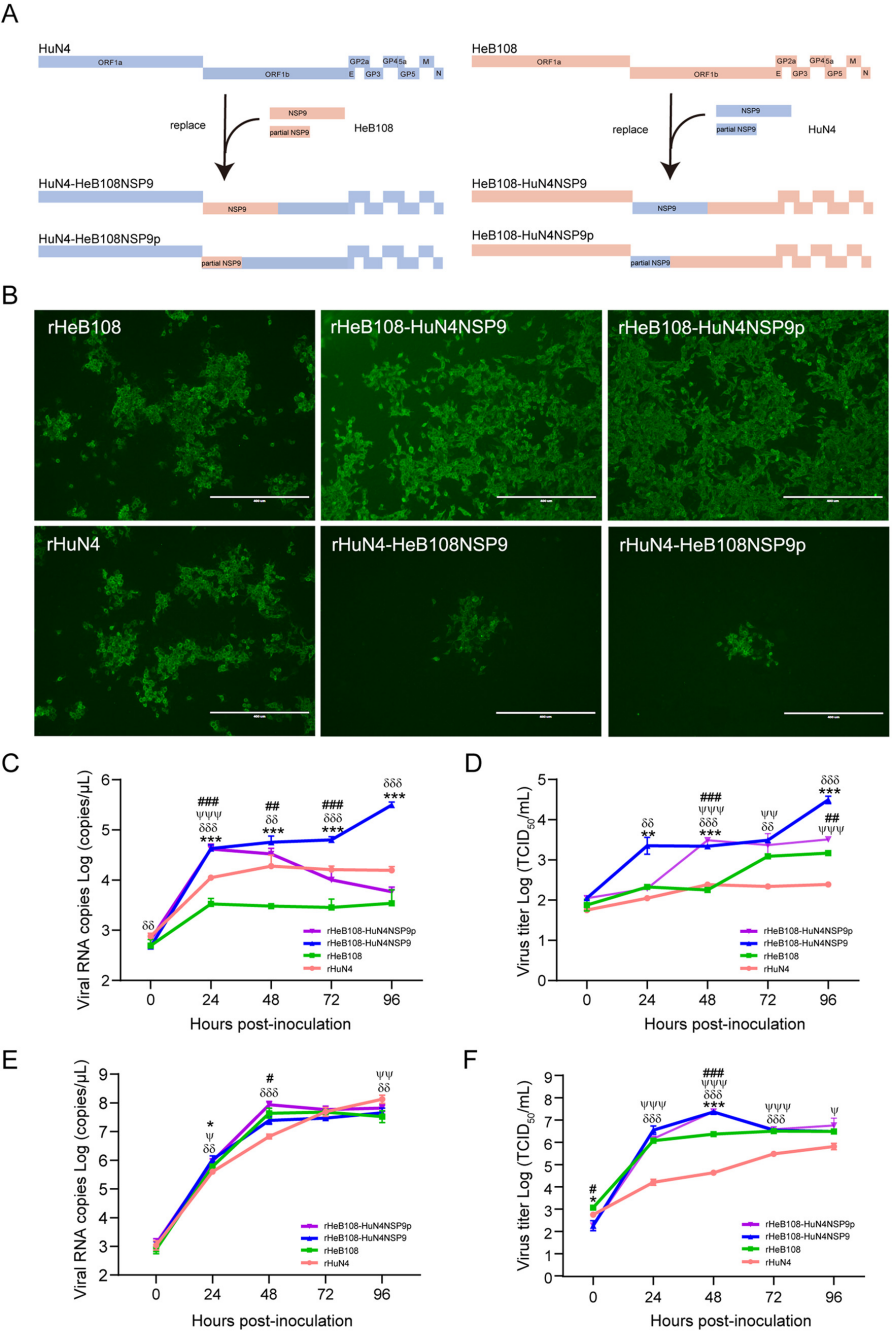
Generation and characterization of artificially recombinant PRRSVs. (A) Schematic diagram for constructing recombinant plasmids. (B) Virus detection by immunofluorescence. Recombinant and parental plasmids (4 μg) were transfected into Marc-145 cells. At 5 days posttransfection, the transfected cells were fixed and stained with monoclonal antibody 3F7 against PRRSV, followed by incubation with Alexa Fluor 488-conjugated goat anti-mouse IgG. Scale bar, 400 μm. The growth kinetics of the recombinant (rHeB108-HuN4NSP9 and rHeB108-HuN4NSP9p) and parental viruses in PAMs (C and D) and Marc-145 cells (E and F) are represented. Each data point represents the mean value of three replicates with the SD. *, Significant difference between rHeB108 and rHeB108-HuN4NSP9 (*, *P < *0.05; **, *P < *0.01; ***, *P < *0.001); #, significant difference between rHeB108 and rHeB108-HuN4NSP9p (#, *P < *0.05; ##, *P < *0.01; ###, *P < *0.001); δ, significant difference between rHuN4 and rHeB108-HuN4NSP9 (δ, *P < *0.05; δδ, *P < *0.01; δδδ, *P < *0.001); ψ, significant difference between rHuN4 and rHeB108-HuN4NSP9p (ψ, *P < *0.05; ψψ, *P < *0.01; ψψψ, *P < *0.001).

Four recombinant PRRSVs were artificially constructed by switching the complete or partial NSP9 (harboring the recombinant hot spot) based on the infectious clones of L1 PRRSV HeB108 and L8 PRRSV HuN4, respectively (Fig. 7A). Four recombinant PRRSVs (rHeB108-HuN4NSP9, rHeB108-HuN4NSP9p, rHuN4-HeB108NSP9, and rHuN4-HeB108NSP9p), as well as their parent viruses, rHeB108 and rHuN4, were rescued in transfected cells. All six rescued viruses were confirmed by immunofluorescence assay (IFA) (Fig. 7B) and proliferated on cells for three passages. As a result, rHeB108-HuN4NSP9 and rHeB108-HuN4NSP9p and their parent viruses proliferated rapidly; however, the rHuN4-HeB108NSP9 and rHuN4-HeB108NSP9p viruses failed to proliferate because of weak replication, although they could be rescued.

Two recombinant PRRSVs (rHeB108-HuN4NSP9 and rHeB108-HuN4NSP9p) and their parental strains (rHeB108 and rHuN4) were used to compare their replicative capability in primary alveolar macrophages (PAMs) and Marc-145 cells. In PAMs, growth kinetics showed that the two recombinant viruses based on the rHeB108 backbone had higher viral genomic copies than their parents rHeB108 or rHuN4 (*P < *0.01) (Fig. 7C). The copy numbers of rHeB108-HuN4NSP9 or rHeB108-HuN4NSP9p were significantly higher than those of the parent viruses for 72 to 96 h postinfection (hpi) (*P < *0.001), and the titers of the two recombinant viruses were higher than those of the parent viruses for 48 to 96 hpi (*P < *0.01) (Fig. 7D). In Marc-145 cells, the recombinant PRRSVs and their parental strains reached peak replication levels in copy number and titer at 48 hpi. The copy number and viral titer of rHeB108-HuN4NSP9p were significantly higher than that of rHeB108 at 48 hpi (*P < *0.05 and 0.001, respectively) (Fig. 7E and F).

## DISCUSSION

PRRS is one of the most important swine diseases worldwide and has caused significant economic losses to the global swine industry since it was first reported in 1987. Among the 10 top pig-raising countries or regions (China, European Union, the United States, Brazil, Russia, Vietnam, Canada, Philippines, South Korea, and Mexico), PRRS has been reported in all countries except Brazil (26). PRRSV has a high recombination frequency, which results in the emergence of novel PRRSV variants (10, 27, 28). In recent years, more than 10 wild-type PRRSV recombination cases and several PRRS MLV strain recombination cases have occurred in North American, European, and Asian countries (21–23, 25). Although some individual PRRSV recombinants have been reported, a systematic overview of PRRSV recombination is still lacking, and the potential factors associated with PRRSV recombination have not been reported.

To investigate the recombinant characteristics of PRRSV-2, 949 PRRSV-2 sequences were collected and analyzed. Most of them belonged to L8 and L1, followed by L5 and L3. In recent years, L1 has been a dominant and prevalent lineage in the United States and China (24, 29). The most widely distributed lineage was L5, whose strains remained some of the major circulating strains. However, L5 PRRSV was not the dominant recombinant PRRSV-2. L1 has a high recombination frequency, mainly because it is one of the dominant local strains with low mortality in pigs and can exist in pig herds for a long time.

The geographical distribution of recombinant PRRSV-2 strains revealed that the recombination rate is related to the density of pigs. Guangdong, Shandong, and Henan, the main pig-raising provinces in China, were detected as the top three regions with regard to the number of recombinants present. The same phenomenon appeared in the United States. This suggests that a high density of pig populations facilitates coinfection of different PRRSVs and increases the possibility of generating recombinant PRRSVs. PRRSVs of different lineages have coexisted for a long time, which makes them easier to spread at the same pig farm. Intersector spread is enhanced by the movement of feeder pigs, spatial adjacency of sectors, and farm density in the destination sector (26), which could further increase the frequency of recombination between different lineages through wide-range transmission.

Vaccines against PRRS-2 have been commercially available since 1994 (30) and are now widely used; however, they offer limited cross-protection against heterologous PRRSVs and fail to clear PRRSVs *in vivo* (24, 31, 32). Immune pressure contributes to the evolution of the virus; moreover, recombination between MLV and wild-type PRRSV strains has been reported (22, 23). We attempted to analyze the possible relationship between PRRSV recombination and the usage of vaccines. Nine vaccines are licensed in China, including one MLV derived from L5 PRRSV (Ingelvac PRRS MLV), seven vaccines derived from L8 PRRSV (five MLV vaccines [CH-1R, HuN4-F112, JXA1-R, TJM-F92, and GDr180] and two killed vaccines [CH-1a and JXA1]), and one chimeric live vaccine (PC strain, L5&L8). The PRRS-2 vaccines in the United States are derived from L5, L7, L8, and L1 PRRSVs. However, no obvious correlation between PRRSV recombination and vaccine usage was determined because of the limited background information about vaccine usage in the farms where recombinant PRRSV-2 strains were detected. Therefore, the relationship between PRRS MLV vaccines and recombination remains a problem worthy of consideration in future.

The structure of SARS-CoV-2 RdRp shared the highest matching score to PRRSV RdRp structure; SARS-CoV-2 and PRRSV both belong to the order *Nidovirales* and have the same transcription strategy; therefore, the structure of SARS-CoV-2 RdRp was used as a reference model. In the predicted structure, the amino acid position (i.e., amino acids 228 to 267) of the high-frequency recombination region of PRRSV-2 was located near the pocket of RdRp. These positions may influence the binding of RdRp to viral RNA or influence the template switch of RdRp, leading to viral recombination. Further experiments are required to confirm the role of RdRp in the template-switching process.

Previous studies have reported that recombination is more likely to occur in AU-rich regions (33); in the present study, no AU-rich regions were found in recombinant PRRSV-2 strains. The secondary structure of some RNA viruses has an impact on recombination; the stem-loop or hairpin structure might promote the occurrence of recombination (34, 35). Therefore, secondary structure analysis is necessary to assess the effect of recombination. According to the current data, the secondary structures of recombination breakpoint region were predicted; however, there is no obvious direct correlation between recombination and secondary structure. We further analyzed host adaptability and natural selection with RdRp codon usage of PRRSV. The codon usage bias of RdRp showed that natural selection had a greater effect on recombinant viruses, and the recombinant viruses had a high codon adaptation index to the Sus scrofa genome (data available upon request).

To investigate the possible role of NSP9 in altering viral replication capacity, four recombinant PRRSVs were generated by switching complete or partial NSP9. Two recombinant viruses based on the L8 backbone did not successfully proliferate owing to their low replication ability. The recombinant viruses HeB108-HuN4NSP9 and HeB108-HuN4NSP9p had greater replication ability than the parent strains. In the present study, we also attempted to switch the ORF2-ORF4 between L1 and L8 PRRSV but failed, probably due to ORF2-ORF4 being critical for viral infection. As for the ORF2-ORF4, the ORF3 was overlapped by both ORF2 and ORF4, making it difficult to switch individual complete ORFs. NSP9 (encoding the viral RdRp) is involved in viral replication, while GP2, GP3, and GP4, which form a heterotrimer, are the major determinants of PRRSV infection (36, 37). The recombination of the regions may be associated with increased viral replication capacity and cellular tropism. Such findings enhance our understanding of the reason for the dominance of L1-based recombination in PRRSV-2 strains and provide a basis for in-depth studies of viral recombination.

## MATERIALS AND METHODS

### Cells and viruses.

Marc-145 cells were cultured in Dulbecco modified Eagle medium (DMEM; Sigma-Aldrich, USA) supplemented with 10% fetal bovine serum (FBS), penicillin (100 IU/mL), and streptomycin (100 μg/mL). PAMs were prepared from the lung lavage of 4-week-old and pathogen-free piglets. PAMs were cultured in RPMI 1640 medium (Gibco/Thermo Fisher Scientific, USA) supplemented with 10% FBS and penicillin-streptomycin. The cells were maintained at 37°C and 5% CO_2_. The infectious clones of the PRRSV HuN4 (GenBank no. EF635006) and HeB108 strains (GenBank no. MN046224) were preserved in our laboratory.

### Genomic sequencing of PRRSV-2 in clinical samples.

During 2018 and 2019, clinical samples were collected from 136 cases of pig diseases at different farms in 15 provinces, including Heilongjiang, Shandong, Hebei, and Guangdong, in China. The pathogens were investigated using a high-throughput sequencing approach based on Illumina HiSeq platform, according to previous methods (38). PRRSV-2 sequence reads were assembled using SeqMan program for each sample. Gaps were filled using reverse transcriptase PCR (RT-PCR) and Sanger sequencing, according to previously described methods (24).

### Data sets.

All available complete or nearly complete genomes (length, >14 kb) of PRRSV-2 collected from 1991 to 2021 were downloaded from GenBank on 3 January 2022. The sequences of MLV and cell passage-derived strains and unverified sequences of PRRSV-2 were removed from the data sets. Twenty-nine complete genomes sequenced in this study were added to the data sets. These sequences comprised 949 PRRSV-2 strains distributed across 11 countries (Fig. 1A).

### Lineage classification of the PRRSV-2 strains.

*ORF5* sequences were extracted from 949 genomic sequences of PRRSV-2, aligned by MAFFT (39), and edited using BioEdit software (v7.0.9). A maximum likelihood phylogenetic tree (ML-tree) was constructed using PhyML (v3.0) (40) and then estimated using the model of nucleotide substitution and Subtree Pruning Regrafting (SPR) branch swapping, as previously described. (9). Bootstrap values were calculated for 1,000 replicates (41). The lineage classification was based on a description by Shi et al. (9).

### Recombination analysis.

Potential recombination events, parental strains, and recombination breakpoint analyses were performed as previously described (10, 24, 42). Briefly, nine representative strains were selected as the reference parents: NADC30 (L1), XW008 (L2), MD001 (L3), EDRD-1 (L4), VR-2332 (L5), P129 (L6), SP (L7), JXA1 (L8), and MN30100 (L9). Interlineage recombination events were preliminary detected using SimPlot software (v 3.5) with a window size of 200 bp and a step size of 20 bp, and the recombinant parental strains and breakpoint locations were determined. Then, recombination detection program (RDP; v4.96) was used for further analysis of the recombinant strains identified preliminarily (43). Recombination events were validated using RDP software, which includes RDP, GENECONV, Maxchi, Chimaera, 3Seq, Bootscan, and SiSscan programs, to identify putative recombination sites. Recombination events were considered likely only if they were detected by at least four of the seven programs in the package with a *P* value of ≤0.01. Furthermore, phylogenetic tree construction was conducted based on recombinant fragments to validate the recombination events. The final recombinant strains were confirmed based on the three methods above. Among the 949 PRRSV-2 sequences, the PRRSV-2s collected in the United States and China in between 1991 and 2018 were analyzed in our previous study (24), and the previous data were integrated into the current data set.

### AU-rich analysis in the recombination hot spots.

AU-rich sequences can facilitate homologous recombination (33, 44). Here, two recombination hot spots were identified; therefore, the nucleotide sequences in the recombination hot spots were selected, and the AU content was analyzed. To investigate the effect of base composition on recombinant or nonrecombinant PRRSVs, the nucleotide contents (A%, U%, G%, and C%) of NSP9 and ORF2-4 were calculated using BioEdit and Lasergene software (v7.1). Moreover, sequences that may have had the same ancestor were excluded during the recombination breakpoint analysis, and the methods of screening strains have been described in detail in a previous study (24).

### Secondary structure prediction in recombination hot spots.

To explore the relationship between the recombinant breakpoint and secondary structure, the complete RNA secondary structures of the NSP9 and ORF2-ORF4 were predicted. Considering the parents of most recombinant strains were L1 and L8 strains, the sequences of representative strains NADC30 and JXA1 (accession numbers JN654459 and EF112445) of L1 and L8 PRRSVs were selected for secondary structure prediction. The Mfold web server (http://www.unafold.org/) was used to predict the RNA secondary structure (45). Subsequently, the recombination breakpoints were mapped to the corresponding positions of the prediction structure according to a previous method (34).

### Homology modeling and location of the recombination hot spot in RdRp.

The 3D structure of PRRSV RdRp was predicted through homology modeling based on the sequence of the HuN4 strain by using SWISS-MODEL (https://swissmodel.expasy.org/) using the default parameters (46). A cryo-electron microscopy-determined structure (PDB ID 6xqb, chain A, 3.4-Å resolution) with a high GMQE score was acquired. This model was used to investigate the position of the recombination hot spot in the RdRp of PRRSV-2.

### Construction and rescue of artificially recombinant PRRSVs.

Based on the infectious clones of HuN4 or HeB108, four recombinant plasmids were constructed by switching between complete NSP9 and partial NSP9 (963 bp in the 5′ terminus of NSP9 harboring the recombinant hot spot). Four recombinant plasmids, named pHuN4-HeB108NSP9, pHuN4-HeB108NSP9p, pHeB108-HuN4NSP9, and pHeB108-HuN4NSP9p were validated by DNA sequencing. Marc-145 cells at 80% confluence were seeded in 6-well plates, and 4 μg of each plasmid was transfected into Marc-145 cells using X-treme Gene HP DNA reagent (Roche, Germany) according to the manufacturer’s instructions. At 5 days posttransfection, the viruses were identified by indirect IFA with a monoclonal antibody (3F7) against the M protein of PRRSV-2 (47), according to a previously described method (48). All rescued viruses were passaged three times in Marc-145 cells, harvested, and stored at −80°C. Viral titers were measured with a microtitration assay using Marc-145 cells in 96-well plates and calculated as 50% tissue culture infective dose(s)/mL according to the method of Reed and Muench (49).

### Viral growth kinetics in PAMs or Marc-145 cells.

The growth kinetics of recombinant PRRSVs were compared to those of parental rHuN4 and rHeB108 in PAMs and Marc-145 cells. In brief, the PAMs or Marc-145 cells in 12-well plates were infected with viruses at a multiplicity of infection of 0.01 for 1 h at 37°C. The cells were then washed three times with PBS three times and supplemented with 2% FBS/DMEM, and the viruses were harvested at 0, 24, 48, 72, and 96 hpi. The harvested viruses were titrated in Marc-145 cells (49). Total RNA was extracted, and real-time quantitative PCR was used to detect the copy numbers of PRRSVs as previously described (50).

### Statistical analysis.

All data are expressed as means ± standard deviation (SD). GraphPad Prism v 8.31 was used for statistical analysis, and statistical significance was determined using one-way analysis of variance, followed by Tukey’s *t* test. Statistical significance was set at *P < *0.05.

### Data availability.

The nucleotide sequences of the PRRSVs sequenced in the present study have been deposited in GenBank (accession numbers OM201171 to OM201199).
