# Supplementary material for: Analysis of Recombinant Characteristics Based on 949 PRRSV-2 Genomic Sequences Obtained from 1991 to 2021 Shows That Viral Multiplication Ability Contributes to Dominant Recombination
Source: Microbiol Spectr. 2022 Sep 8;10(5):e02934-22. doi: 10.1128/spectrum.02934-22 (PMC9602502; doi:10.1128/spectrum.02934-22)
Supplement: Supplemental file 1 — Supplemental material. Download spectrum.02934-22-s0001.pdf, PDF file, 1.2 MB [file spectrum.02934-22-s0001.pdf]

Supplementary Figure 1

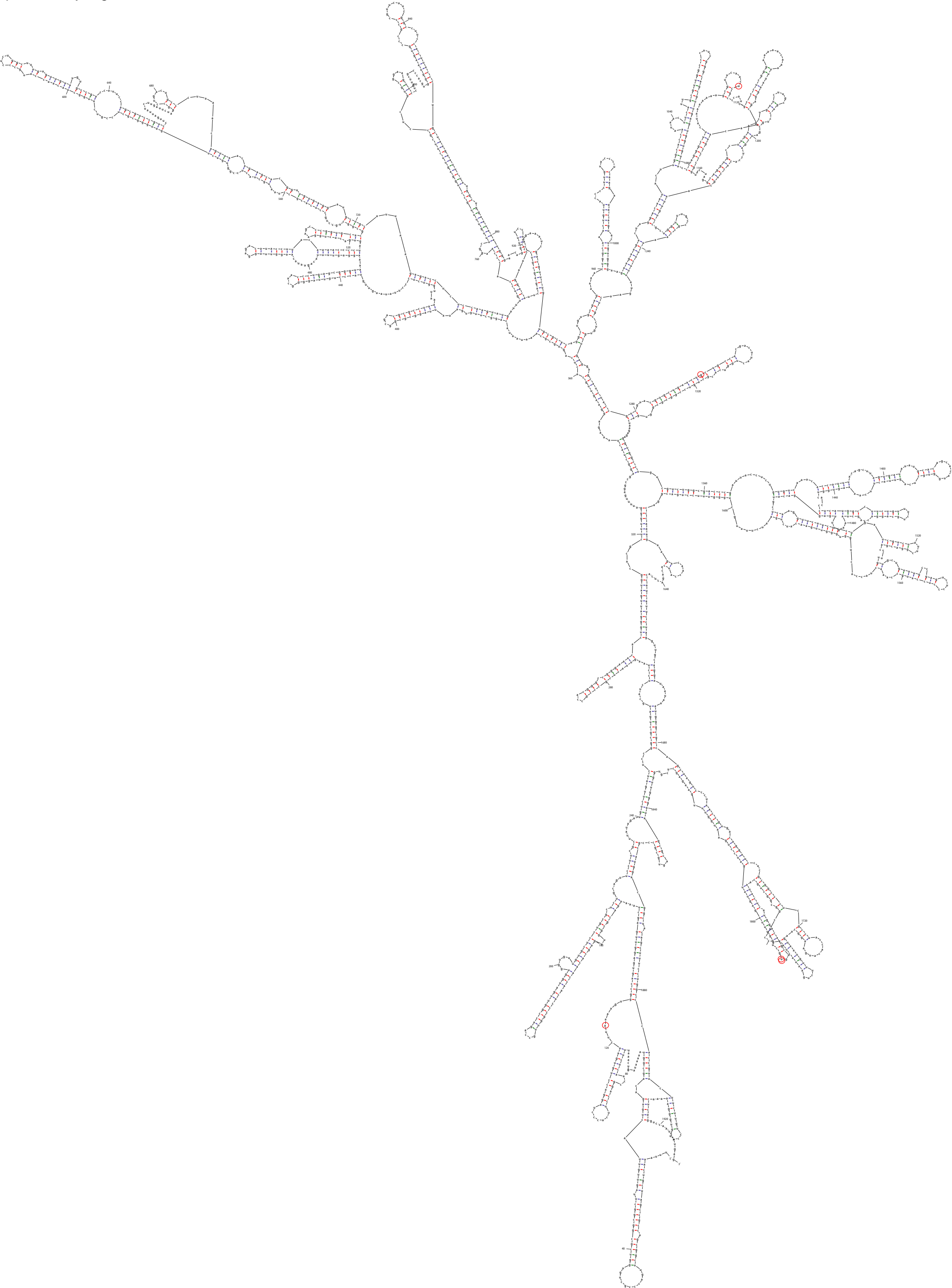

dG = -630.68 [Initially -700.00] JXA1 NSP9

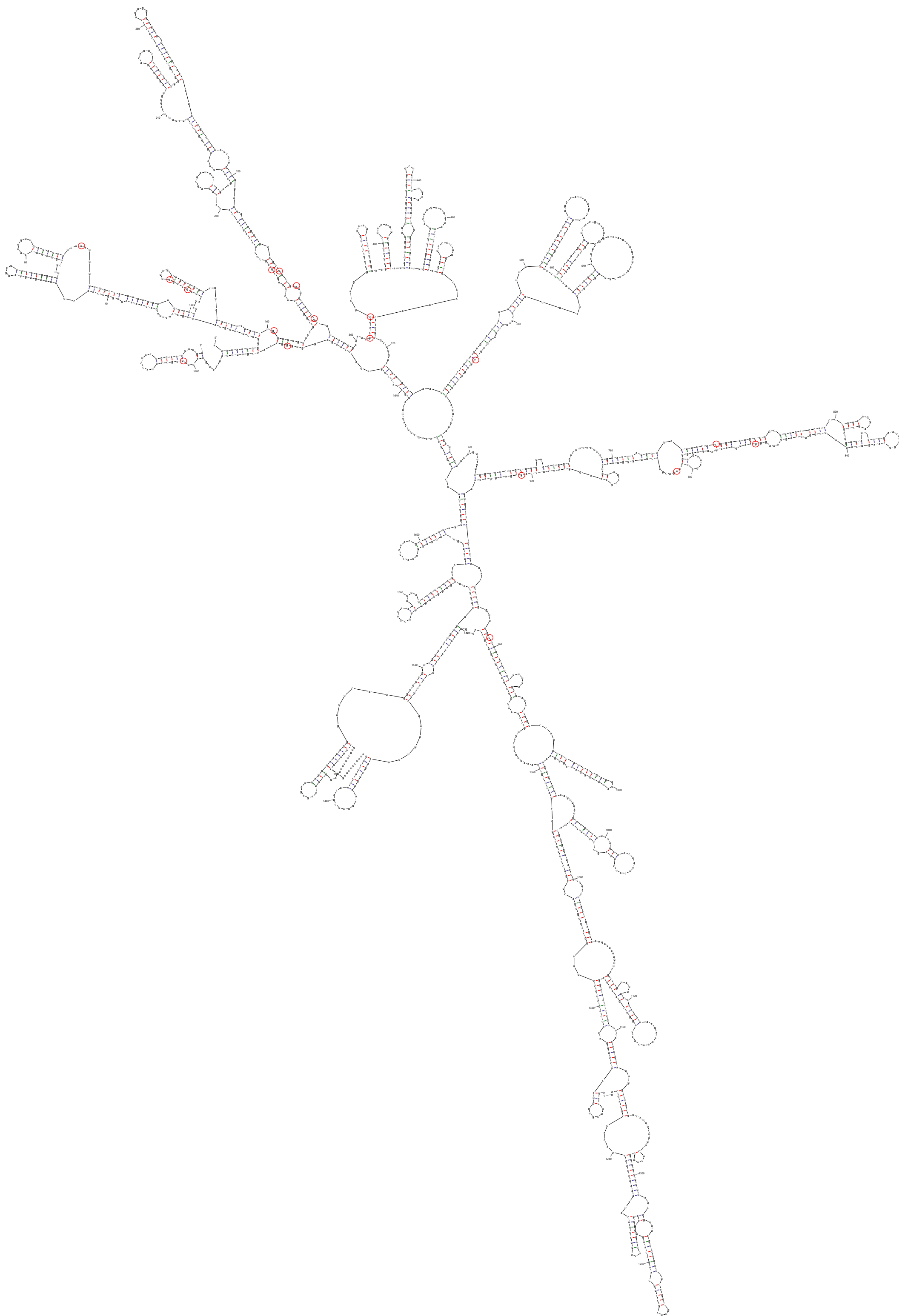

$dG = -516.49$ [Initially -585.20] JXA1 ORF2-ORF4

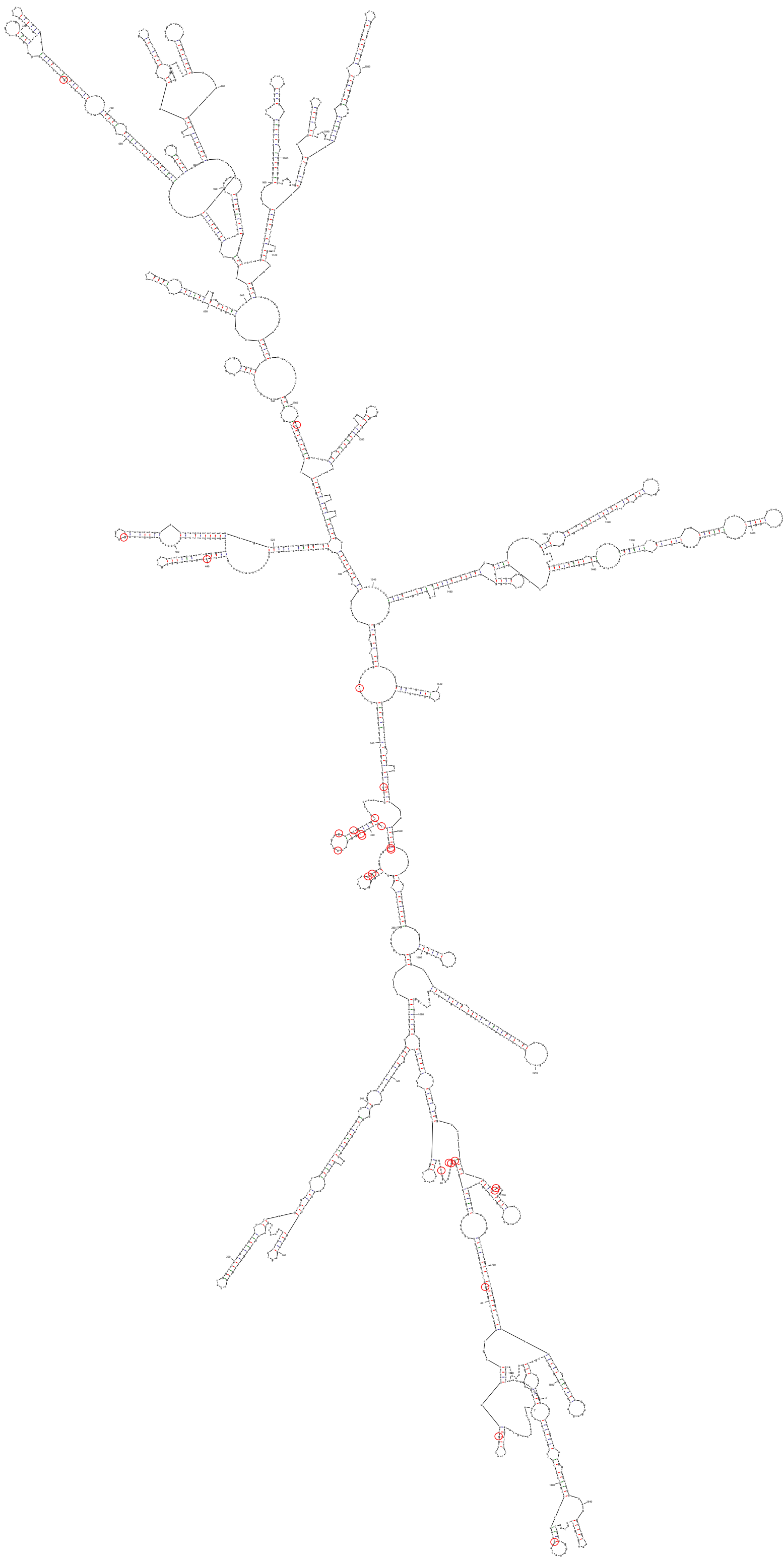

$dG = -629.06$ [Initially -707.50] NADC30 NSP9

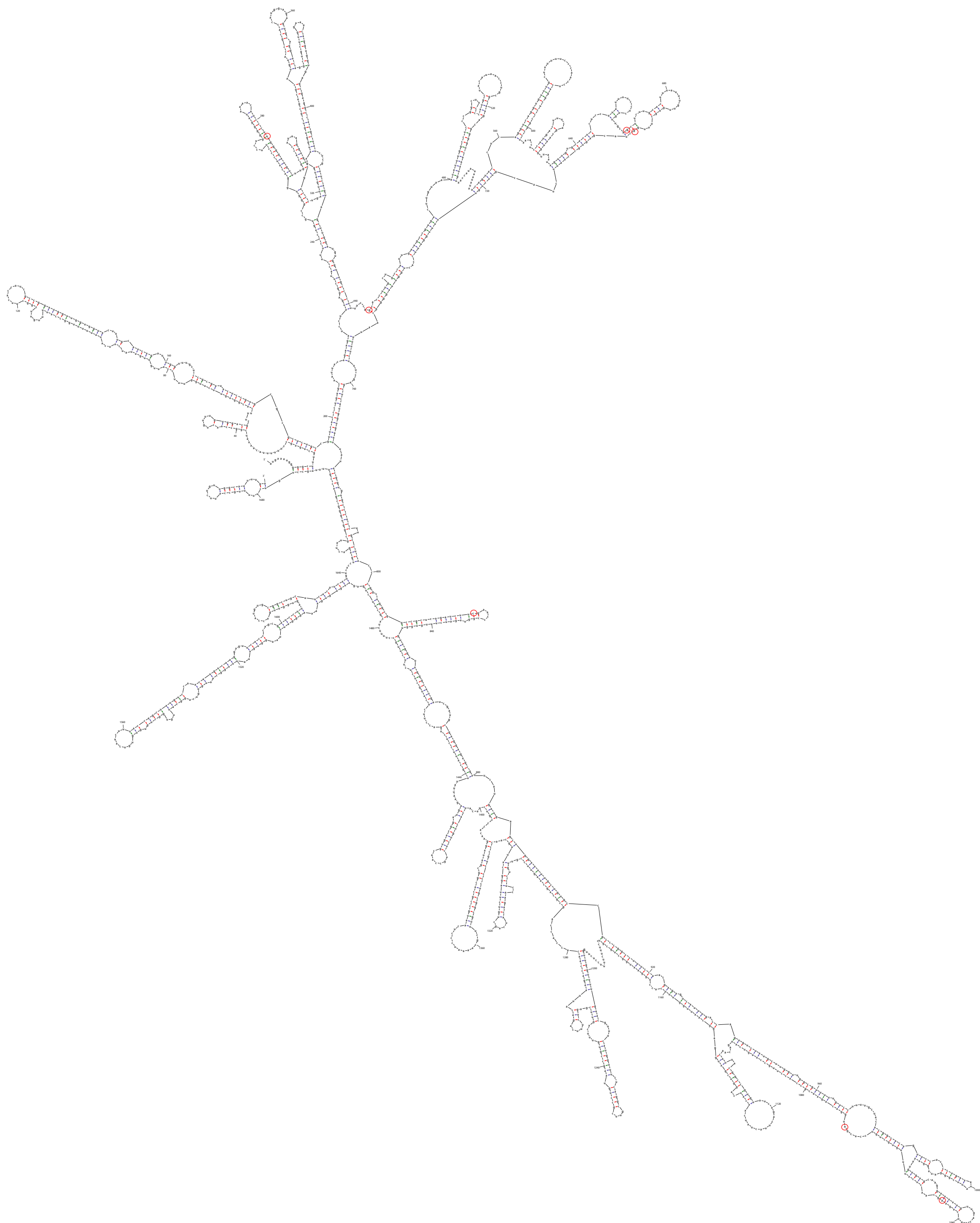

$dG = -543.50$ [Initially -587.00] NADC30 ORF2-ORF4

Fig. S1 RNA secondary structure of the PRRSV NSP9 and ORF2–4 predicted by mfold, recombination hotspots were mapped on the secondary structure and marked by red circles.

Supplementary Figure 2

C101

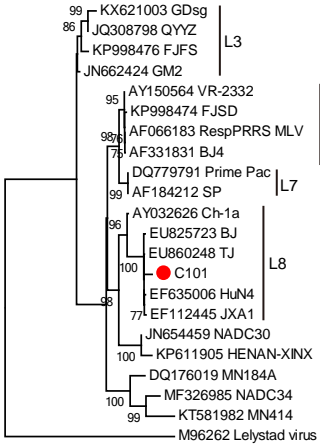

FJDJQ-2018

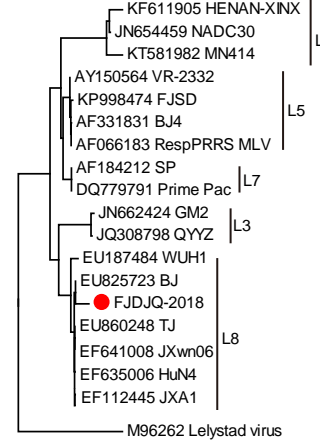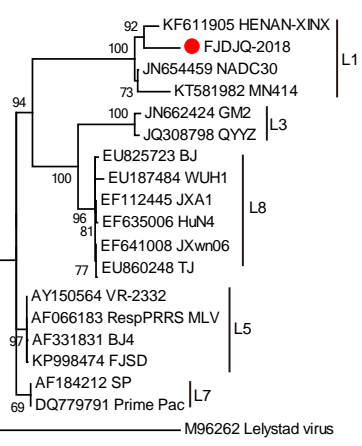

JS1810-195

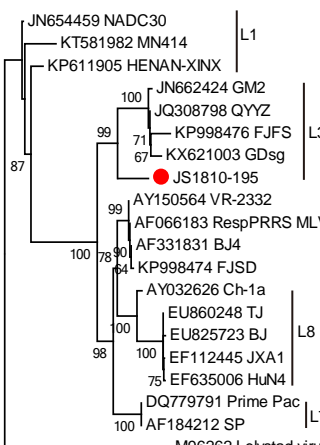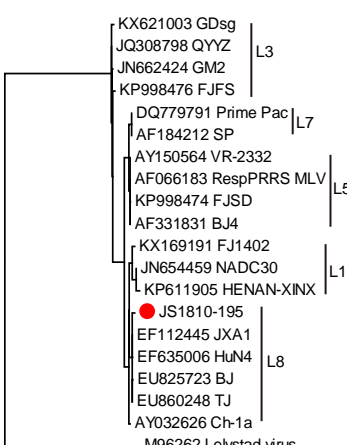

GXNN1396

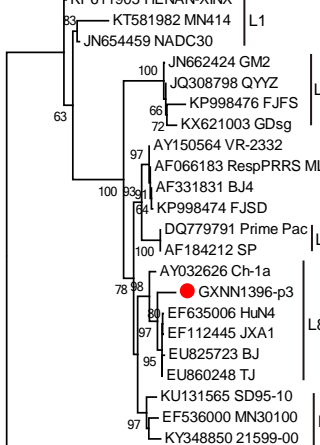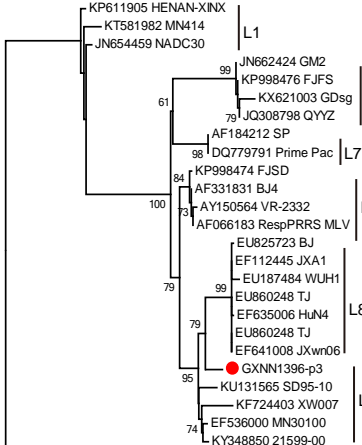

PRRSV2/CN/GDDX/2018

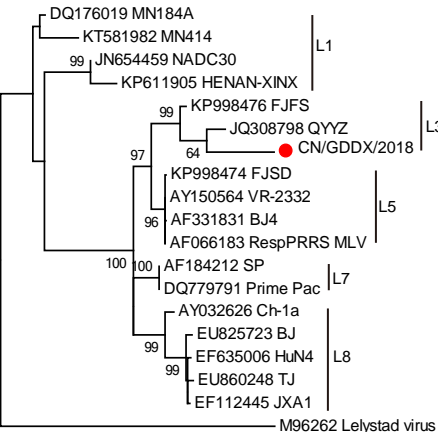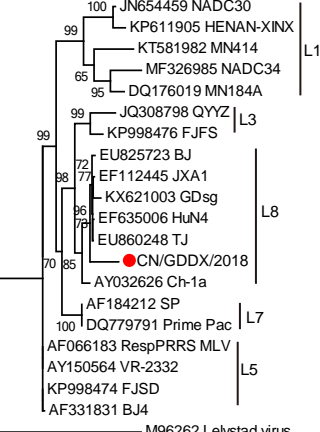

GDsf1808

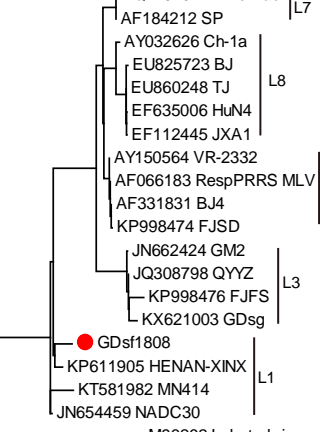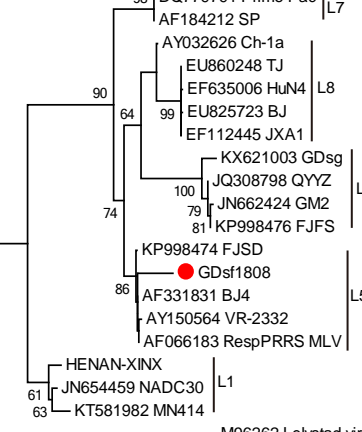

SD43

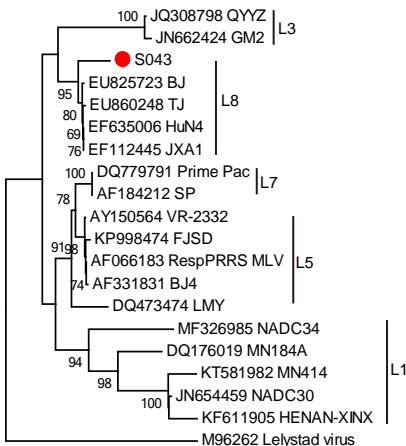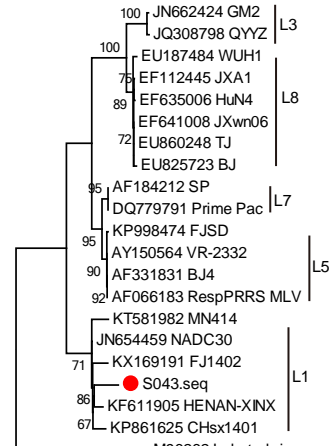

GDHZ_Huizhou

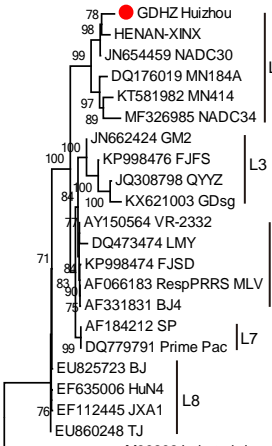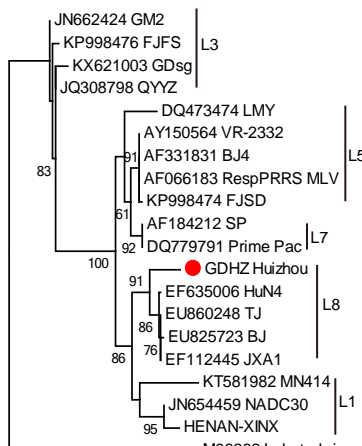

LN86

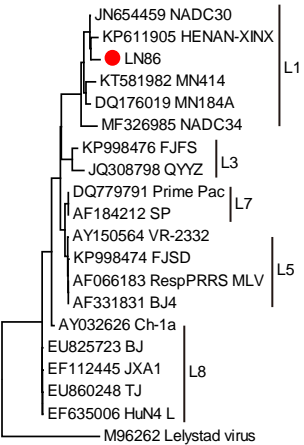

S039

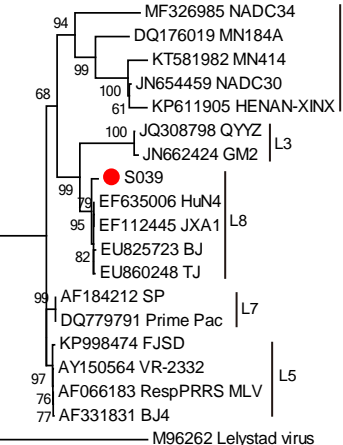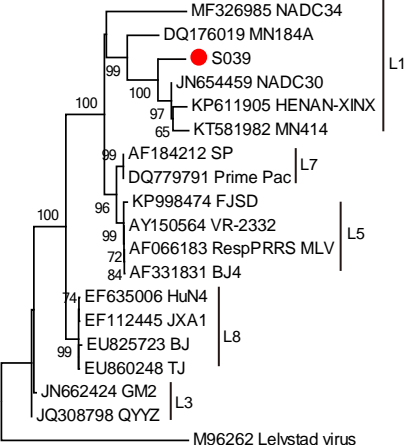

S130

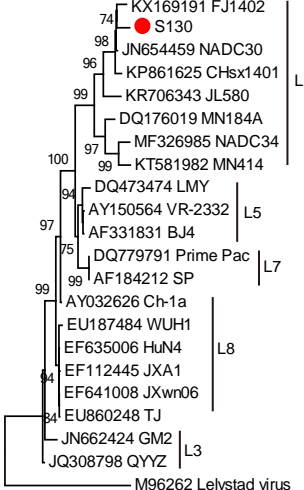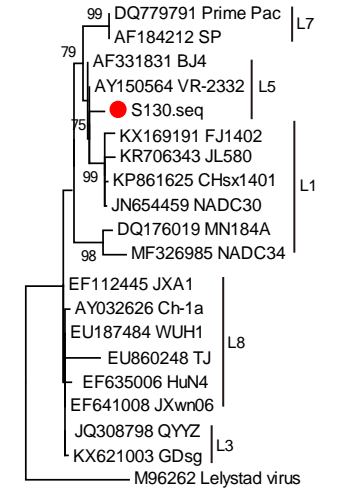

H60

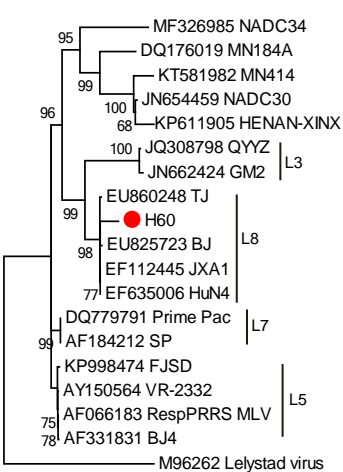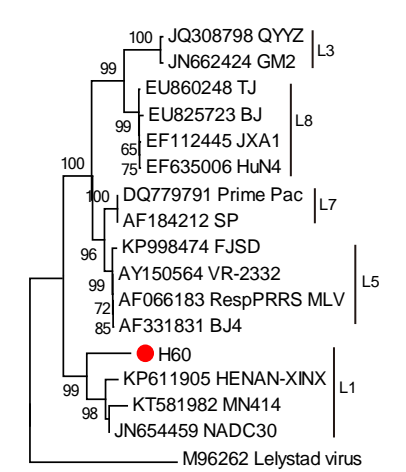

SDbz16-2

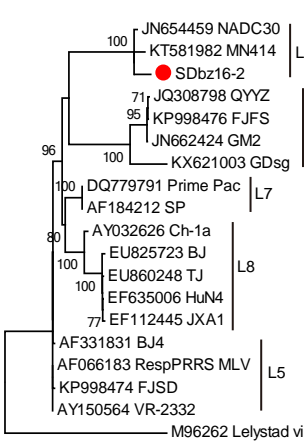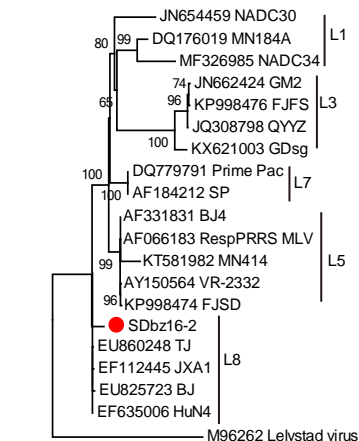

H013

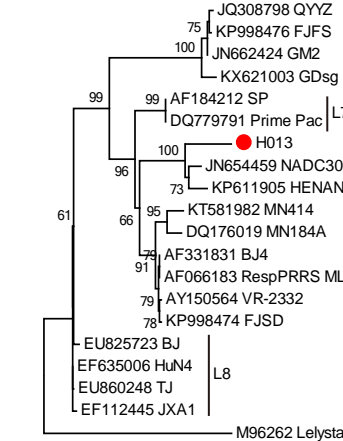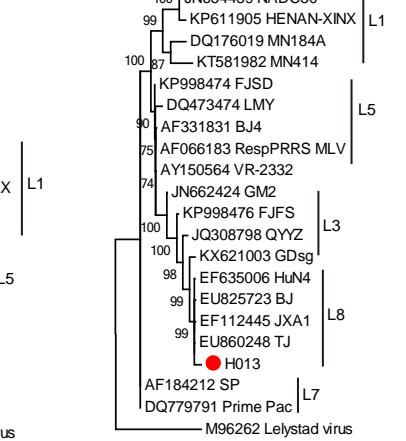

XJ1904-39

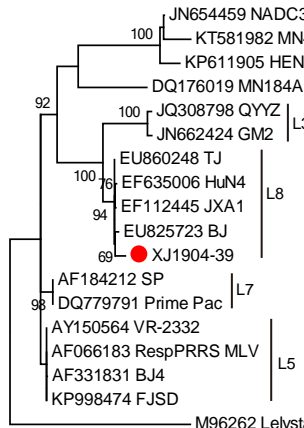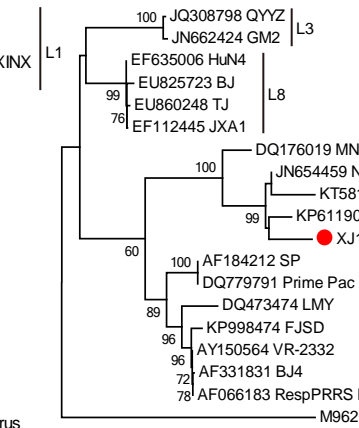

SDqd1501

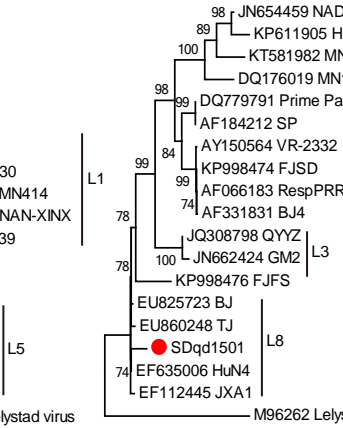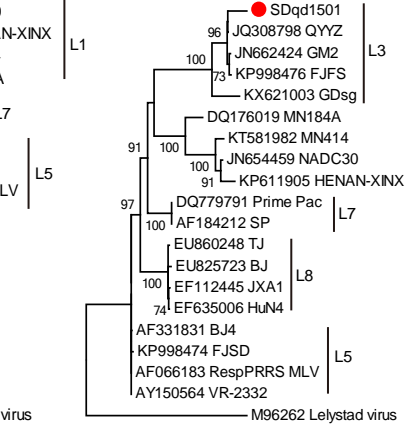

HeN1501

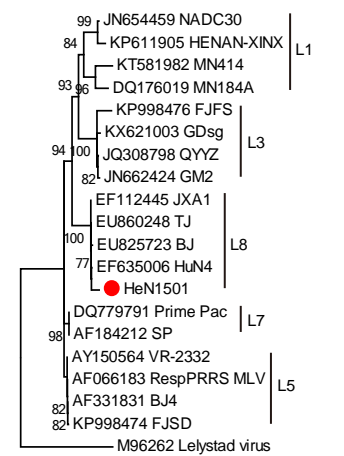

JS18-3

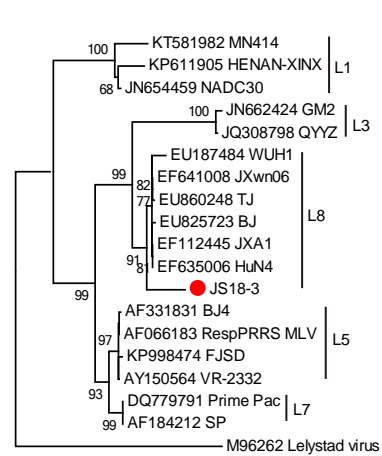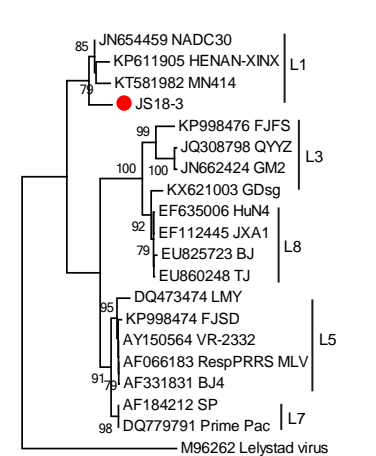

GDsf1809

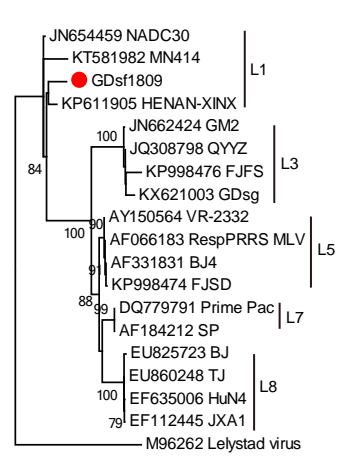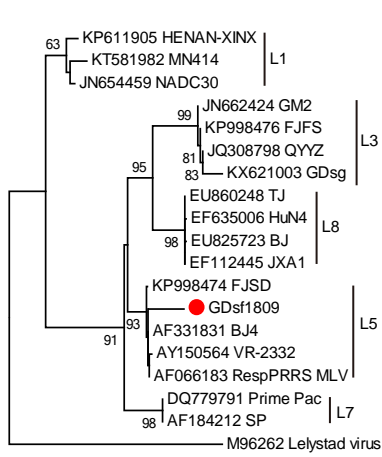

GXNN1839

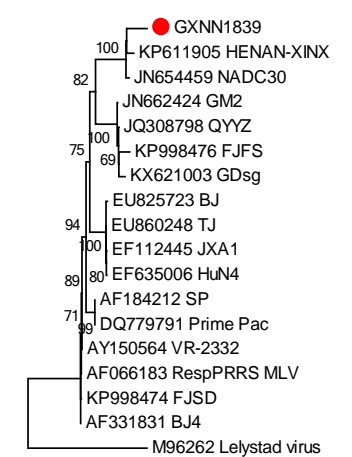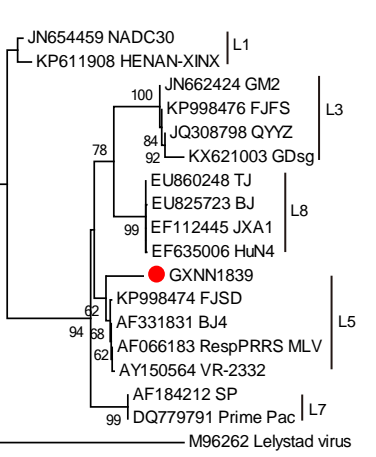

SD110-1608

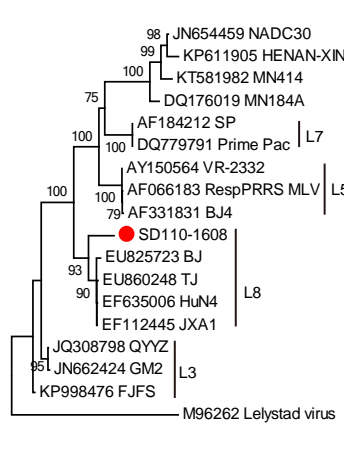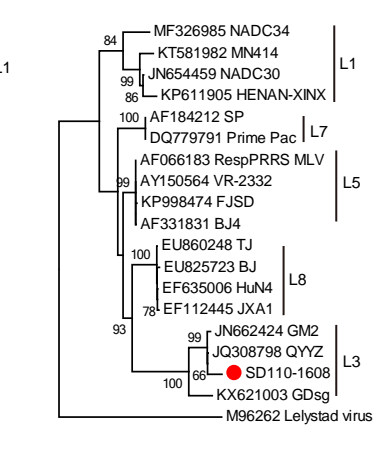

SDwh1601

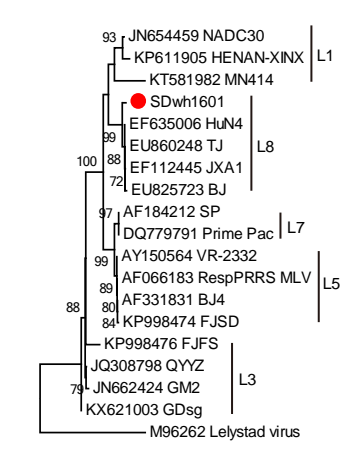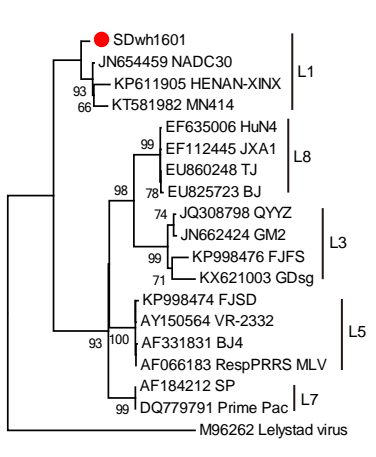

SWU/YB2/2018

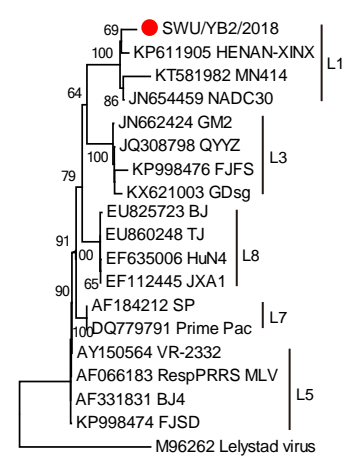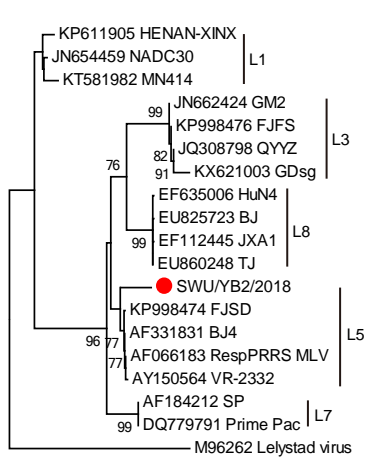

SWU/YB1/22018

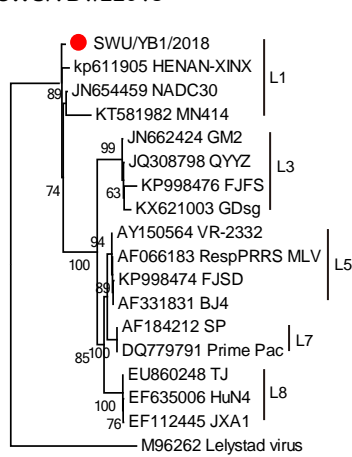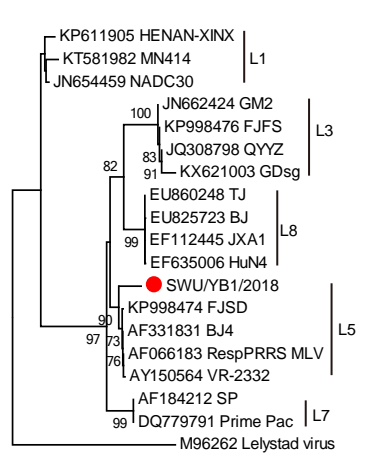

SCya18

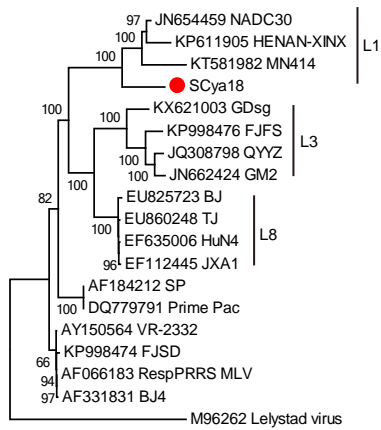

SWU/MS2/2018

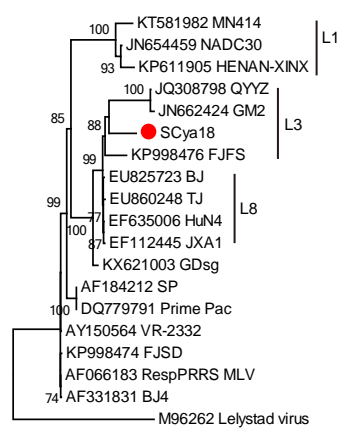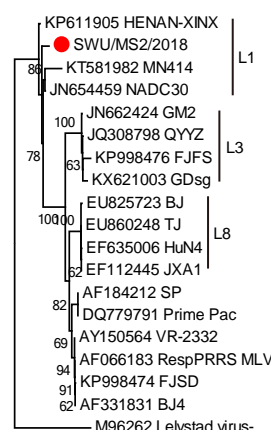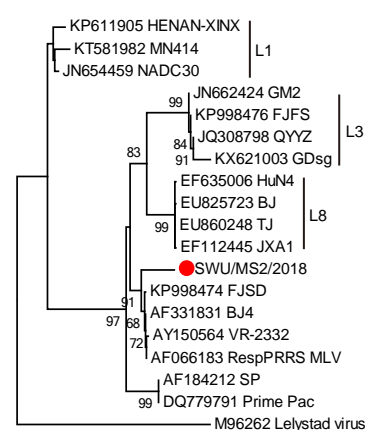

SWU/MS3/2018

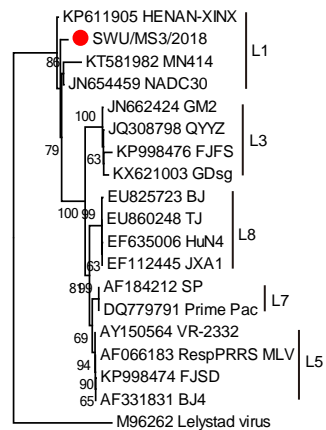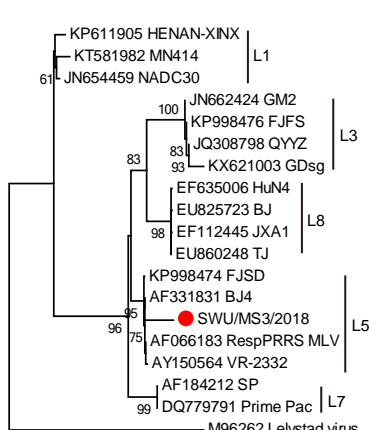

NA80

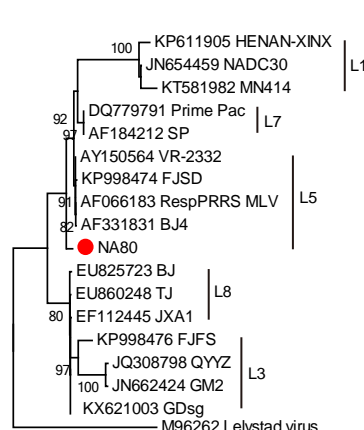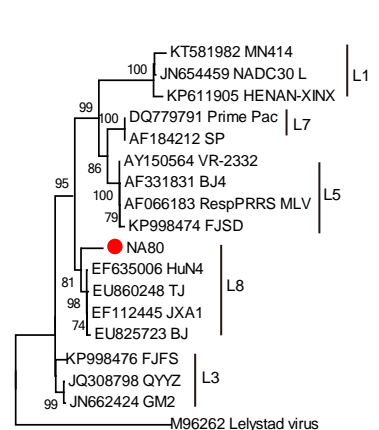

GDQYQC2

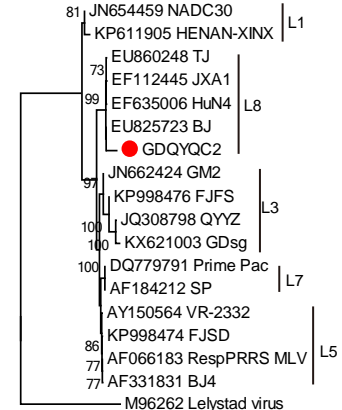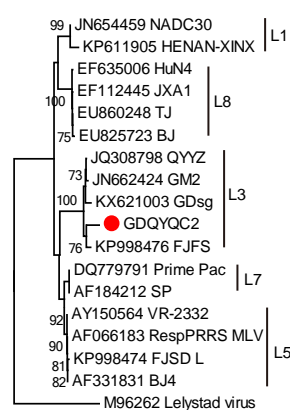

PRRSV/CN/X2984/2018

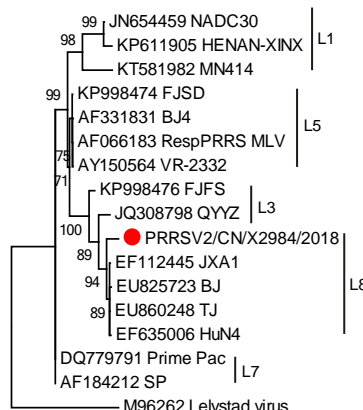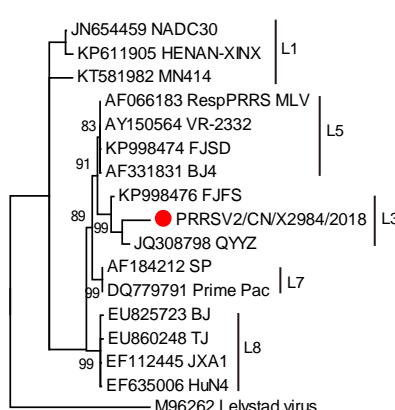

GDsf1806

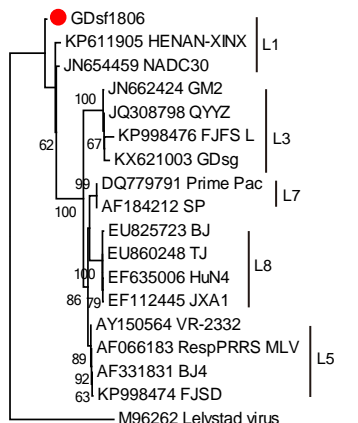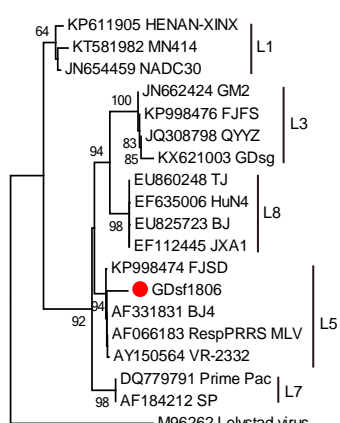

GDsf1710

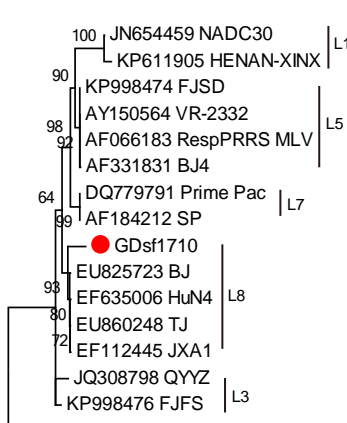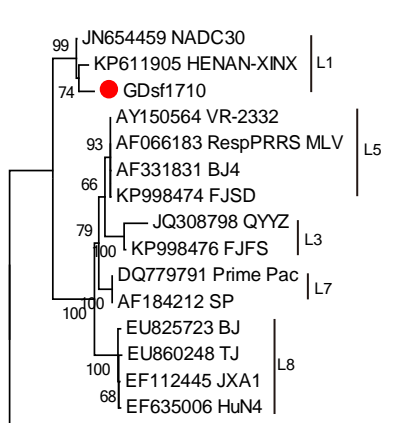

H012

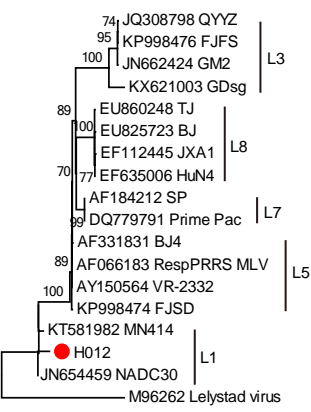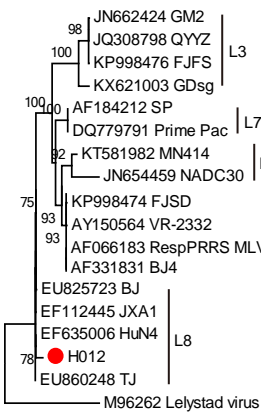

PRRSV2/CN/N9185/2018

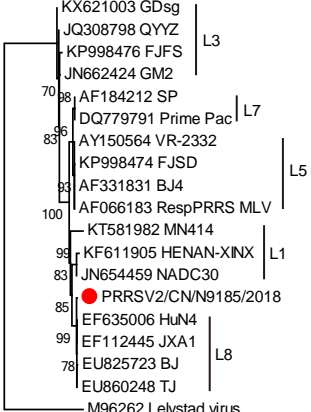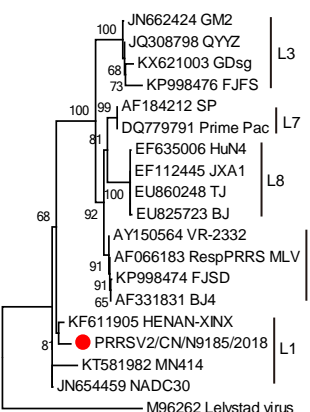

JS2020

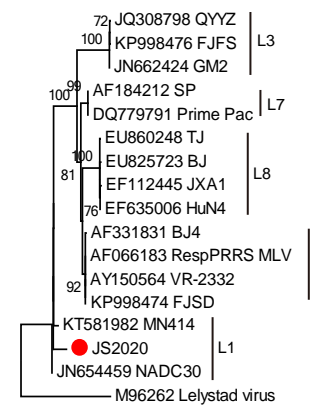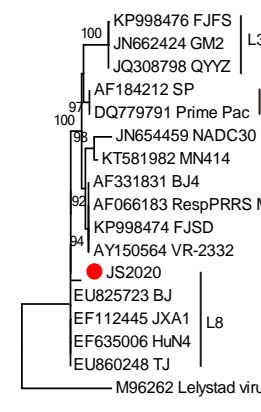

SWU/CD1/2018

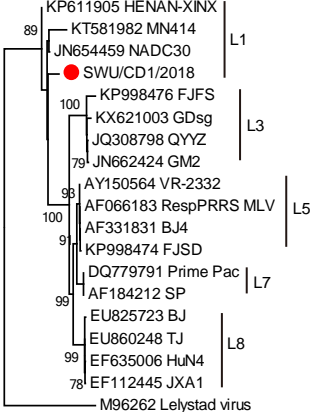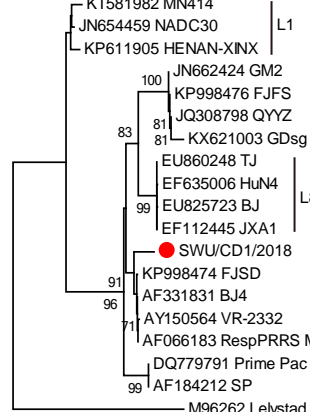

PRRSV2/MY5/2018

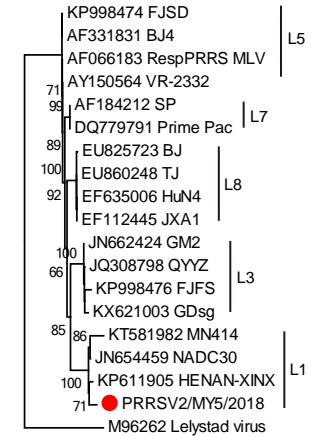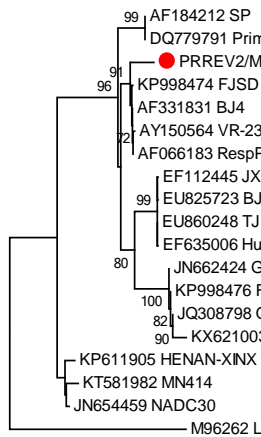

GDsf1807

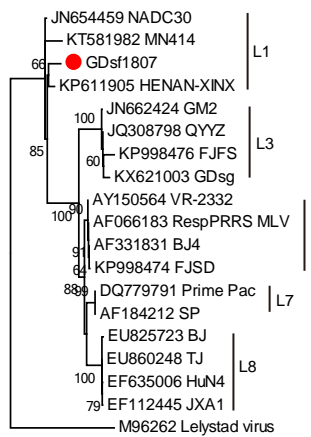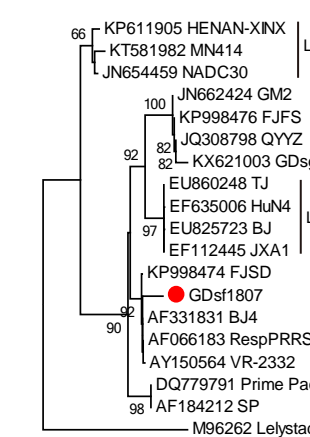

GDsf1804

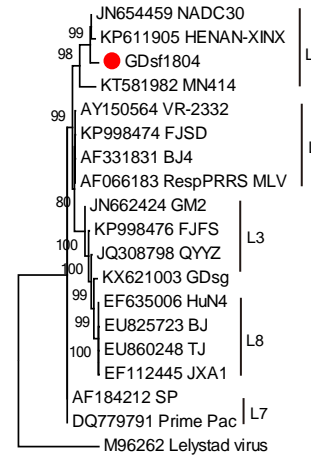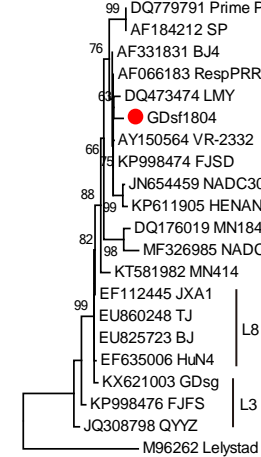

GDsf1711

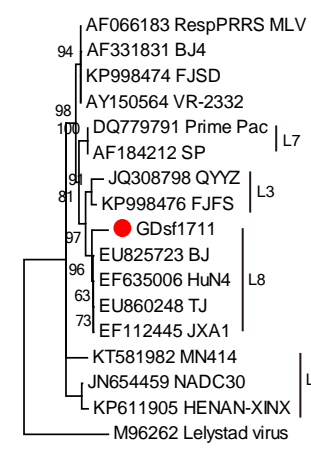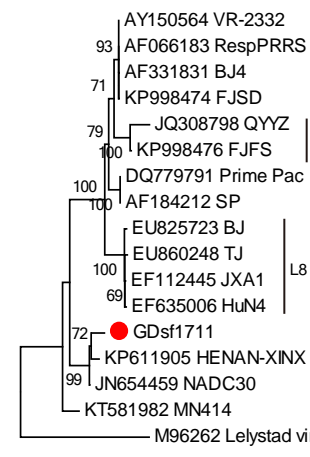

Phylogenetic tree of the VP1 gene of M96262 Lelystad virus and other swine vesicular disease viruses. The tree shows relationships between various virus strains, with bootstrap values indicated at the nodes. A red circle highlights the M96262 Lelystad virus strain. The tree is rooted on the left and branches to the right. The strains are grouped into several clusters labeled L5, L7, L8, L3, and L1.

Strains and their bootstrap values (from top to bottom):

- KP998474 FJSD
- AF331831 BJ4
- AF066183 RespPRRS MLV
- AY150564 VR-2332
- AF184212 SP
- DQ779791 Prime Pac
- EU825723 BJ
- EU860248 TJ
- EF635006 HuN4
- EF112445 JXA1
- JN662244 GM2
- QJ308798 QYYZ
- KP998476 FJFS
- KX621003 GDsg
- KT581982 MN414
- JN654459 NADC30
- KP611905 HENAN-XINX
- SWU/MY6/2018 (highlighted with a red circle)
- M96262 Lelystad virus

Bootstrap values at nodes (from top to bottom): 73, 100, 90, 100, 93, 62, 68, 100, 65, 85, 89, 100, 76.

Clusters labeled on the right:

- L5 (top cluster)
- L7 (cluster containing DQ779791 Prime Pac)
- L8 (cluster containing EU825723 BJ, EU860248 TJ, EF635006 HuN4, EF112445 JXA1)
- L3 (cluster containing KP998476 FJFS, KX621003 GDsg)
- L1 (bottom cluster)

Phylogenetic tree of the VP1 gene of M96262 Lelystad virus and other related viruses. The tree shows the evolutionary relationships between various virus strains. The strains are listed on the left, and their corresponding bootstrap values are shown on the right. The strains are: JN654459 NADC30, KP611905 HENAN-XINX, KT581982 MN414, AF331831 BJ4, KP998474 FJSD, AF066183 RespRRS MLV, AY150564 VR-2332, DQ779791 Prime Pac, AF184212 SP, JQ308798 QYYZ, KP998476 FJFS, GDSf1802 (marked with a red dot), EU825723 BJ, EF635006 HuN4, EU860248 TJ, EF112445 JXA1, and M96262 Lelystad virus. The tree is rooted at the bottom with M96262 Lelystad virus. The branches are labeled with bootstrap values: 95, 99, 97, 92, 80, 98, 94, 78, and 74. The tree is divided into several clades, with some clades labeled L1, L5, L7, L3, and L8.

Sequence list (from top to bottom):

- JN654459 NADC30
- KP611905 HENAN-XINX
- KT581982 MN414
- AF331831 BJ4
- KP998474 FJSD
- AF066183 RespRRS MLV
- AY150564 VR-2332
- DQ779791 Prime Pac
- AF184212 SP
- JQ308798 QYYZ
- KP998476 FJFS
- GDSf1802 (marked with a red dot)
- EU825723 BJ
- EF635006 HuN4
- EU860248 TJ
- EF112445 JXA1
- M96262 Lelystad virus

Bootstrap values (from top to bottom):

- 95
- 99
- 97
- 92
- 80
- 98
- 94
- 78
- 74

Clade labels (from top to bottom):

- L1
- L5
- L7
- L3
- L8

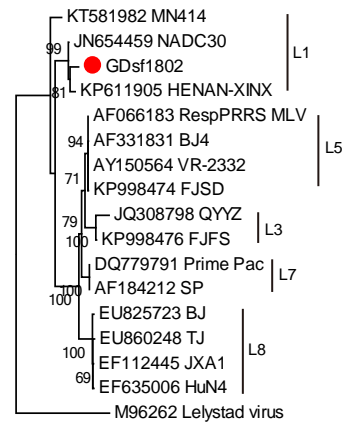

Phylogenetic tree of the VP1 gene of HsPV1 and other polyomaviruses. The tree shows HsPV1 (red dot) as a novel polyomavirus distinct from known ones. Bootstrap values are indicated at the nodes. Scale bar = 0.01 substitutions per site.

Sequences (from top to bottom):

- KT581982 MN414
- JN654459 NADC30
- KP611905 HENAN-XINX
- AF066183 RespPRRS MLV
- AF331831 BJ4
- KP998474 FJSD
- AY150564 VR-2332
- DQ779791 Prime Pac
- AF184212 SP
- GDS1f707
- QJ308798 QYYZ
- KP998476 FJFS
- EU825723 BJ
- EF635006 HuN4
- EU860248 TJ
- EF112445 JXA1
- M96262 Lelystad virus

Bootstrap values (from top to bottom):

- 99
- 96
- 95
- 97
- 91
- 83
- 97
- 96
- 66

Scale bar = 0.01 substitutions per site.

Phylogenetic tree showing the relationships between various PRRSV2 strains. The tree is rooted at the bottom left. The strains are listed on the right, with their corresponding bootstrap values indicated by numbers at the nodes. The strains are grouped into clades L1, L3, L5, and L8. A red circle highlights the PRRSV2/CN/F1228/2017 strain, which is closely related to M62622 Levstad virus.

Strains and their bootstrap values (from top to bottom):

- JN654459 NADC30 (100)
- KP611905 HENAN-XINX (95)
- QF176019 MN184A (95)
- AF184212 SP (99)
- DQ779791 Prime Pac (99)
- AY150564 VR-2332 (85)
- AF331831 BJ4 (92)
- KP998474 FJSD (98)
- AF066183 RespPRRS MLV (98)
- QJ308798 QYYZ (100)
- JN662424 GM2 (100)
- EU825723 BJ (97)
- EF112445 JXA1 (97)
- EF635006 HuN4 (97)
- EU860248 TJ (97)
- PRRSV2/CN/F1228/2017 (red circle)
- M62622 Levstad virus (97)

Clades and their bootstrap values (from top to bottom):

- L1 (100)
- L7 (99)
- L5 (98)
- L3 (100)
- L8 (97)

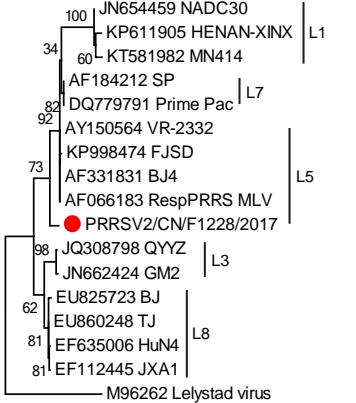

Phylogenetic tree of the VP1 gene of SDy1401 and other swine vesicular disease virus strains. The tree shows SDy1401 (red circle) as a novel strain closely related to M96262 Lelystad virus. Other strains are grouped into clusters L1, L3, L5, L7, and L8. Bootstrap values are indicated at the nodes.

- 98 JN654459 NADCC30
- 86 KP611905 HENAN-XINX | L1
- KT581982 MM414
- KP998476 FJFS
- 100 KX621003 GDsg | L3
- JQ308798 QYYZ
- 81 JN662424 GM2
- 96 AF184212 SP
- 96 DQ779791 Prime Pac | L7
- AY150564 VR-2332
- 89 AF066183 RespPRRS MLV | L5
- 81 KP998474 FJSD
- 83 AF331831 BJ4
- EF112445 JXA1
- 100 EU825723 BJ | L8
- EU860248 TJ
- 78 EF635006 HuN4 L
- 79 ● SDy1401
- M96262 Lelystad virus

Phylogenetic tree of the VP1 gene of the GDHh1808 virus. The tree shows GDHh1808 (red dot) as a novel virus, closely related to EF635006 HuN4 and EF112445 JXA1. Other sequences include JQ308798 QYYZ, JN662424 GM2, EU860248 TJ, EU825723 BJ, JN654459 NADC30, KT581982 MN414, KP611905 HENAN-XIN, DQ779791 Prime Pac, AF184212 SP, KP998474 FJSD, AY150564 VR-2332, AF066183 RespPRRS MLV, AF331831 BJ4, and M92626 Lelystad virus. Bootstrap values are shown at the nodes.

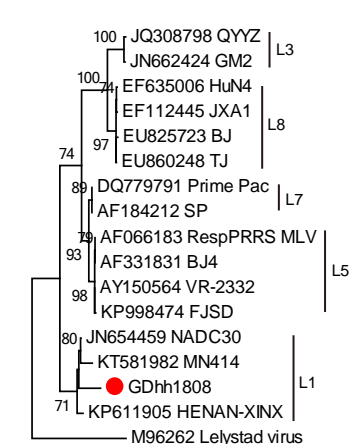

Phylogenetic tree showing the relationship between PRRSV2/CN/X9830/2018 (highlighted with a red circle) and other PRRSV2 strains. The tree is rooted at the bottom with M96262 Lelystad virus. The tree is divided into several clades labeled L1, L3, L5, L7, and L8.

- 97 83 PRRSV2/CN/X9830/2018
- 83 KP611905 HENAN-XINX
- JN654459 NADC30
- KT581982 MN414
- JN662424 GM2
- KP998476 FJS
- KX621003 GDsg
- 100 JQ308798 QYZ
- 94 AY150564 VR-2332
- 94 AF066183 RespPRRS MLV
- 94 KP998474 FJSD
- 94 AF331831 BJ4
- 80 AF184212 SP
- 100 DQ779791 Prime Pac
- 100 EU825723 BJ
- 100 EU860248 TJ
- 100 EF635006 HuN4
- EF112445 JXA1
- M96262 Lelystad virus

Phylogenetic tree of PRRSV strains based on the GP5 gene. The tree shows relationships between various strains, with a red circle highlighting the PRRSV/CN/X4836/2019 strain. Bootstrap values are indicated at the nodes. The tree is rooted with EU825723 BJ and branches into several groups, including L7, L3, and L8.

- 65 PRRSV/CN/X4836/2019 (highlighted with a red circle)
- 100 KP611905 HENAN-XINX
- JN654459 NADC30
- 89 DQ779791 Prime Pac
- 95 AF184212 SP
- 89 AY150564 VR-2332
- 99 AF331831 BJ4
- 78 KP998474 FJSD
- 80 AF066183 RespPRRS MLV
- 87 KP998476 FJFS
- 88 JQ308798 QYYZ
- EU860248 TJ
- EF635006 HuN4
- EF112445 JXA1
- EU825723 BJ
- M96262 Lelystad virus

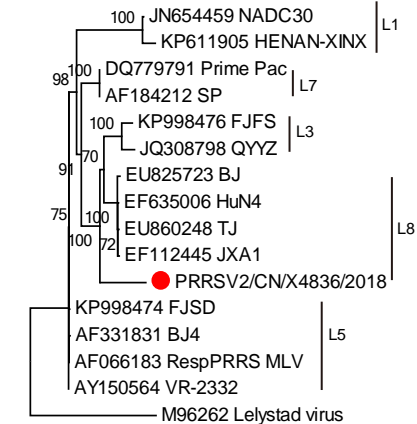

GDSc1809

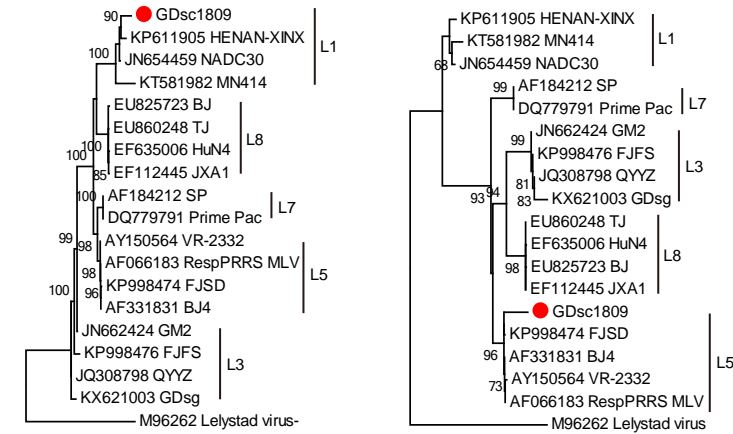

PRRSV2/CN/X4833/2018

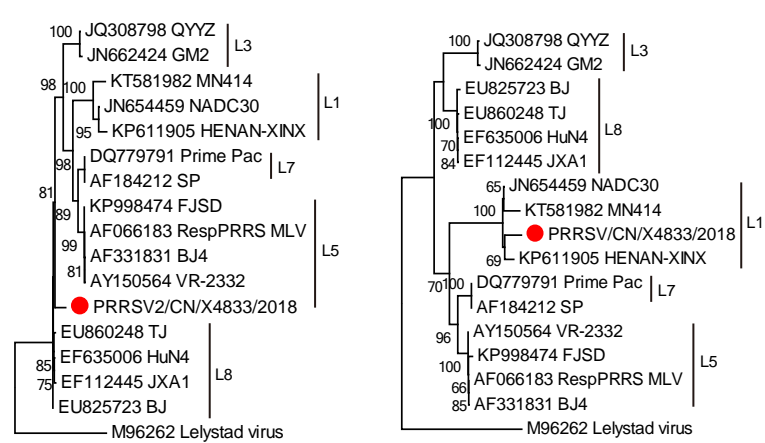

GXNN202004a

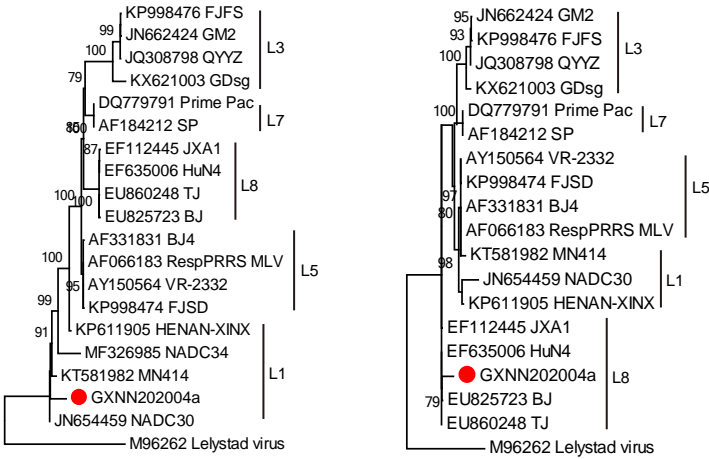

GXNN202004

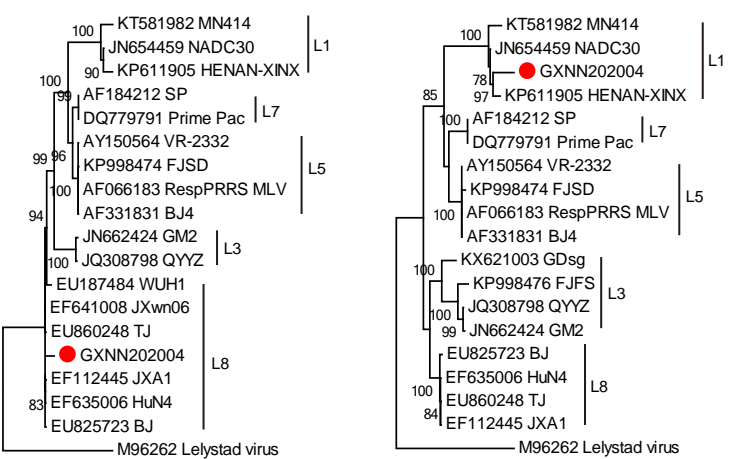

PRRSV2/CN/X4839/2017

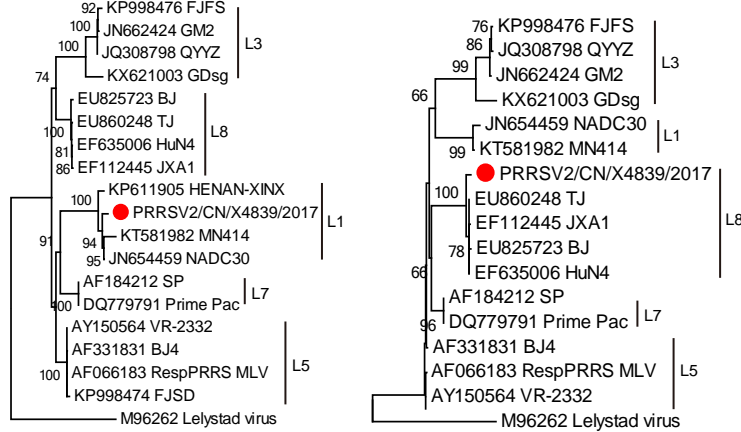

SD-YL1712

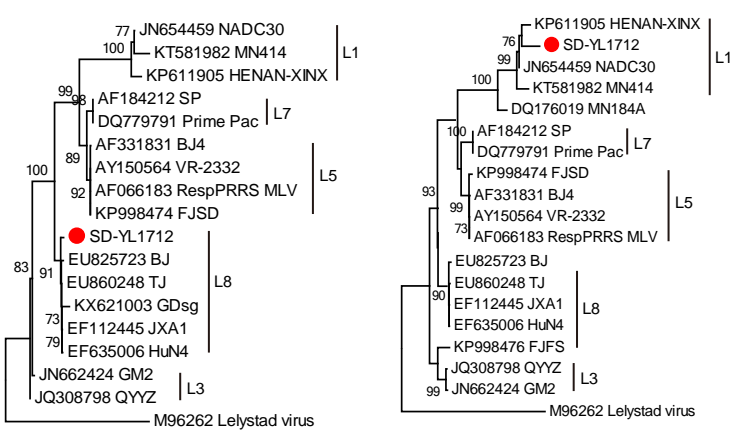

PRRSV2/CN/X4831/2018

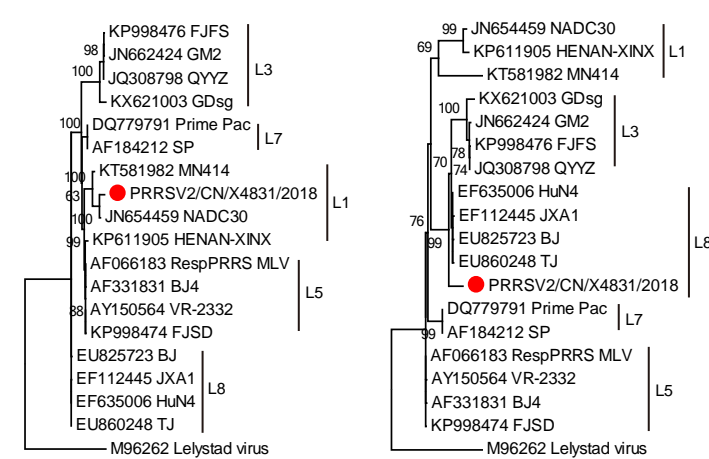

GD1909

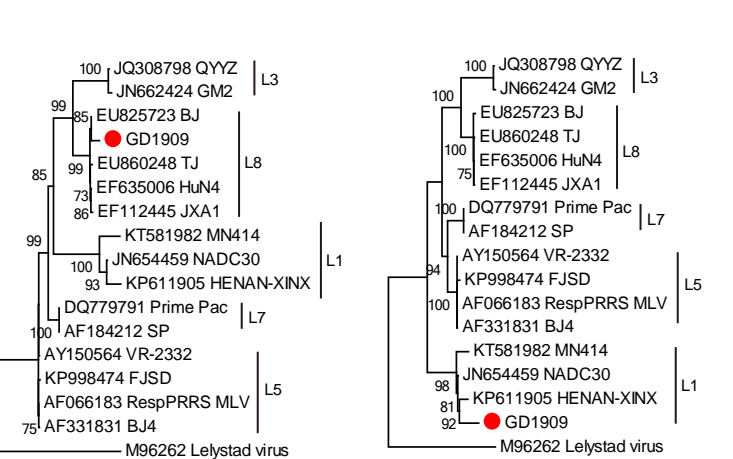

SD99-1606

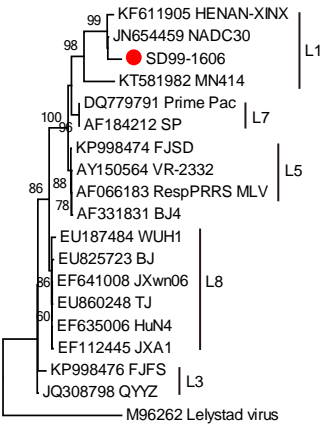

HBap4/2018

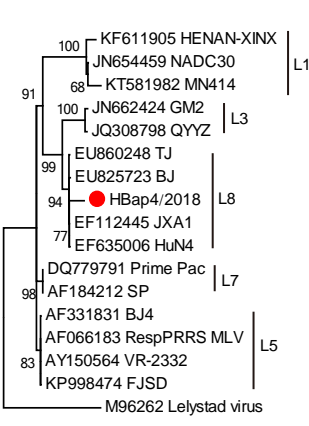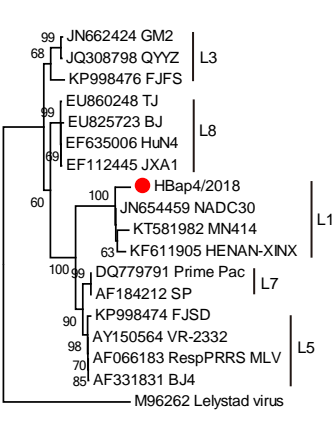

SD1704-23

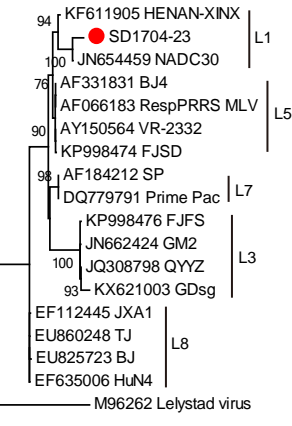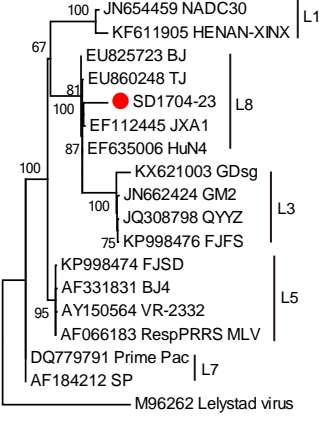

S136

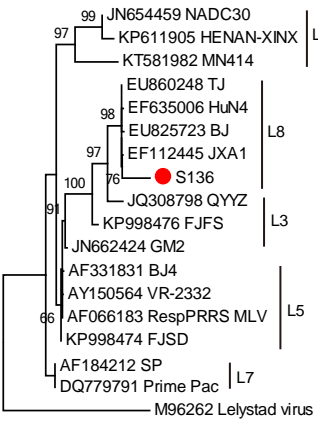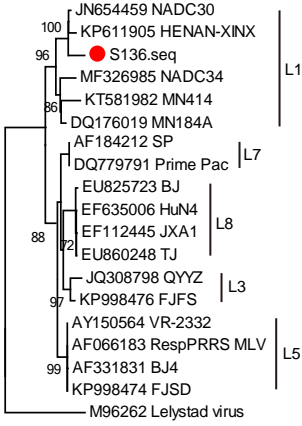

HN1804-2

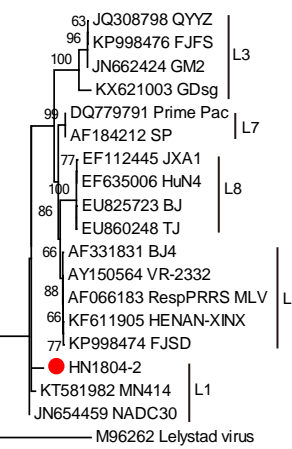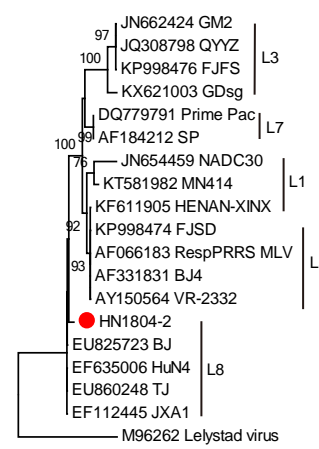

SD/DJY

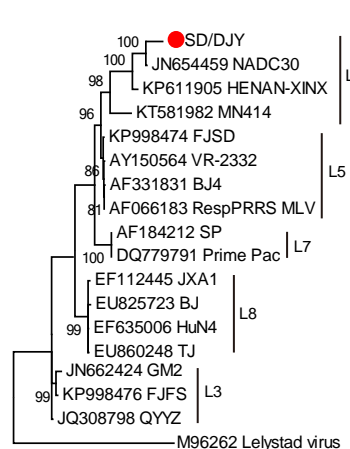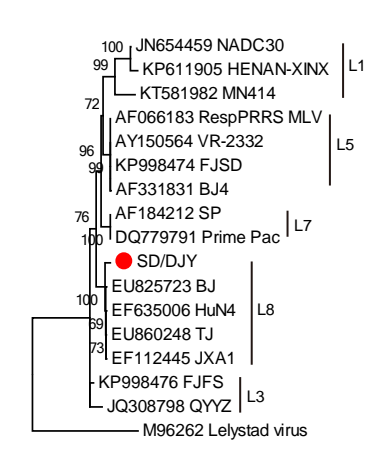

SCcd2020

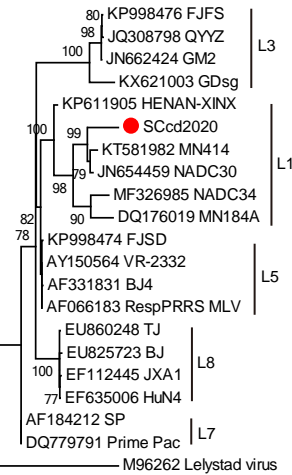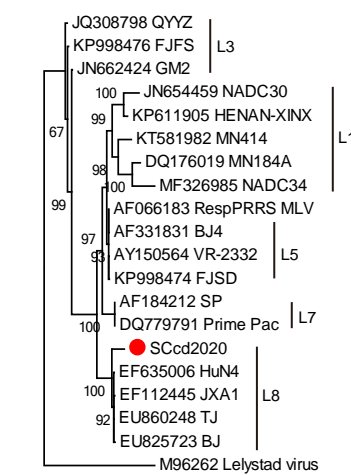

GDSc1808

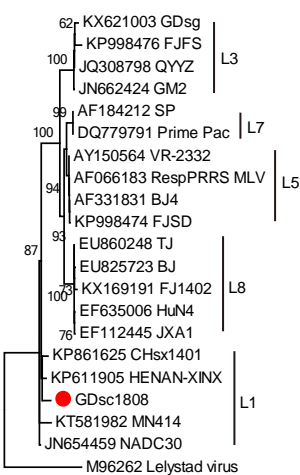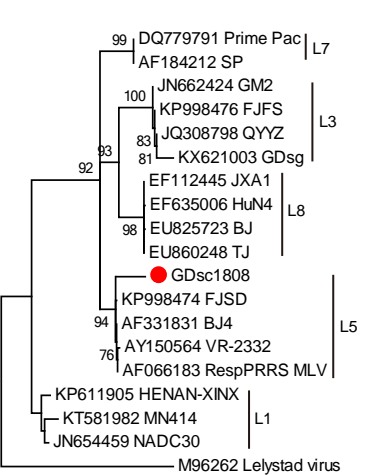

SDwh1403

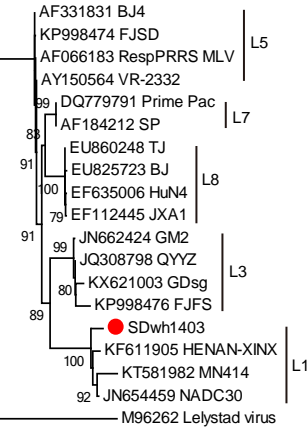

HZ1-3_Yunnan_20171

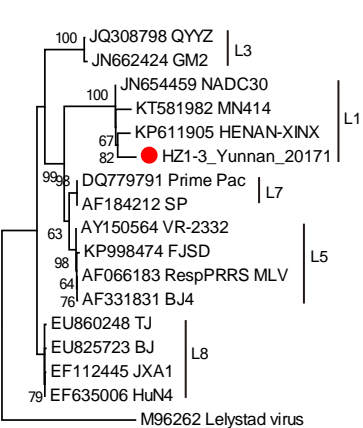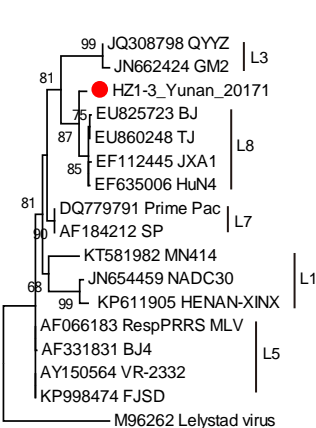

FS-GD-02

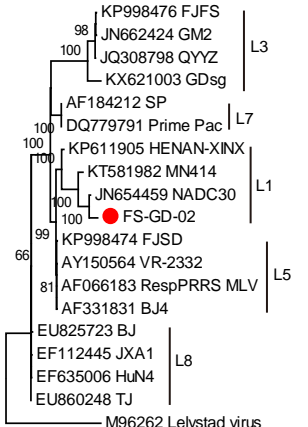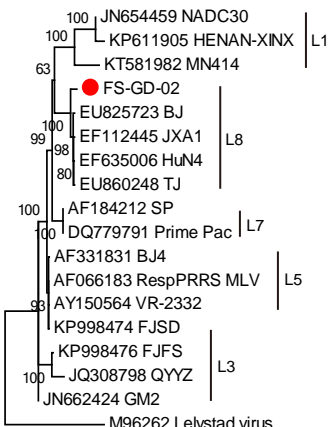

S145

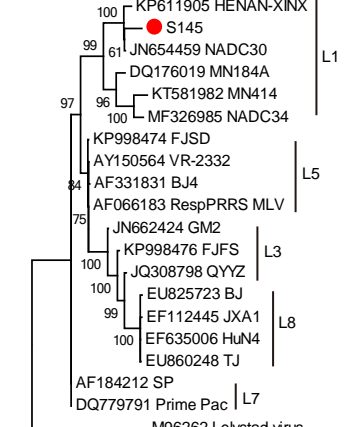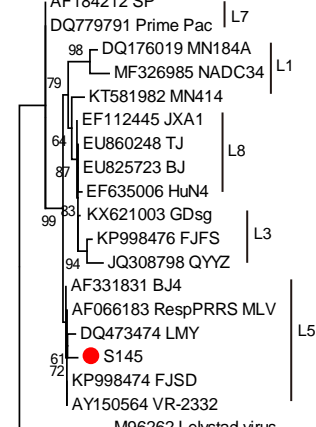

S001

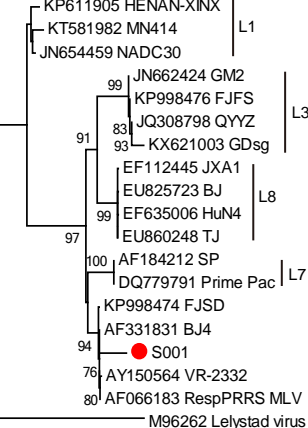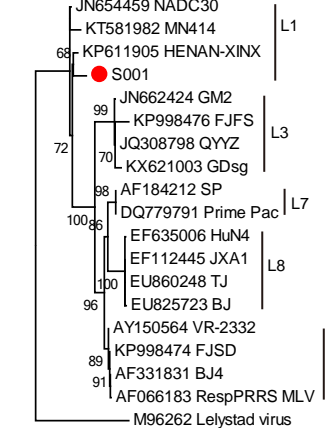

PRRSV2/CN/110713/2018

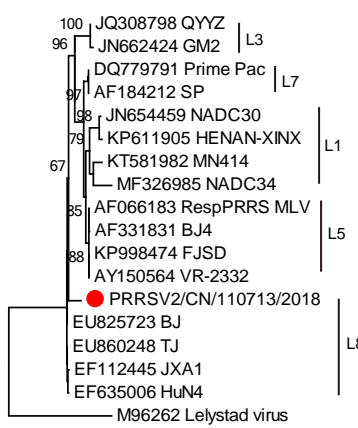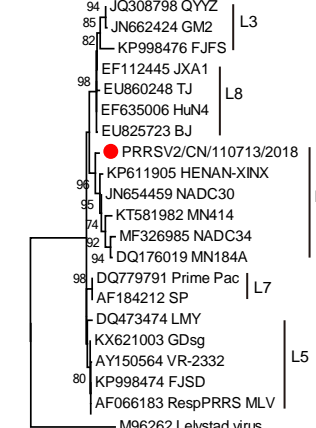

Fig. S2 Phylogenetic analysis based on the recombinant region of recombinant strains.

Supplementary Table 1. Strains information used in this study.

Accession number	Strain	Date	Country	Province/<br>States	Lineage
AF176348	PA8	na	Canada	Alberta	L5
	HB94-lun	2019	China	Hebei	L1
	S145	2019	China	Shandong	L1
	S130-lym	2019	China	Shandong	L1
	G52	2018	China	Guangdong	L8
	G101-lun	2019	China	Guangdong	L5
	S039	2018	China	Shandong	L1
	S75	2018	China	Shandong	L8
	S022-lun	2018	China	Shandong	L8
	H60-lun	2018	China	Hebei	L1
	NA80-lun	2018	China	Heilongjiang	L5
	HB96-lun	2019	China	Hebei	L5
	LN86-lun	2018	China	Liaoning	L1
	G122-lun	2018	China	Guangdong	L8
	S032-lun	2018	China	Shandong	L8
	S78-lun	2018	China	Shandong	L1
	H013	2018	China	Hebei	L1
	S77	2018	China	Shandong	L1
	S020	2018	China	Shandong	L1
	S70	2018	China	Shandong	L3
	G59-lun	2018	China	Guangdong	L3
	G128-lun	2018	China	Guangdong	L3
	C103-lun	2019	China	Chongqing	L5
	G113-lun	2018	China	Guangdong	L3
	S043	2018	China	Shandong	L1
	S136-lun	2019	China	Shandong	L1
	H012	2018	China	Hebei	L1
	S001-lun	2018	China	Shandong	L1
	H64	2019	China	Heilongjiang	L1
	ZJ83-lun	2018	China	Zhejiang	L3
AF331831	BJ-4	1997	China	Beijing	L5
AY032626	CH-1a	1996	China	Beijing	L8
AY150312	HB-1(sh)/2002	2002	China	Hebei	L8
AY262352	HB-2(sh)/2002	2002	China	Hebei	L8
AY457635	HN1	na	China	Hunan	L5
DQ459471	S1	na	China	na	L5
EF075945	HUB1	2006	China	Hubei	L8
EF112445	JXA1	2006	China	Jiangxi	L8
EF112446	HUB2	2006	China	Hubei	L8
EF112447	HEB1	2006	China	Hebei	L8

EF153486	CC-1	2005	China	Jilin	L5
EF517962	HuN	na	China	Hunan	L8
EF635006	HUN4	2006	China	Hunan	L8
EF641008	JXwn06	2006	China	Jiangxi	L8
EU097706	NX06	2006	China	Ningxia	L8
EU097707	BJsy06	2006	China	Beijing	L8
EU106888	SHH	2006	China	Shanghai	L8
EU109502	LN	2006	China	Liaoning	L8
EU109503	GD	2006	China	Guangdong	L8
EU144079	SY0608	2006	China	Jiangsu	L8
EU187484	WUH1	2007	China	Hubei	L8
EU200961	Jiangxi-3	na	China	Jiangxi	L8
EU200962	Henan-1	na	China	Henan	L8
EU236259	HPBEDV	na	China	na	L8
EU262603	Em2007	2007	China	Hubei	L8
EU624117	XH-GD	2007	China	Guangdong	L8
EU678352	WUH2	na	China	Hubei	L8
EU708726	JX143	2006	China	Jiangxi	L8
EU825723	BJ	2007	China	Beijing	L8
EU825724	GD	2007	China	Guangdong	L8
EU860248	TJ	2006	China	Tianjin	L8
EU860249	NM1	2007	China	Inner<br>Mongolia	L8
EU864231	CG	2007	China	na	L8
EU864232	SHB	2005	China	na	L8
EU864233	TP	2006	China	na	L8
EU880431.2	GS2008	2008	China	Gansu	L8
EU880432.2	JX2006	2006	China	Jiangxi	L8
EU880433.2	GD2007	2007	China	Guangdong	L8
EU880434.2	SX2007	2007	China	Shanxi	L8
EU880435.2	YN2008	2008	China	Yunnan	L8
EU880436	XL2008	2008	China	na	L8
EU880437.2	HN2007	2007	China	Hunan	L8
EU880438	CH2002	2002	China	na	L8
EU880439	CH2004	2004	China	na	L8
EU880440	CH2003	2003	China	na	L8
EU880441.2	GS2002	2002	China	Gansu	L5
EU880442.2	GS2003	2003	China	Gansu	L5
EU880443.3	GS2004	2004	China	Gansu	L5
EU939312	JSyx	2006	China	Jiangsu	L8
FJ175687	PRRSV01	na	China	na	L5
FJ175688	PRRSV02	na	China	na	L5
FJ175689	PRRSV03	na	China	na	L5

FJ393456	07NM	2007	China	Inner<br>Mongolia	L8
FJ393457	07HEN	2007	China	Henan	L8
FJ393458	07HEBTJ	2007	China	Hebei	L8
FJ393459	07BJ	2007	China	Beijing	L8
FJ536165	NB/04	2004	China	Zhejiang	L8
FJ797690	HN-HW	2006	China	Hunan	L8
FJ889129	CBB-1-F3	2008	China	na	L8
FJ889130	CWZ-1-F3	2008	China	na	L8
FJ895329	SX2009	2009	China	Shanxi	L8
FJ950744	BJSY-1	2007	China	Beijing	L8
FJ950745	BJBLZ	2007	China	Beijing	L8
FJ950746	BJPG	2007	China	Beijing	L8
FJ950747	BJSD	2007	China	Beijing	L8
GQ330474	APRRS	na	China	na	L7
GQ351601	BJ0706	2007	China	Beijing	L8
GQ359108	SD-CXA/2008	2008	China	Shandong	L8
GQ374441	GDQJ	2007	China	Guangdong	L8
GQ374442	GDBY1	2008	China	Guangdong	L8
GQ857656	SX-1	na	China	Shanxi	L8
GQ914997	SD1-100	na	China	Shandong	L1
GU143913	GD	2009	China	Guangdong	L8
GU168567	09HUB7	2009	China	Hubei	L8
GU168568	09HUB5	2009	China	Hubei	L8
GU168569	08SDWF	2008	China	Shandong	L8
GU169411	08HuN	2008	China	Hunan	L8
GU232735	KP	2008	China	na	L8
GU232738	YN9	2008	China	Yunnan	L8
GU269541	GD3	2005	China	Guangdong	L8
GU454850	GDQY2	2007	China	Guangdong	L8
GU461292	AH0701	2007	China	Anhui	L8
HM011104	BJSY07	2007	China	Beijing	L8
HM016158	JN-HS	2008	China	Shandong	L8
HM016159	ZP-1	2009	China	Shandong	L8
HM189676	HLJHL	2009	China	Heilongjiang	L8
HM214913	GX09-16	2009	China	Guangxi	L8
HM214914	GX09-29	2009	China	Guangxi	L8
HM214915	GX09-32	2009	China	Guangxi	L8
HM853673.2	WUH3	2008	China	Hubei	L8
HQ315835	BB0907	2009	China	Guangxi	L8
HQ315836	NT0801	2008	China	Jiangsu	L8
HQ315837	SY0909	2009	China	Jiangsu	L8
HQ401282	Shaanxi-2	2007	China	Shaanxi	L8

HQ416720	TA-12	2008	China	Shandong	L8
HQ843178	HLJ-09	2009	China	Henan	L8
HQ843179	HLM-09	2009	China	Henan	L8
HQ843180	SD-09	2009	China	Shandong	L8
HQ843181	SX-09	2009	China	Shanxi	L8
JF268672	09SC	2009	China	Sichuan	L8
JF268673	09HUN1	2009	China	Hunan	L8
JF268674	09HUN2	2009	China	Hunan	L8
JF268675	09JS	2009	China	Jiangsu	L8
JF268676	09BJ	2009	China	Beijing	L8
JF268677	09DB1	2009	China	na	L8
JF268678	09SD	2009	China	Shandong	L8
JF268679	09HEB	2009	China	Hebei	L8
JF268680	09HEN2	2009	China	Henan	L8
JF268681	09DB2	2009	China	na	L8
JF268682	09HUB1	2009	China	Hubei	L8
JF268683	09HUB2	2009	China	Hubei	L8
JF268684	09HEN1	2009	China	Henan	L8
JF748717	YD	2009	China	na	L8
JF748718	DC	2010	China	na	L8
JF796180	FS	2010	China	Guangdong	L8
JF796180	FS	2010	China	Guangdong	L8
JF800911	ZCYZ	2009	China	Shandong	L8
JN256115	SD0901	2009	China	Shandong	L8
JN387271	GDQY1	2007	China	Guangdong	L8
JN662424	GM2	2011	China	Guangdong	L3
JN836553	SCwhn09CD	2009	China	Sichuan	L8
JN864948	DY	2007	China	Guangdong	L5
JQ308798	QYYZ	2011	China	Guangdong	L3
JQ309822	GX10-42	2010	China	Guangxi	L8
JQ309823	GX10-48	2010	China	Guangxi	L8
JQ326271	WUH4	2011	China	Hubei	L8
JQ663540	10-10JX	2010	China	Jiangxi	L8
JQ663541	10-10BJ-1	2010	China	Beijing	L8
JQ663542	10-10BJ-3	2010	China	Beijing	L8
JQ663543	10-10BJ-2	2010	China	Beijing	L8
JQ663544	10-10BJ-4	2010	China	Beijing	L8
JQ663545	10-10BJ-5	2010	China	Beijing	L8
JQ663546	10-10FUJ-1	2010	China	Fujian	L8
JQ663547	10-10FUJ-2	2010	China	Fujian	L8
JQ663548	10-10FUJ-3	2010	China	Fujian	L8
JQ663549	10-10FUJ-4	2010	China	Fujian	L8
JQ663550	10-10FUJ-5	2010	China	Fujian	L8

JQ663551	10-10HEB-1	2010	China	Hebei	L8
JQ663552	10-10HEB-2	2010	China	Hebei	L8
JQ663553	10-10HEB-3	2010	China	Hebei	L8
JQ663554	10-10JL	2010	China	Jilin	L8
JQ663555	10-10SD	2010	China	Shandong	L8
JQ663556	10-10QN	2010	China	na	L8
JQ663558	10-10GX-1	2010	China	Guangxi	L8
JQ663559	10-10GX-2	2010	China	Guangxi	L8
JQ663560	10-10GX-3	2010	China	Guangxi	L8
JQ663561	10-10GX-4	2010	China	Guangxi	L8
JQ663562	10-10GX-5	2010	China	Guangxi	L8
JQ715697	NVDC-GD2-2011	2011	China	Guangdong	L8
JQ715698	NVDC-JS2-2011	2011	China	Jiangsu	L8
JQ743666	QY2010	2010	China	Guangdong	L3
JQ955657	GX1001	2010	China	Guangxi	L8
JQ955658	GX1002	2010	China	Guangxi	L8
JX087437	SD16	2012	China	Shandong	L8
JX177644	JL-04/12	2012	China	Jilin	L8
JX192632	10HN-GD	2010	China	Guangdong	L8
JX192633	10GZ-GD	2010	China	Guangdong	L8
JX192634	10FS-GD	2010	China	Guangdong	L8
JX192635	10FS1-GD	2010	China	Guangdong	L8
JX192636	10BY-GD	2010	China	Guangdong	L8
JX192637	10SJ-GD	2010	China	Guangdong	L8
JX192638	10SS-GD	2010	China	Guangdong	L8
JX192639	10ZQ-GD	2010	China	Guangdong	L8
JX215551	11FS11-GD	2011	China	Guangdong	L8
JX215552	10QY-GD	2010	China	Guangdong	L8
JX215553	10HD-GD	2010	China	Guangdong	L8
JX215554	11FS12-GD	2011	China	Guangdong	L8
JX217036	11NZ-GD	2011	China	Guangdong	L8
JX235365	11SH-GD	2011	China	Guangdong	L8
JX235366	11SH1-GD	2011	China	Guangdong	L8
JX235367	11XX-GD	2011	China	Guangdong	L8
JX235370	11GZ-GD	2011	China	Guangdong	L8
JX317648	HV	2007	China	Na	L8
JX317649	JX	2010	China	Jiangxi	L8
JX679179	HH08	2011	China	Heilongjiang	L8
JX857698	YN-2011	2011	China	Yunan	L5
JX878379	SDA2	2011	China	Shandong	L8
JX878380	SDA3	2011	China	Shandong	L8
JX880029	NJ-1106	2011	China	Jiangsu	L8
JX912249	GX1003	2010	China	Guangxi	L8

KC445138	HZ-31	2012	China	na	L5
KC527830	GD-2011	2011	China	Guangdong	L8
KF287132	HK1	2003	China	Hongkong	L8
KF287133	HK2	2003	China	Hongkong	L3
KF287134	HK4	2003	China	Hongkong	L8
KF287135	HK6	2004	China	Hongkong	L3
KF287136	HK7	2004	China	Hongkong	L8
KF287137	HK9	2004	China	Hongkong	L3
KF287138	HK11	2004	China	Hongkong	L3
KF287139	HK12	2004	China	Hongkong	L8
KF287140	HK13	2005	China	Hongkong	L8
KF287141	HK14	2004	China	Hongkong	L5
KF287142	HK15	2004	China	Hongkong	L3
KF287143	HK16	2004	China	Hongkong	L3
KF611905	HENAN-XINX	2013	China	Henan	L1
KF678434	SH1211	2012	China	Shanghai	L3
KF751237	BJ1102	2011	China	Beijing	L8
KF751238	LN1101	2011	China	Liaoning	L8
KF771273	GZ1101	2011	China	Guizhou	L5
KF815525	XJu-1	2012	China	Xinjiang	L8
KJ002451	HeNan-A1	2013	China	Henan	L8
KJ002452	HeNan-A2	2013	China	Henan	L8
KJ019330	Henan-A3	2013	China	Henan	L8
KJ143621	HENAN-HEB	2012	China	Henan	L1
KJ534539	Henan-A4	2013	China	Henan	L8
KJ534540	Henan-A5	2013	China	Henan	L8
KJ534541	Henan-A6	2013	China	Henan	L8
KJ534542	Henan-A7	2013	China	Henan	L8
KJ534543	Henan-A8	2013	China	Henan	L8
KJ541663	GZ106	2005	China	Guizhou	L8
KJ546412	HeNan-A9	2013	China	Henan	L8
KJ591659	HEB-2013	2013	China	Hebei	L8
KJ609516	MY-486	2013	China	Henan	L8
KJ609517	MY-376	2013	China	Henan	L8
KJ747052	YN-1	2011	China	Yunnan	L8
KJ819934	Henan-A12	2014	China	Henan	L8
KJ819935	Henan-A13	2014	China	Henan	L8
KJ819936	Henan-A14	2014	China	Henan	L8
KJ855518	Shanxi-6	2010	China	Shanxi	L8
KM000066	NMG2014	2014	China	Inner<br>Mongolia	L8
KM189443	SC2012	2012	China	Sichuan	L8
KM261784	HB2014001	2014	China	Hubei	L8

KP162169	HB-XL	2014	China	na	L8
KP179402	NT1	na	China	Jiangsu	L8
KP179403	NT2	na	China	Jiangsu	L8
KP179404	NT3	na	China	Jiangsu	L8
KP330232	HUN-2014	2014	China	Hunan	L8
KP742986	TJbd14-1	2014	China	Tianjin	L8
KP742987	TJbd14-2	2014	China	Tianjin	L8
KP771735	NVDC-SHH02-2014	2014	China	Shanghai	L8
KP771736	NVDC-shh01-2014	2014	China	Shanghai	L8
KP771737	NVDC-SD6-2014	2014	China	Shandong	L8
KP771738	NVDC-SD1-2014	2014	China	Shandong	L8
KP771739	NVDC-SC1-2014	2014	China	Sichuan	L8
KP771740	NVDC-SXJC-2013	2013	China	Shanxi	L8
KP771741	NVDC-SDXX-2013	2013	China	Shandong	L8
KP771742	NVDC-HBCZ-2013	2013	China	Hubei	L8
KP771743	NVDC-BJPG-2013	2013	China	Beijing	L8
KP771744	NVDC-HeB2-2013	2013	China	Hebei	L8
KP771745	NVDC-HeB1-2013	2013	China	Hebei	L8
KP771746	NVDC-CQ1-2011	2011	China	Chongqing	L8
KP771747	NVDC-CQ1-2012	2012	China	Chongqing	L8
KP771748	NVDC-BJ2-2011	2011	China	Beijing	L8
KP771749	NVDC-HeB1-2011	2011	China	Hebei	L8
KP771750	NVDC-MD2-2013	2013	China	na	L8
KP771751	NVDC-MD1-2013	2013	China	na	L8
KP771752	HEB 20130008-14	2013	China	Hebei	L8
KP771753	HEB 20130008-13	2013	China	Hebei	L8
KP771754	NVDC-HeB-2008	2008	China	Hebei	L8
KP771755	NVDC-CQ-2008	2008	China	Chongqing	L8
KP771756	NVDC-BJ9-2012	2012	China	Beijing	L8
KP771757	NVDC-BJ8-2012	2012	China	Beijing	L8
KP771758	NVDC-BJ7-2012	2012	China	Beijing	L8
KP771759	NVDC-BJ6-2012	2012	China	Beijing	L8
KP771760	NVDC-BJ5-2012	2012	China	Beijing	L8
KP771761	NVDC-BJ4-2012	2012	China	Beijing	L8
KP771762	NVDC-BJ3-2012	2012	China	Beijing	L8
KP771763	NVDC-BJ2-2012	2012	China	Beijing	L8
KP771764	NVDC-BJ1-2012	2012	China	Beijing	L8
KP771765	NVDC-HeB2-2011	2011	China	Hebei	L8
KP771766	NVDC-GD-2011	2011	China	Guangdong	L8
KP771767	NVDC-YN-2011	2011	China	Yunnan	L8
KP771768	NVDC-SD2-2012	2012	China	Shandong	L8
KP771769	NVDC-SD1-2012	2012	China	Shandong	L8
KP771770	NVDC-HuN-2011	2012	China	Hunan	L8

KP771771	NVDC-HeN-2012	2012	China	Henan	L8
KP771772	NVDC-HeB2-2012	2012	China	Hebei	L8
KP771773	NVDC-HeB1-2012	2012	China	Hebei	L8
KP771774	NVDC-CQ3-2011	2011	China	Chongqing	L8
KP771775	NVDC-CQ3-2012	2012	China	Chongqing	L8
KP771776	NVDC-CQ2-2012	2012	China	Chongqing	L8
KP771777	NVDC-CQ4-2012	2012	China	Chongqing	L8
KP771778	NVDC-BJ1-2011	2011	China	Beijing	L8
KP771779	NVDC-NM-2008	2008	China	Inner Mongolia	L8
KP771780	NVDC-13SXJC-2014	2014	China	Shanxi	L8
KP771781	NVDC-HuNCS-2014	2014	China	Hunan	L8
KP771782	NVDC-R38-2014	2014	China	na	L8
KP771783	NVDC-R224-2014	2014	China	na	L8
KP771784	NVDC-SD4-2014	2014	China	Shandong	L8
KP780881	14LY01-FJ	2014	China	Fujian	L8
KP780882	14LY02-FJ	2014	China	Fujian	L8
KP793736	GD-HD	2011	China	Guangdong	L8
KP860909	FJZ03	na	China	Fujian	L1
KP860910	FJY04	na	China	Fujian	L1
KP860911	FJW05	na	China	Fujian	L1
KP861625	CHsx1401	2014	China	Shanxi	L1
KP998401	17199	2005	China	Taiwan	L3
KP998402	312	2005	China	Taiwan	L3
KP998403	1483	2012	China	Taiwan	L3
KP998404	338	2011	China	Taiwan	L3
KP998406	HC120821-SH1	2012	China	Taiwan	L3
KP998407	HC120821-SH2	2012	China	Taiwan	L3
KP998408	HC120821-LL	2012	China	Taiwan	L3
KP998409	HC120904-CHYL	2012	China	Taiwan	L3
KP998410	JM	2011	China	Taiwan	L3
KP998412	25934	2008	China	Taiwan	L3
KP998413	310	2005	China	Taiwan	L3
KP998414	660	2009	China	Taiwan	L3
KP998415	803	2013	China	Taiwan	L3
KP998416	CH	2002	China	Taiwan	L3
KP998418	HL	2004	China	Taiwan	L3
KP998419	M1	2001	China	Taiwan	L3
KP998420	NT	2000	China	Taiwan	L3
KP998421	Q94-136	2005	China	Taiwan	L3
KP998422	TD/TP	1998	China	Taiwan	L3
KP998423	Tsai	1999	China	Taiwan	L3
KP998424	TY1	2000	China	Taiwan	L3

KP998425	TD1	1997	China	Taiwan	L3
KP998426	CH8V-J2	2003	China	Taiwan	L3
KP998427	TD-2	2004	China	Taiwan	L3
KP998429	WSV	1992	China	Taiwan	L5
KP998430	HC120629	2012	China	Taiwan	L3
KP998431	MD001	1991	China	Taiwan	L3
KP998474	FJSD	na	China	Fujian	L5
KP998475	FJE1	na	China	Fujian	L8
KP998476	FJFS	na	China	Fujian	L3
KP998477	FJCH	na	China	Fujian	L8
KP998478	FJZH	na	China	Fujian	L8
KP998479	FJOU	na	China	Fujian	L8
KR149645	JXja15	2015	China	Jiangxi	L8
KR706343	JL580	2013	China	Jilin	L1
KT022071	HNxa14	2014	China	Hunan	L8
KT022072	HNyc13	2013	China	Hunan	L8
KT180169	XF1129	2013	China	na	L8
KT351739	HLJA1	2013	China	Heilongjiang	L8
KT351740	HLJB1	2013	China	Heilongjiang	L8
KT358728	GZgy15-1	2015	China	Guizhou	L8
KT445876	HNP5	2014	China	na	L8
KT804696	FJYR	na	China	Fujian	L8
KT819203	SCwhn14DY	2014	China	Sichuan	L8
KT945017	HNjz15	2015	China	Henan	L1
KT945018	HNyc15	2015	China	Henan	L3
KU201579	QH-08	2008	China	Qinghai	L8
KU215416	15LY01-FJ	2015	China	Fujian	L8
KU215417	15LY02-FJ	2015	China	Fujian	L8
KU523366	WUH5	2015	China	Hubei	L1
KU523367	WUH6	2011	China	Hubei	L1
KU950370	HENPDS-2	2015	China	Henan	L8
KU950371	HENXC-4	2015	China	Henan	L1
KU950372	HENXX-1	2014	China	Henan	L1
KU950373	HENZK-1	2014	China	Henan	L8
KU950374	HENZMD-9	2015	China	Henan	L1
KU950375	HENZZ-8	2015	China	Henan	L8
KU978619	GD-KP	2015	China	Guangdong	L3
KX169191	FJ1402	2014	China	Fujian	L1
KX357708	QTX	2016	China	na	L8
KX510269	TJnh1501	2015	China	Tianjin	L8
KX621003	GDsg	2015	China	Guangdong	L3
KX650082	RVB-581	2008	China	na	L8
KX689233	XJzx1-2015	2015	China	Xinjiang	L3

KX758249	FJWQ16	2016	China	Fujian	L1
KX758250	FJXS15	2015	China	Fujian	L1
KX766378	HN07-1	2007	China	Hunan	L8
KX766379	HNhx	2016	China	Hunan	L1
KX767091	GSWW/CHA 2015	2015	China	Gansu	L8
KX815407	15GD1	2015	China	Guangdong	L8
KX815408	15GD2	2015	China	Guangdong	L8
KX815409	15GD3	2015	China	Guangdong	L8
KX815410	15GD4	2015	China	Guangdong	L8
KX815411	15HEB1	2015	China	Hebei	L1
KX815412	15HEB3	2015	China	Hebei	L8
KX815413	15HEN1	2015	China	Henan	L1
KX815414	15HEN3	2015	China	Henan	L8
KX815415	15HEN4	2015	China	Henan	L1
KX815416	15HUN1	2015	China	Hunan	L8
KX815417	15HUN2	2015	China	Hunan	L8
KX815418	15HUN3	2015	China	Hunan	L8
KX815419	15JX1	2015	China	Jiangxi	L1
KX815420	15JX2	2015	China	Jiangxi	L8
KX815421	15JX3	2015	China	Jiangxi	L8
KX815422	15JX4	2015	China	Jiangxi	L8
KX815423	15LN1	2015	China	Liaoning	L1
KX815424	15LN2	2015	China	Liaoning	L8
KX815425	15LN3	2015	China	Liaoning	L1
KX815426	15SC1	2015	China	Sichuan	L8
KX815427	15SC2	2015	China	Sichuan	L8
KX815428	15SC3	2015	China	Sichuan	L1
KX815429	15SN1	2015	China	Shannxi	L8
KX815430	15SN2	2015	China	Shannxi	L8
KX815431	15SN3	2015	China	Shannxi	L8
KX815432	15ZJ1	2015	China	Zhejiang	L1
KX815433	15ZJ2	2015	China	Zhejiang	L8
KX815434	15ZJ3	2015	China	Zhejiang	L8
KX900392	HENJY-2	2015	China	Henan	L1
KX980392	SDhz1512	2015	China	Shandong	L1
KX980393	SDlz1601	2016	China	Shandong	L8
KY041782	HENXX-8	2016	China	Henan	L1
KY053458	SDYG1606	2016	China	Shandong	L1
KY290748	HENXX-9	2016	China	Henan	L8
KY373214	JSWA	2014	China	Jiangsu	L1
KY373215	HiNZWQ	2014	China	Hainan	L3
KY373216	AHBZ	2014	China	Anhui	L8
KY373217	SDZZ	2014	China	Shandong	L8

KY373218	SXF105	2014	China	Shanxi	L8
KY412887	FJL15	2014	China	Fujian	L1
KY412888	FJM4	2014	China	Fujian	L1
KY488470	GDJM	2014	China	Guangdong	L8
KY488471	GDGZ	2014	China	Guangdong	L8
KY488472	GDMM	2014	China	Guangdong	L8
KY488473	GDZQ	2014	China	Guangdong	L8
KY488474	HNHK2	2014	China	Hunan	L8
KY488475	HNHK1	2014	China	Hunan	L8
KY488476	GDHY	2015	China	Guangdong	L8
KY488477	GDHZ	2015	China	Guangdong	L8
KY488478	GDQY	2015	China	Guangdong	L8
KY488479	GDSG	na	China	Guangdong	L8
KY495780	JX/CH/2016	2016	China	Jiangxi	L3
KY495781	SH/CH/2016	2016	China	Shanghai	L3
KY498542	GDST	2014	China	Guangdong	L8
KY745901	GDYDZZZ	2016	China	Guangdong	L3
KY761966	FZ16A	2016	China	Fujian	L8
MF124329	GD1404	2014	China	Guangdong	L3
MF196905	SCcd16	2016	China	Sichuan	L3
MF196906	SCnj16	2016	China	Sichuan	L1
MF370557	FZ06A	2006	China	Fujian	L8
MF375260	SD-A19	2015	China	Shandong	L1
MF375261	SC-d	2015	China	Sichuan	L1
MF526896	GDQYQC2	2016	China	Guangdong	L8
MF669720	GD1404	2014	China	Guangdong	L8
MF669721	HZL1501	2015	China	Na	L8
MF669722	ZJXS1412	2014	China	Zhejiang	L8
MF689000	HeN1201	2012	China	Henan	L8
MF766470	HeN1301	2013	China	Henan	L8
MF766471	HeN1401	2014	China	Henan	L1
MF766472	HeN1501	2015	China	Henan	L8
MF766473	HeN1502	2015	China	Henan	L8
MF766474	HeN1601	2016	China	Henan	L1
MF770574	17-ZJ-HZ	2017	China	Zhejiang	L8
MF772778	GDzj	na	China	Guangdong	L8
MF818049	SC/NJ 2016	2016	China	Sichuan	L8
MG011718	FJLIUY-2017	2017	China	Fujian	L3
MG011719	FJDJQ-2017	2017	China	Fujian	L3
MG687491	QHD1	2017	China	Qinghai	L1
MG844181	HB17A	2017	China	Hebei	L1
MG860516	LNWK96	2017	China	Liaoning	L1
MG913987	LNWK130	2017	China	Liaoning	L1

MG914067	SCcd17	2017	China	Sichuan	L1
MH046842	FJNP2017	2017	China	Fujian	L3
MH046843	GDZS2016	2016	China	Guangdong	L3
MH068878	SD17-38	2017	China	Shandong	L1
MH078490	SCN17	2017	China	Sichuan	L1
MH121061	SD17-36	2017	China	Guangdong	L1
MH167387	QHD2	2017	China	Qinghai	L1
MH167388	QHD3	2017	China	Qinghai	L1
MH236426	ZJnb16-2	2016	China	Zhejiang	L3
MH324400	SCya17	2017	China	Sichuan	L3
MH370474	CH/2018/NCV-Anheal-1	2018	China	na	L1
MH404256	SD17	2017	China	Shandong	L8
MH588710	SDbz16-2	2016	China	Shandong	L1
MH651736	CY1-1604	2016	China	na	L1
MH651737	CY2-1604	2016	China	na	L1
MH651738	HNJYF-1606	2016	China	Hunan	L1
MH651739	HBFL-1604	2016	China	Hubei	L1
MH651740	HNJYH-1606	2016	China	Hunan	L1
MH651741	LNCH-1604	2016	China	Liaoning	L1
MH651742	SDQD-1604	2016	China	Shandong	L1
MH651743	SD-1602	2016	China	Shandong	L1
MH651744	SD53-1603	2016	China	Shandong	L1
MH651745	SD99-1606	2016	China	Shandong	L1
MH651746	SDQZ-1609	2016	China	Shandong	L1
MH651747	SDZC-1609	2016	China	Shandong	L8
MH651748	TJZH-1607	2016	China	Tianjin	L1
MH663433	HNRZ	2017	China	Hunan	L8
MK144542	GZgy17	2017	China	Guizhou	L3
MK144543	SCya18	2018	China	Sichuan	L3
MK202794	FJ0908	2018	China	Fujian	L1
MK396376	GDsf1707	2017	China	Guangdong	L1
MK396377	GDsf1710	2017	China	Guangdong	L1
MK396378	GDsf1711	2017	China	Guangdong	L1
MK396379	GDsf1802	2018	China	Guangdong	L1
MK396380	GDsf1804	2018	China	Guangdong	L1
MK396381	GDsf1806	2018	China	Guangdong	L1
MK396382	GDsf1807	2018	China	Guangdong	L1
MK396383	GDsf1808	2018	China	Guangdong	L1
MK396384	GDsf1809	2018	China	Guangdong	L1
MK429980	SWU/MS2/2018	2018	China	Sichuan	L1
MK429981	SWU/MS3/2018	2018	China	Sichuan	L1
MK429982	SWU/MY5/2018	2018	China	Sichuan	L1
MK429983	SWU/MY6/2018	2018	China	Sichuan	L1

MK429984	SWU/YB1/2018	2018	China	Sichuan	L1
MK429985	SWU/YB2/2018	2018	China	Sichuan	L1
MK429986	SWU/CD1/2018	2018	China	Sichuan	L1
MK429987	SWU/CQ1/2018	2018	China	Chongqing	L5
MK450333	CH-WH-2019-1	2018	China	Hubei	L1
MK450365	CH-YY	2018	China	na	L8
MK453049	PRRSV-ZDXYL-China-2018-1	2018	China	Heilongjiang	L1
MK453050	PRRSV-ZDXYL-China-2018-2	2018	China	Heilongjiang	L1
MK759853	XJ17-5	2017	China	Xinjiang	L8
MK780824	SDWH27-1710	2017	China	Shandong	L3
MK780825	SD110-1608	2016	China	Shandong	L3
MK906026	JSTZ1712-12	2017	China	Jiangsu	L8
MN026346	GXBB16-1	2016	China	Guangxi	L8
MN026347	NM-12	na	China	na	L8
MN046221	2014-81	2014	China	Heilongjiang	L8
MN046222	HLJ-80	2016	China	Heilongjiang	L1
MN046223	HLJ-DZD1-1804	2018	China	Heilongjiang	L8
MN046224	HEB-108	2017	China	Hebei	L1
MN046225	Fujian-2014-18	2014	China	Fujian	L1
MN046226	HeNXX-2014-3	2014	China	Henan	L1
MN046227	HeNXX-2014-9	2014	China	Henan	L8
MN046228	HeNXX-2014-12	2014	China	Henan	L1
MN046229	HeB-239	2018	China	Hebei	L1
MN046230	HLJWK108-1711	2018	China	Heilongjiang	L1
MN046231	Anhui-2017-109	2017	China	Anhui	L8
MN046232	JS3-1805	2018	China	Jiangsu	L8
MN046233	Liaoning-2017-6	2017	China	Liaoning	L8
MN046234	SDJM-1602	2016	China	Shandong	L8
MN046235	Gansu-2017-51	2017	China	Gansu	L8
MN046236	Sichuan-2017-117	2017	China	Sichuan	L8
MN046237	InterMo-2015-2	2015	China	Inner Mongolia	L8
MN046238	HN-1603	2016	China	Hunan	L8
MN046239	HLJ-YC8	2018	China	Heilongjiang	L8
MN046240	SX1-1607	2016	China	Shanxi	L1
MN046241	SX2-1607	2016	China	Shanxi	L1
MN046242	LN-DB87	2018	China	Liaoning	L3
MN046243	HLJ-DZD4-1805	2018	China	Heilongjiang	L1
MN119304	SD1612-1	2016	China	Shandong	L8
MN119305	SD1704-23	2017	China	Shandong	L1
MN119306	SH1704-25	2017	China	Shanghai	L8
MN119307	HN1804-2	2018	China	Henan	L1
MN119308	JS1810-195	2018	China	Jiangsu	L8

MN119309	XJ1904-39	2019	China	Xinjiang	L1
MN401750	SW2018001-YL	2018	China	Taiwan	L3
MN547964	JS1703-21	2017	China	Jiangsu	L8
MN547965	JSTZ1810-220	2018	China	Jiangsu	L1
MN547966	JSTZ1904-664	2019	China	Jiangsu	L8
MN547967	JSTZ1907-714	2019	China	Jiangsu	L8
MN606304	JS18-3	2018	China	Jiangsu	L1
MN606305	JX07	2007	China	Jiangxi	L8
MN642099	SDqd1501	2015	China	Shandong	L3
MN642100	SDwh1402	2014	China	Shandong	L8
MN642101	SDwh1403	2014	China	Shandong	L1
MN642102	SDwh1601	2016	China	Shandong	L1
MN642103	SDwh1602	2016	China	Shandong	L8
MN642104	SDwh1701	2017	China	Shandong	L5
MN642105	SDyt1401	2014	China	Shandong	L1
MN648054	LNDZD10-1806	2018	China	Liaoning	L1
MN648055	HLJZD30-1902	2019	China	Heilongjiang	L1
MN648449	HLHDZD32-1901	2019	China	Heilongjiang	L1
MN648450	HLJZD22-1812	2018	China	Heilongjiang	L1
MN660067	GXNN1396-p3	2013	China	Guangxi	L8
MN660069	GXYL1403	2014	China	Guangxi	L8
MN660070	GXNN1839	2018	China	Guangxi	L1
MN862433	FJDJQ-2018	2018	China	Fujian	L1
MT036897	FS-GD-02_FoShan_Guangdong_2016.01	2016	China	Guangdong	L1
MT036898	GDHZ_Huizhou	2017	China	Guangdong	L1
MT036899	HZ1-3_Yunnan_20171	2017	China	Yunnan	L1
MT036900	XY-HN_Xinyang	2017	China	Henan	L1
MT075480	SC/DJY	2019	China	Sichuan	L1
MT165636	GD1909	2019	China	Guangdong	L1
MT268280	HB18-41	2018	China	Hubei	L8
MT316312	HB18-4	2019	China	Hubei	L8
MT379661	GDDX-2018	2018	China	Guangdong	L8
MT394494	GDhh1808	2018	China	Guangdong	L8
MT394495	GDxn1808	2018	China	Guangdong	L1
MT394496	GDsc1808	2018	China	Guangdong	L1
MT394497	GDsc1809	2018	China	Guangdong	L1
MT409687	PRRSV2/CN/X4831/2018	2018	China	na	L1
MT409688	PRRSV2/CN/X9830/2018	2018	China	na	L1
MT409689	PRRSV2/CN/X4836/2018	2018	China	na	L1
MT409690	PRRSV2/CN/X2998/2018	2018	China	na	L8
MT409691	PRRSV2/CN/X4833/2018	2018	China	na	L1
MT409692	PRRSV2/CN/X4839/2017	2017	China	na	L1
MT416541	PRRSV2/CN/X2984/2018	2018	China	na	L8

MT416542	PRRSV2/CN/N9185/2018	2018	China	na	L3
MT416543	PRRSV2/CN/F1228/2017	2017	China	na	L8
MT416544	PRRSV2/CN/F1004/2017	2017	China	na	L3
MT416545	PRRSV2/CN/101806/2018	2018	China	na	L8
MT416546	PRRSV2/CN/110713/2018	2018	China	na	L1
MT416548	PRRSV2/CN/101805/2018	2018	China	na	L5
MT663768	TS01	2018	China	na	L8
MT708500	SD-YL1712	2017	China	Shandong	L8
MT721741	PRRSV2/CN/GDDX/2018	2018	China	Guangdong	L8
MT746146	JSYC20-05-1	2020	China	Jiangsu	L5
MT780871	JSYZ1909-16	2019	China	Jiangsu	L8
MT811822	YNAN2018	2018	China	Yunnan	L8
MT811823	YNCN2017	2017	China	Yunnan	L8
MT811824	YNWH2016	2017	China	Yunnan	L8
MT811825	YNCL2018	2018	China	Yunnan	L8
MT811826	YNCXZX2017	2017	China	Yunnan	L8
MT811827	YNCX2016	2016	China	Yunnan	L8
MT811828	YNDL2016	2017	China	Yunnan	L8
MT811829	YNJN2016	2016	China	Yunnan	L8
MT811830	YNYL2016	2017	China	Yunnan	L8
MT811831	YNLQ2016	2016	China	Yunnan	L8
MT811832	YNQJXW2017	2017	China	Yunnan	L8
MT811833	YNQJ2017	2017	China	Yunnan	L8
MT811834	YNSD2017	2017	China	Yunnan	L8
MT811835	YNSL2018	2018	China	Yunnan	L8
MT811836	YNSM2016	2016	China	Yunnan	L8
MT811837	YNSB2016	2017	China	Yunnan	L8
MT811838	YNPL2016	2016	China	Yunnan	L8
MT811839	YNXS2017	2017	China	Yunnan	L8
MT811840	YNXW2017	2017	China	Yunnan	L8
MT811841	YNML2018	2018	China	Yunnan	L8
MW079495	2020-Acheng-1	2020	China	Heilongjiang	L1
MW531679	GXNN202004a	2020	China	Guangxi	L1
MW561593	GXNN202010	2020	China	Guangxi	L3
MW561594	GXNN202004	2020	China	Guangxi	L1
MW627193	HB18-36	2018	China	Hubei	L8
MW651975	HB19-12	2019	China	Hubei	L8
MW651976	HB19-18	2019	China	Hubei	L8
MW803134	PRRSV-China/SCcd2020/2020	2020	China	Sichuan	L1
MW853923	AH-PRRS20178-1	2017	China	Anhui	L8
MW880772	SXSZ-2020	2020	China	Shanxi	L1
MZ172971	SD-QD-2101	2020	China	Shandong	L8
MZ322956	SDRZ01	2019	China	Shandong	L8

MZ342900	JS2020	2020	China	Jiangsu	L1
MZ579701	HBap4/2018	2018	China	Hubei	L8
MZ820388	JS2021NADC34	2021	China	Jiangsu	L1
KC862575	DK-2012-01-11-3	2012	Denmark	na	L5
KC862576	DK-1997-19407B	1997	Denmark	na	L5
KC862577	DK-2011-030311-1	2011	Denmark	na	L5
KC862578	DK-2004-1-7-Pl	2004	Denmark	na	L5
KC862579	DK-2010-10-1-2	2010	Denmark	na	L5
KC862580	DK-2010-10-7-1	2010	Denmark	na	L5
KC862581	DK-2010-10-2-1	2010	Denmark	na	L5
KC862582	DK-2008-10-1-3	2008	Denmark	na	L5
KC862583	DK-2010-10-4-1	2010	Denmark	na	L5
KC862584	DK-2003-2-3	2003	Denmark	na	L5
KC862585	DK-2004-2-1	2004	Denmark	na	L5
KF183946	DK-2010-10-13-1	2010	Denmark	na	L5
KF183947	DK-2011-88005-A8-Pl	2011	Denmark	na	L5
KM514315	PRRSV-2 102	2012	Hungary	na	L1
MK287894	PRRSV/MZ/IND/1A/18	2018	India	na	L8
MK287895	PRRSV/MZ/IND/24-A/18	2018	India	na	L8
MK315208	PRRSV/MZ/IND/399A/18	2018	India	na	L8
MK315209	PRRS/MZ/IND/DBT1/18	2018	India	na	L8
MK315210	PRRS/MZ/IND/109A/18	2018	India	na	L8
AB288356	EDRD-1	1992	Japan	Chiba	L4
AB811786	Nagasaki11-14	2011	Japan	Nagasaki	L9
AB811787	Jam2	2000	Japan	Aomori	L5
AB811788	Yamagata10-7	2010	Japan	Yamagata	L4
AB811789	Aomori10-5	2010	Japan	Aomori	L4
LC008532	Jpn5-37	na	Japan	na	L1
JN626287	BH58/10	2010	Laos	na	L8
JQ663557	10-LW1-13	2010	Laos	na	L8
JQ663563	10-LW2-6	2010	Laos	na	L8
JQ663564	10-LW3-7	2010	Laos	na	L8
JQ663565	10-10LW5-1	2010	Laos	na	L8
JQ663566	10-LW6-6	2010	Laos	na	L8
JQ663567	10-LW7-1	2010	Laos	na	L8
JQ663568	10-LW8-1	2010	Laos	na	L8
AY585241	PL97-1	1997	South<br>Korea	na	L5
DQ473474	LMY	na	South<br>Korea	na	L5
JX138233	CP07-626-2	2007	South<br>Korea	na	L1
JX138234	e417-2	2009	South	na	L4

JX138235	CP07-401-9	2007	Korea<br>South	na	L5
JX138236	A4699	2010	Korea<br>South	na	L1
KF555450	CA-2	2013	Korea<br>South	na	L1
KF555451	KNU-12-KJ4	2013	Korea<br>South	na	L1
KP704287	CVK3-6	na	Korea<br>South	na	L1
MK057529	KU-N1202	2012	Korea<br>South	na	L5
MK057530	KU-N1606	2016	Korea<br>South	na	L1
MK057531	KU-N1702	2017	Korea<br>South	na	L1
MK057532	KU-N1712	2017	Korea<br>South	na	L5
DQ056373	01NP1.2	na	Korea<br>Thailand	na	L5
KF735060	HP/Thailand/19500LL/2010	2010	Thailand	na	L8
AF325691	NVSL 97-7985 IA 1-4-2	1997	USA	Iowa	L8
AY545985	NVSL 97-7895	na	USA	na	L8
DQ176019	MN184A	na	USA	Minnesota	L1
DQ176020	MN184B	na	USA	Minnesota	L1
EF532805	Flagship_Before	na	USA	na	L1
EF532808	FF3_Before	na	USA	na	L1
EF532809	FF4_After	na	USA	na	L1
EF532813	Hawkeye4_Before	na	USA	na	L1
EF532816	ISU-P	na	USA	na	L5
EF536000	MN30100	na	USA	Minnesota	L9
EF536001	QUAL1_Before	na	USA	na	L5
EF536002	QUAL2_After	na	USA	na	L1
HQ699067	NC16845	2006	USA	na	L9
JN654458	SDSU73	1996	USA	na	L8
JN654459	NADC30	2008	USA	Iwoa	L1
JN660150	NADC31	2008	USA	Iwoa	L1
JQ087873	A2MC2	2010	USA	na	L5
JX044140	VR2385	na	USA	na	L5
JX258843	SD23983	na	USA	na	L5
KC469618	SD95-21	1995	USA	na	L5
KF632717	XW001	2012	USA	na	L1
KF724397	XW004	2011	USA	na	L1

KF724399	XW011	2012	USA	na	L9
KF724403	XW007	2012	USA	na	L9
KF724404	XW008	2012	USA	na	L2/L8
KF724406	XW014	2011	USA	na	L9
KF724407	XW019	2013	USA	na	L6
KF724408	XW018	2013	USA	na	L1
KF724409	XW015	2013	USA	na	L1
KF724410	XW016	2013	USA	na	L1
KF724411	XW017	2013	USA	na	L1
KF724412	XW006	2013	USA	na	L1
KF724413	XW002	2013	USA	na	L5
KP283399	MN11B	2012	USA	Minnesota	L1
KP283400	MN9B	2012	USA	Minnesota	L1
KP283401	MN6	2012	USA	Minnesota	L1
KP283402	Minnesota17B	2013	USA	Minnesota	L1
KP283403	Minnesota17A	2012	USA	Minnesota	L1
KP283404	Minnesota16	2011	USA	Minnesota	L1
KP283405	Minnesota15	2012	USA	Minnesota	L1
KP283406	Minnesota14	2012	USA	Minnesota	L1
KP283407	Minnesota11A	2012	USA	Minnesota	L1
KP283408	Minnesota9A	2012	USA	Minnesota	L1
KP283409	Minnesota7	2012	USA	Minnesota	L1
KP283410	Minnesota5	2012	USA	Minnesota	L1
KP283411	Minnesota4	2012	USA	Minnesota	L1
KP283412	Minnesota3	2012	USA	Minnesota	L1
KP283413	Minnesota2	2012	USA	Minnesota	L1
KP283414	Minnesota1	2012	USA	Minnesota	L1
KP283415	Iowa12	2012	USA	Iowa	L1
KP283416	Illinois8	2012	USA	Illinois	L1
KR534893	OH28372-2013	2013	USA	Ohio	L1
KR534894	OH155-2015	2015	USA	Ohio	L1
KT207837	12-39404	2012	USA	na	L9
KT257944	14-60	2014	USA	Iowa	L5
KT257945	14-64	2014	USA	Iowa	L5
KT257946	14-67	2014	USA	Iowa	L5
KT257947	14-68	2014	USA	Iowa	L5
KT257948	14-76	2014	USA	Minnesota	L8
KT257950	14-79	2014	USA	Minnesota	L8
KT257952	14-95	2014	USA	Minnesota	L8
KT257953	14-96	2014	USA	Minnesota	L8
KT257954	21675	2014	USA	Kansas	L1
KT257955	101416	2014	USA	Colorado	L1
KT257956	103837	2014	USA	Kansas	L1

KT257958	109560	2014	USA	Kansas	L1
KT257959	ISU01	2014	USA	na	L1
KT257962	ISU04	2014	USA	na	L1
KT257963	ISU05	2014	USA	na	L1
KT257964	ISU06	2014	USA	na	L1
KT257965	ISU07	2014	USA	na	L1
KT257966	ISU10	2014	USA	North<br>Carolina	L1
KT257967	ISU17	2014	USA	Iowa	L1
KT257968	ISU18	2014	USA	Iowa	L1
KT257970	ISU22	2014	USA	na	L1
KT257971	ISU23	2014	USA	na	L1
KT257972	ISU24	2014	USA	na	L1
KT257973	ISU25	2014	USA	na	L1
KT257974	ISU27	2014	USA	na	L1
KT257975	ISU28	2014	USA	na	L1
KT257976	ISU29	2014	USA	na	L1
KT257977	ISU30	2014	USA	na	L1
KT257978	ISU32	2014	USA	na	L1
KT257980	ISU37	2014	USA	na	L1
KT257981	ISU39	2014	USA	na	L1
KT257982	ISU40	2014	USA	na	L1
KT257983	ISU47	2014	USA	Iowa	L1
KT257984	ISU49	2014	USA	na	L1
KT257986	ISU67	2014	USA	na	L1
KT257987	ISU68	2014	USA	Iowa	L1
KT257990	ISU71	2014	USA	na	L1
KT257991	ISU72	2014	USA	na	L1
KT257992	ISU73	2014	USA	Missouri	L1
KT257993	ISU78	2014	USA	Iowa	L1
KT257994	ISU81	2014	USA	Nebraska	L1
KT257995	ISU82	2014	USA	Nebraska	L1
KT257996	ISU84	2014	USA	Nebraska	L1
KT257997	ISU86	2014	USA	Nebraska	L1
KT257998	ISU87	2014	USA	Nebraska	L1
KT257999	ISU90	2014	USA	Colorado	L1
KT258000	ISU91	2014	USA	Colorado	L1
KT258001	ISU94	2014	USA	Iowa	L1
KT258002	ISU96	2014	USA	Iowa	L1
KT258003	ISU97	2014	USA	Iowa	L1
KT258004	ISU95	2014	USA	Iowa	L1
KT258006	SDSU58	2014	USA	North<br>Carolina	L1

KT258007	SDSU62	2014	USA	Nebraska	L1
KT258009	SDSU47	2014	USA	Iowa	L1
KT581982	MN414	2014	USA	Minnesota	L1
KX192112	NCV-13	2016	USA	Nebraska	L1
KX192113	NCV-16	2016	USA	Nebraska	L1
KX192114	NCV-17	2016	USA	Nebraska	L1
KX192115	NCV-21	2016	USA	Nebraska	L1
KX192116	NCV-23	2016	USA	Nebraska	L1
KX192117	NCV-24	2016	USA	Nebraska	L1
KX192118	NCV-25	2016	USA	Nebraska	L1
KX192119	NCV-26	2016	USA	Nebraska	L1
KX462792	A2MC2	2012	USA	na	L5
KY348847	1692-98	1998	USA	na	L9
KY348848	18066-04	2004	USA	na	L1
KY348849	18565-01	2001	USA	na	L1
KY348850	21599-00	2000	USA	na	L9
KY348851	43807-00	2000	USA	na	L5
KY348852	46517-00	2000	USA	na	L9
KY348853	3805-00	2000	USA	na	L5
MF326985	IA/2014/NADC34	2014	USA	Iowa	L1
MF326986	IA/2015/NADC35	2015	USA	Iowa	L1
MF326987	IA/2015/NADC36	2015	USA	Iowa	L1
MF326988	IA/2013/ISU-1	2013	USA	Iowa	L1
MF326989	IA/2014/ISU-2	2014	USA	Iowa	L1
MF326990	NC/2014/ISU-3	2014	USA	North<br>Carolina	L1
MF326991	NC/2014/ISU-4	2014	USA	North<br>Carolina	L1
MF326992	IN/2014/ISU-5	2014	USA	Indiana	L1
MF326993	OH/2014/ISU-6	2014	USA	Ohio	L1
MF326994	OH/2014/ISU-7	2014	USA	Ohio	L1
MF326995	IA/2014/ISU-8	2014	USA	Iowa	L1
MF326996	IA/2015/ISU-9	2015	USA	Iowa	L1
MF326997	IA/2015/ISU-10	2015	USA	Iowa	L1
MF326998	NC/2015/ISU-11	2015	USA	North<br>Carolina	L1
MF326999	NC/2015/ISU-12	2015	USA	North<br>Carolina	L1
MF327000	IA/2015/ISU-13	2015	USA	Iowa	L1
MF327001	IA/2015/ISU-14	2015	USA	Iowa	L1
MF526964	USA/IN_Purdue/1490-LN/2017	2017	USA	Indiana	L1
MF526965	USA/IN_Purdue/14067/2017	2017	USA	Indiana	L1
MF663706	IA14737-2016	2016	USA	na	L1

MK680895	USA/OklahomaSHM/2016	2016	USA	na	L1
MK796164	IA76950-WT	2018	USA	na	L1
MK796165	IA70388-R	2018	USA	na	L8
MK820651	P129	1995	USA	Indiana	L6
MK837936	NADC20	1996	USA	na	L9
MK860181	394-1	2015	USA	North<br>Carolina	L1
MN073081	0752R-S4	2018	USA	na	L1
MN073082	7710R-S18	2018	USA	na	L1
MN073083	7498R-S10	2018	USA	na	L1
MN073084	2385R-S13	2018	USA	na	L1
MN073085	018560PRRS-S9-L001	na	USA	na	L1
MN073086	1924R-S2-L001	2017	USA	na	L1
MN073087	018561PRRS-S10-L001	na	USA	na	L1
MN073088	1923R-S1-L001	2017	USA	na	L1
MN073089	9337R-S4-L001	2017	USA	na	L1
MN073090	PRR223341-S23-L001	na	USA	na	L1
MN073091	PRR223343-S24-L001	na	USA	na	L1
MN073092	9982R-S5-L001	2017	USA	na	L1
MN073093	PRR21032-S1-L001	na	USA	na	L1
MN073094	brian27950-S16-L001	na	USA	na	L1
MN073095	20170210381-S2-L001	2017	USA	na	L1
MN073096	PRRSV-2018014208-S12	2018	USA	na	L1
MN073097	5606R-S6-L001	2017	USA	na	L1
MN073098	PRR312824-S16-L001	2017	USA	na	L1
MN073099	PRR312825-S17-L001	2017	USA	na	L1
MN073100	PRR312826-S18-L001	2017	USA	na	L1
MN073101	PRRSV-2018014209-S1	2018	USA	na	L1
MN073102	7705R-S1	2018	USA	na	L1
MN073103	014737Fib-S5-L001	na	USA	na	L1
MN073104	014737lu1A-S1-L001	na	USA	na	L1
MN073105	014737lu2A-S2-L001	na	USA	na	L1
MN073106	014737luC-S4-L001	na	USA	na	L1
MN073107	014737Ton-S6-L001	na	USA	na	L1
MN073108	014737luB-S3-L001	na	USA	na	L1
MN073109	br42321BC-S13-L001	na	USA	na	L1
MN073110	PRR41505-S4-L001	na	USA	na	L1
MN073111	PRR027983-S27-L001	2017	USA	na	L1
MN073112	PRR027984-S28-L001	2017	USA	na	L1
MN073113	PRR312823-S15-L001	2017	USA	na	L1
MN073114	52335PRRS-S10-L001	2016	USA	na	L1
MN073115	5988810PRRS-S5-L001	2016	USA	na	L1
MN073116	27981kf-S3-L001	2017	USA	na	L1

MN073117	PRR027982-S26-L001	2017	USA	na	L1
MN073118	PRR027981-S25-L001	2017	USA	na	L1
MN073119	5381R-S5-L001	2017	USA	na	L1
MN073120	5383R-S7-L001	2017	USA	na	L1
MN073121	5382R-S6-L001	2017	USA	na	L1
MN073122	6950-1R-S2	2018	USA	na	L1
MN073123	PRR312821-S13-L001	2017	USA	na	L1
MN073124	PRR312822-S14-L001	2017	USA	na	L1
MN073125	PRR312827-S19-L001	na	USA	na	L1
MN073126	016789PRR1-S11-L001	na	USA	na	L1
MN073127	7684R-S11	2018	USA	na	L1
MN073128	PRRSV-201704176-1-S16	na	USA	na	L7
MN073129	41761R-S15-L001	2018	USA	na	L7
MN073130	4115R-S1	2018	USA	na	L5
MN073131	1041R-S1	2018	USA	na	L5
MN073132	4512R-S7-L001	2018	USA	na	L5
MN073133	7473PRRS-S3-L001	2016	USA	na	L5
MN073134	PRR715665-S8-L001	na	USA	na	L5
MN073135	PRR715666-S9-L001	na	USA	na	L5
MN073136	397081R-S16-L001	2017	USA	na	L5
MN073137	397082R-S17-L001	2017	USA	na	L5
MN073138	7472PRRS-S2-L001	2016	USA	na	L5
MN073139	7471PRRS-S1-L001	2016	USA	na	L5
MN073140	lung23199-S4-L001	2017	USA	na	L5
MN073141	PRR1467916R-S6-L001	2017	USA	na	L5
MN073142	PRR146795R-S5-L001	2017	USA	na	L5
MN073143	PRR146795SA-S1-L001	2017	USA	na	L5
MN073144	PRR146795SQ-S3-L001	2017	USA	na	L5
MN073145	PRR1467916SA-S2-L001	2017	USA	na	L5
MN073146	PRR1467916SQ-S4-L001	2017	USA	na	L5
MN073147	PRRS35752R-S7-L001	2017	USA	na	L5
MN073148	PRR02760-S1-L001	2017	USA	na	L5
MN073149	PRR02760Q-S15-L001	2017	USA	na	L5
MN073150	lng26861I-S17-L001	2017	USA	na	L5
MN073152	PRR71566-4-S10	na	USA	na	L8
MN073153	PRR715664-S10-L001	na	USA	na	L8
MN073154	PRR715667-S11-L001	na	USA	na	L8
MN073155	PRR3-S3-L001	2016	USA	na	L8
MN073156	PRRSV-2017083451-S10	na	USA	na	L8
MN073157	3451R-S10-L001	2018	USA	na	L8
MN073158	2017021039-S12-L001	2017	USA	na	L8
MN073159	lung249621-S5-L001	2017	USA	na	L8
MN073160	lung249622-S6-L001	2017	USA	na	L8

MN073161	9985R-S8-L001	2017	USA	na	L8
MN073162	9981R-S4-L001	2017	USA	na	L8
MN073163	PRR40074-S3-L001	na	USA	na	L8
MN073164	serum24962-S7-L001	2017	USA	na	L8
MN073165	9983R-S6-L001	2017	USA	na	L8
MN073166	PRR80785GJ-S21-L001	na	USA	na	L8
MN073167	PRR80785GD-S22-L001	na	USA	na	L8
MN073168	562482PRRS-S4-L001	2016	USA	na	L8
MN073169	6660R-S5-L001	2017	USA	na	L8
MN073170	9986R-S9-L001	2017	USA	na	L8
MN073171	9987R-S10-L001	2017	USA	na	L8
MN073172	1504R-S1	2018	USA	na	L1
MN073173	8812-3R-S15	2018	USA	na	L1
MN073174	PRRSV-2018017613-S8	2018	USA	na	L1
MN073175	012574PRR-S1-L001	na	USA	na	L1
MN073176	012574VI-S2-L001	na	USA	na	L1
MN073177	191R-S18-L001	2017	USA	na	L1
MN073178	192R-S19-L001	2017	USA	na	L1
MN073179	PRRSVlung-S1-L001	2016	USA	na	L1
MN073180	7703R-S6	2018	USA	na	L1
MN073181	PRR80785	na	USA	na	L8
MN073182	PRR22334-1	na	USA	na	L1
MN175677	PRRSV2/USA/Lab3	2016	USA	na	L1
NC_001961	NC_001961	na	USA	na	L5
U87392	ATCC VR-2332	1992	USA	na	L5
FJ394029	07QN	2007	Viet<br>Nam	na	L8
JX512910	SRV07	2007	Viet<br>Nam	na	L8
KU842720	Hanvet1.vn	2010	Viet<br>Nam	na	L8

Supplementary Table 2. Reference strains information used in this study.

Accession number	Strain	Date	Country	Province/<br>States	Lineage
AB023782	Kitasato 93-1	na	Japan	na	L4
AB175696	Jiw1	na	Japan	Iwate	L4
AB175708	Jtg1	na	Japan	Tochigi	L4
AB175716	Aomori 93	na	Japan	Aomori	L4
AB175717	Chiba 14	na	Japan	Chiba	L4
AB175720	EDRD-8	na	Japan	Hokkaido	L4
AB175721	Gu922M	na	Japan	Gunma	L4
AB175722	Ibaraki 3	na	Japan	Ibaraki	L4
AB175712	Jyt1	na	Japan	Yamagata	L4
AB175714	Jyt3	na	Japan	Yamagata	L4
AF176462	PRRSV40	na	USA	Illinois	L9
DQ475064	PRRSV0000386	2004	na	na	L9
DQ476027	PRRSV0001584	2003	USA	Nebraska	L9
DQ476294	PRRSV0001940	2003	USA	Texas	L9
DQ476464	PRRSV0002160	2003	USA	North Carolina	L9
DQ477194	PRRSV0003049	2004	USA	Texas	L9
DQ477476	PRRSV0003401	na	na	na	L9
EU556160	2000-5424	na	na	na	L9
EU756048	PRRSV0000004469	2005	USA	Minnesota	L9
EU756993	PRRSV0000006345	2001	USA	Iowa	L9
EU757947	PRRSV0000007649	2005	USA	Iowa	L9
EU758266	PRRSV0000008140	2005	USA	Iowa	L9
JX014352	XJNJ-2-2	2011	China	Xinjiang	L9
JX014359	XJNJ-17-2	2011	China	Xinjiang	L9

Supplementary Table3. Recombination information of PRRSVs in 2019-2021.

Accession number	Strain name	Country	Major parent	Minor parent	Region 1			Region 2			Region 3			Region 4		
					Left	Right	Length	Left	Right	Length	Left	Right	Length	Left	Right	Length
MK429986	SWU/CD1/2018	China	L1	L5	7871	8111	240	12849	13296	447						
MK429985	SWU/YB2/2018	China	L1	L5	7931	8126	195	12847	13311	464						
MK429984	SWU/YB1/2018	China	L1	L5	7871	8111	240	12849	13301	452						
MK429983	SWU/MY6/2018	China	L1	L5	7890	8109	219	12840	13301	461						
MK429982	SWU/MY5/2018	China	L1	L5	7890	8109	219	12840	13301	461						
MK429981	SWU/MS3/2018	China	L1	L5	7871	8111	240	12829	13315	486						
MK429980	SWU/MS2/2018	China	L1	L5	7911	8111	200	12847	13301	454						
MK144543	SCya18	China	L1	L8	1	1685	1684	11093	11600	507	12001	12270	269	12468	14171	1703
MH588710	SDbz16-2	China	L1	L8	5414	6949	1535									
MF766472	HeN1501	China	L8	L5	12525	12875	350									
MN606304	JS18-3	China	L8	L1	1799	5019	3220									
MN660070	GXNN1839	China	L1	L5	7911	8113	202	12844	13300	456						
MN660067	GXNN1396-p3	China	L8	L9	12916	13141	225									
MK780825	SD110-1608	China	L8	L3	3889	6738	2849	11329	13067	1738	13792	15211	1419			
MK396384	GDsf1809	China	L1	L5	7842	8111	269	12890	13277	387						
MK396383	GDsf1808	China	L1	L5	7836	8117	281	12890	13285	395						
MK396382	GDsf1807	China	L1	L5	7835	8129	294	12898	13296	398						
MK396381	GDsf1806	China	L1	L5	7842	8117	275	12890	13285	395						
MK396380	GDsf1804	China	L1	L5	7842	8117	275									
MK396379	GDsf1802	China	L1	L8	6960	7441	481	7871	8752	881						
MK396378	GDsf1711	China	L1	L8	6954	7437	483	7851	8752	901						
MK396377	GDsf1710	China	L1	L8	6954	7437	483	7871	8752	881						
MK396376	GDsf1707	China	L1	L8	6954	7437	483	7871	8752	881						
	HB94-lun	China	L1	L8	5496	8132	2636									
	S145	China	L1	L5	7971	8104	133									
	S130-lym	China	L1	L5	7874	8104	230									
	C101-lun	China	L8	L5	12451	12634	183	12901	15898	2997						
	S039	China	L8	L1	1814	5772	3958	13827	14984	1157						
	H60-lun	China	L1	L8	1	2030	2029	5415	8173	2758						

	NA80-lun	China	L5	L8	1235	1495	260	2266	4401	2135	5809	6687	878	9093	12534	3441
	LN86-lun	China	L1	L5	11088	11661	573									
	H013	China	L1	L8	5901	6201	300	7122	8121	999						
	S043	China	L1	L8	1	1551	1550	5402	8151	2749						
	S136-lun	China	L1	L8	5940	6317	377	6761	8118	1357						
	H012	China	L1	L8	1	2025	2024	5504	9168	3664	14011	14251	240			
	S001-lun	China	L1	L5	7871	8116	245	12824	13308	484						
MF526896	GDQYQC2	China	L8	L3	11328	13843	2515	14568	15081	513						
MN119308	JS1810-195	China	L8	L6	4301	4451	150	5361	5560	199	12071	13041	970			
MN119309	XJ1904-39	China	L8	L1	2122	5756	3634	12281	15012	2731						
MN642099	SDqd1501	China	L8	L3	3899	6760	2861	11336	13105	1769	13827	15172	1345			
MN642102	SDwh1601	China	L8	L1	12344	12547	203	12836	15315	2479						
MN642105	SDyt1401	China	L8	L1	12312	12521	209	12820	15315	2495						
MT394494	GDhh1808	China	L8	L1	1569	5415	3846	12317	13866	1549	14319	15046	727			
MT416541	PRRSV2/CN/X2984/2018	China	L8	L5	8910	9590	680	13148	13357	209						
MT416542	PRRSV2/CN/N9185/2018	China	L8	L1	12021	14033	2012	14191	14600	409						
MT416543	PRRSV2/CN/F1228/2017	China	L8	L5	1972	2189	217									
MT708500	SD-YL1712	China	L8	L1	2145	4072	1927									
MT721741	PRRSV2/CN/GDDX/2018	China	L8	L5	8770	9590	820	13148	13357	209						
MZ579701	HBap4/2018	China	L8	L1	2030	5673	3643	6857	7921	1064	14848	15028	180			
MH651745	SD99-1606	China	L1	L5	7881	8121	240									
MN119305	SD1704-23	China	L1	L8	6798	7416	618	12880	13253	373						
MN119307	HN1804-2	China	L1	L8	5522	8141	2619									
MN642101	SDwh1403	China	L1	L5	7881	8127	246	12851	13331	480						
MN862433	FJDJQ-2018	China	L1	L8	1	1294	1293	4236	6394	2158	7259	9668	2409			
MT036897	FS-GD-02_FoShan_Guangdong_2016.01	China	L1	L8	6775	9794	3019	11081	11345	264						
MT036898	GDHZ_Huizhou	China	L1	L8	11348	11701	353									
MT036899	HZ1-3_Yunnan_20171	China	L1	L8	429	683	254									
MT075480	SC/DJY	China	L1	L8	7868	8944	1076	11095	11561	466	11864	12343	479			
MT165636	GD1909	China	L1	L8	1	1303	1302	4395	5984	1589	7821	8134	313			
MT394496	GDsc1808	China	L1	L5	7842	8121	279	12890	13285	395						
MT394497	GDsc1809	China	L1	L5	7842	8117	275	12893	13285	392						
MT409687	PRRSV2/CN/X4831/2018	China	L1	L8	6933	7451	518	7868	8251	383						
MT409688	PRRSV2/CN/X9830/2018	China	L1	L8	185	701	516	12788	13418	630	14241	14396	155			

MT409689	PRRSV2/CN/X4836/2018	China	L1	L8	6888	9516	2628	12486	12914	428	13113	13471	358			
MT409691	PRRSV2/CN/X4833/2018	China	L1	L8	1	1046	1045	12850	13007	157	13189	13418	229			
MT409692	PRRSV2/CN/X4839/2017	China	L1	L8	4855	5121	266									
MT416546	PRRSV2/CN/110713/2018	China	L1	L8	431	716	285	6945	7366	421	8291	8497	206			
MW531679	GXNN202004a	China	L1	L8	1	446	445	5721	6902	1181						
MW561594	GXNN202004	China	L1	L8	1	2024	2023	5812	8964	3152	11356	11561	205			
MW803134	PRRSV-China/SCcd2020/2020	China	L1	L8	4085	4594	509	5438	8149	2711	8774	8957	183	12661	13229	568
MZ342900	JS2020	China	L1	L8	5445	8239	2794	12728	13000	272	14323	14525	202			

Supplementary Table 4. Primers used to construct the HuN4 and HeB108 recombinant viruses.

Primer name	Primer Sequence (5'-3')
HeB- <i>Nhe</i> -F	AGGAACAGTGTTTAAACTGCTAGCCGCCAGCGGCTTGAC
HeB- <i>Nhe</i> -R	GTCAAGCCGCTGGCGGCTAGCAGTTTAAACACTGTTTCCT
HeB-HuN-F	CTCAGGTCCAATCATGAGGGGAAGAAGTCCAGGGTG
HeB-HuN-R	CACCCTGGACTTCTTCCCCTCATGATTGGACCTGAG
HeB-HuNp-F	GCTGTGCGAGAAAACTGGCAGACTGTTACCCCTTGC
HeB- HuNp-R	GCAAGGGGTAACAGTCTGCCAGTTTTCTCGCACAGC
HuN-HeB-F	CTCAGGTCCAATTATGAAGGGGAAGAAGTCCAGAATG
HuN-HeB-R	CATTCTGGACTTCTTCCCCTTCATAATTGGACCTGAG
HuN- <i>Nhe</i> -F	AGGAGCAGTGTTTAAACTGCTAGCCGCCAGCGGCTTG
HeB- <i>Asc</i> -R	TTTCCCTTTCTGGCGCGCCCGAAACGCATCATTGTAAT
HuN-HeBp-F	GCTGTGCGAGAAAACTGGCAAACACTGTTACCCCTTGT
HuN-HeBp-R	ACAAGGGGTAACAGTTTGCCAGTTTTCTCGCACAGC
